# Common Genetic Polymorphisms Influence Blood Biomarker Measurements in COPD

**DOI:** 10.1371/journal.pgen.1006011

**Published:** 2016-08-17

**Authors:** Wei Sun, Katerina Kechris, Sean Jacobson, M. Bradley Drummond, Gregory A. Hawkins, Jenny Yang, Ting-huei Chen, Pedro Miguel Quibrera, Wayne Anderson, R. Graham Barr, Patricia V. Basta, Eugene R. Bleecker, Terri Beaty, Richard Casaburi, Peter Castaldi, Michael H. Cho, Alejandro Comellas, James D. Crapo, Gerard Criner, Dawn Demeo, Stephanie A. Christenson, David J. Couper, Jeffrey L. Curtis, Claire M. Doerschuk, Christine M. Freeman, Natalia A. Gouskova, MeiLan K. Han, Nicola A. Hanania, Nadia N. Hansel, Craig P. Hersh, Eric A. Hoffman, Robert J. Kaner, Richard E. Kanner, Eric C. Kleerup, Sharon Lutz, Fernando J. Martinez, Deborah A. Meyers, Stephen P. Peters, Elizabeth A. Regan, Stephen I. Rennard, Mary Beth Scholand, Edwin K. Silverman, Prescott G. Woodruff, Wanda K. O’Neal, Russell P. Bowler, SPIROMICS Research Group, COPDGene Investigators

**Affiliations:** 1 Department of Biostatistics, University of North Carolina at Chapel Hill, Chapel Hill, North Carolina, United States of America; 2 Department of Biostatistics and Informatics, Colorado School of Public Health, University of Colorado Anschutz Medical Campus, Aurora, Colorado, United States of America; 3 National Jewish Health, Denver, Colorado, United States of America; 4 Division of Pulmonary, Critical Care and Sleep Medicine, Department of Medicine, Johns Hopkins University School of Medicine, Baltimore, Maryland, United States of America; 5 Center for Genomics and Personalized Medicine Research, Wake Forest School of Medicine, Winston-Salem, North Carolina, United States of America; 6 Collaborative Studies Coordinating Center, Gillings School of Global Public Health, University of North Carolina at Chapel Hill, Chapel Hill, North Carolina, United States of America; 7 Marsico Lung Institute/Cystic Fibrosis Research Center, Department of Medicine, Division of Pulmonary and Critical Care Medicine, University of North Carolina at Chapel Hill, Chapel Hill, North Carolina United States of America; 8 Department of Medicine, Columbia University Medical Center, New York, New York; Department of Epidemiology, Mailman School of Public Health at Columbia University, New York, New York, United States of America; 9 Department of Epidemiology, Gillings School of Global Public Health, University of North Carolina at Chapel Hill, Chapel Hill, North Carolina, United States of America; 10 Department of Epidemiology, Bloomberg School of Public Health, Johns Hopkins University,Baltimore, Maryland, United States of America; 11 Division of Respiratory and Critical Care Physiology and Medicine, Harbor- University of California at Los Angeles Medical Center, Torrance, California, United States of America; 12 Channing Division of Network Medicine, Department of Medicine, Brigham and Women's Hospital and Harvard Medical School, Boston, Massachusetts, United States of America; 13 Division of Pulmonary and Critical Care Medicine, University of Iowa, Iowa City, Iowa, United States of America; 14 Department of Medicine, Division of Pulmonary, Critical Care and Sleep Medicine, National Jewish Health, Denver, Colorado, United States of America; 15 Department of Thoracic Medicine and Surgery, Lewis Katz School of Medicine, Temple University, Philadelphia, Pennsylvania, United States of America; 16 Division of Pulmonary and Critical Care Medicine, Channing Division of Network Medicine, Brigham and Women's Hospital, Harvard Medical School, Boston, Massachusetts, United States of America; 17 Division of Pulmonary, Critical Care, Allergy, and Sleep Medicine, Department of Medicine, University of San Francisco Medical Center, University of California San Francisco, San Francisco, California, United States of America; 18 Division of Pulmonary and Critical Care Medicine, University of Michigan Health System, Ann Arbor, Michigan; VA Ann Arbor Healthcare System, Ann Arbor, Michigan, United States of America; 19 Division of Pulmonary and Critical Care Medicine, University of Michigan Health System, Ann Arbor, Michigan, United States of America; 20 Section of Pulmonary and Critical Care Medicine, Baylor College of Medicine, Houston, Texas, United States of America; 21 Division of Pulmonary and Critical Care Medicine, Johns Hopkins University School of Medicine, Baltimore, Maryland, United States of America; 22 Department of Radiology, Division of Physiologic Imaging, University of Iowa Hospitals and Clinics, Iowa City, Iowa, United States of America; 23 Department of Genetic Medicine, Weill Cornell Medical College, New York, New York, Department of Medicine, Division of Pulmonary and Critical Care Medicine, Weill Cornell Medical College, New York, New York, United States of America; 24 Department of Internal Medicine, Division of Pulmonary and Critical Care Medicine, University of Utah, Salt Lake City, Utah, United States of America; 25 Division of Pulmonary and Critical Care Medicine, Department of Medicine, David Geffen School of Medicine, University of California Los Angeles, Los Angeles, California, United States of America; 26 Department of Medicine, Weill Cornell Medical College, New York-Presbyterian Hospital/Weill Cornell Medical Center, New York, New York, United States of America; 27 Department of Medicine, Division of Pulmonary, Critical Care, Allergy and Immunologic Medicine, Wake Forest University School of Medicine, Winston-Salem, North Carolina, United States of America; 28 Department of Medicine, National Jewish Health, Denver, Colorado United States of America; 29 Division of Pulmonary and Critical Care Medicine, University of Nebraska, Omaha, Nebraska, United States of America; 30 Division of Pulmonary, Critical Care, Sleep and Allergy, Department of Medicine and Cardiovascular Research Institute, University of California San Francisco School of Medicine, San Francisco, California, United States of America; 31 Marsico Lung Institute/Cystic Fibrosis Research Center, Department of Medicine, University of North Carolina at Chapel Hill, Chapel Hill, North Carolina United States of America; 32 Department of Medicine, Division of Pulmonary Medicine, National Jewish Health, Denver, Colorado, United States of America; Georgia Institute of Technology, UNITED STATES

## Abstract

Implementing precision medicine for complex diseases such as chronic obstructive lung disease (COPD) will require extensive use of biomarkers and an in-depth understanding of how genetic, epigenetic, and environmental variations contribute to phenotypic diversity and disease progression. A meta-analysis from two large cohorts of current and former smokers with and without COPD [SPIROMICS (N = 750); COPDGene (N = 590)] was used to identify single nucleotide polymorphisms (SNPs) associated with measurement of 88 blood proteins (protein quantitative trait loci; pQTLs). PQTLs consistently replicated between the two cohorts. Features of pQTLs were compared to previously reported expression QTLs (eQTLs). Inference of causal relations of pQTL genotypes, biomarker measurements, and four clinical COPD phenotypes (airflow obstruction, emphysema, exacerbation history, and chronic bronchitis) were explored using conditional independence tests. We identified 527 highly significant (p < 8 X 10^−10^) pQTLs in 38 (43%) of blood proteins tested. Most pQTL SNPs were novel with low overlap to eQTL SNPs. The pQTL SNPs explained >10% of measured variation in 13 protein biomarkers, with a single SNP (rs7041; p = 10^−392^) explaining 71%-75% of the measured variation in vitamin D binding protein (gene = *GC*). Some of these pQTLs [*e*.*g*., pQTLs for VDBP, sRAGE (gene = *AGER*), surfactant protein D (gene = *SFTPD*), and TNFRSF10C] have been previously associated with COPD phenotypes. Most pQTLs were local (*cis*), but distant (*trans*) pQTL SNPs in the *ABO* blood group locus were the top pQTL SNPs for five proteins. The inclusion of pQTL SNPs improved the clinical predictive value for the established association of sRAGE and emphysema, and the explanation of variance (R^2^) for emphysema improved from 0.3 to 0.4 when the pQTL SNP was included in the model along with clinical covariates. Causal modeling provided insight into specific pQTL-disease relationships for airflow obstruction and emphysema. In conclusion, given the frequency of highly significant local pQTLs, the large amount of variance potentially explained by pQTL, and the differences observed between pQTLs and eQTLs SNPs, we recommend that protein biomarker-disease association studies take into account the potential effect of common local SNPs and that pQTLs be integrated along with eQTLs to uncover disease mechanisms. Large-scale blood biomarker studies would also benefit from close attention to the ABO blood group.

## Author Summary

Precision medicine is an emerging approach that takes into account variability in genes, gene and protein expression, environment and lifestyle. Recent advances in high-throughput genome-wide genotyping, genomics, and proteomics coupled with the creation of large, highly-phenotyped clinical cohorts now allows for integration of these molecular data sets at the individual level. Here we use genome-wide genotyping and blood measurements of 88 biomarkers in 1,340 subjects from two large NIH-supported clinical cohorts of smokers (SPIROMICS and COPDGene) to identify more than 300 novel DNA variants that influence measurement of blood protein levels (pQTLs). We find that many DNA variants explain a large portion of the variability of measured protein expression in blood. Furthermore, we show that integration of DNA variants with blood biomarker levels can improve the ability of predictive models to reflect the relationship between biomarker and disease features (*e*.*g*., emphysema) within chronic obstructive pulmonary disease (COPD).

## Introduction

Implementing precision medicine will require extensive use of biomarkers and in-depth understanding of the contributions of genetic, epigenetic, and environmental variation to phenotypic diversity and disease progression. Genome-wide association studies (GWAS) linking disease phenotypes to single nucleotide polymorphic (SNP) markers have successfully identified genes and pathways involved in complex phenotypes [1, 2]. GWAS are complemented by efforts of functional studies, such as the Genotype-Tissue Expression (GTEx) program [3], which seek to identify expression quantitative trait loci (eQTLs) linking SNP markers with mRNA expression [4]. Such eQTLs can illuminate relationships between genetic variation and disease phenotypes. However, genetic variants can also affect protein levels by mechanisms not detectable by eQTL analyses by altering post-transcriptional processes involving stability, translation, secretion and/or detection of the gene product. Few studies have been focused on the impact of genetic variation on large numbers of protein biomarkers in chronic diseases. However, the recent work in Battle et al., [5] suggests that variants affecting gene expression and protein level may be distinct, so identifying the genetic features that affect protein variation [protein quantitative trait loci (pQTLs)] and gene expression for disease-relevant biomarkers will be important.

To investigate the role of genetic variation on blood biomarkers and their relationship to a chronic disease, we examined genotyping-biomarker-clinical phenotype relationships in two independent, large, well-characterized cohorts of subjects at risk for chronic obstructive lung disease (COPD): Sub-Populations and InteRmediate Outcome Measures in COPD Study (SPIROMICS) [6] and COPDGene [7]. COPD is the third most common cause of death in developed countries [8] and has strong demographic (age, gender) and behavioral (*e*.*g*., smoking) risk factors, yet most smokers do not develop clinically important lung disease. Furthermore, COPD has several clinically important, but highly variable, phenotypes including extent and progression of airflow obstruction, loss of lung tissue (emphysema), frequent cough and sputum production (chronic bronchitis) and exacerbations. There have been many publications that have examined the relationship between blood biomarkers and these COPD phenotypes [9]. These biomarkers include both non-specific markers of inflammation (*e*.*g*., fibrinogen, C reactive protein, interleukin 6) as well as lung specific proteins (*e*.*g*., surfactant protein D, club cell 16) and other proteins [*e*.*g*., soluble receptor for advanced glycosylation endproducts (sRAGE), chemokine (C-C motif) ligand 18 (CCL18), and adiponectin]. Many of these biomarker studies have been replicated in independent cohorts and nearly all studies used antibody-based assays. The SPIROMICS and COPDGene biomarker efforts included many of these biomarkers as well as additional novel understudied biomarkers (S1 Table). Although some recent publications suggest that there may be important genetic associations for some blood protein measurements [10], there have been no studies that use multiple independent populations for large scale blood biomarkers, nor are there extensive evaluations on how the SNP-biomarker relationship influences prediction of disease phenotype. Because both SPIROMICS and COPDGene have complete genotyping data, some transcriptomic data, an identical panel of a large number of blood biomarkers, and extensive well-phenotyped clinical data, there is a unique opportunity to identify novel pQTLs and explore their influence on biomarker-disease relationships for COPD and its disease phenotypes.

## Materials and Methods

### Ethics statement

Written informed consent was received from all subjects. Collection and use of subject information and samples was approved at each clinical center (see S1 File) with the main approval from the IRB at National Jewish Health (HS-1883a) and the IRB at the University of North Carolina at Chapel Hill (10–0048)

### Study design, COPD phenotypes, and cohorts

#### Study design

This study reports a meta-analysis from two large cohorts of current and former smokers with and without COPD: SPIROMICS (ClinicalTrials.gov Identifier: NCT01969344) [6] and COPDGene (ClinicalTrials.gov Identifier: NCT00608764) [7]. For the present study, we analyzed non-Hispanic white (NHW) subjects who had both genotype and biomarker data. Although both of these large studies contain subjects of multiple ethnicities, because COPDGene only has the biomarkers used in this work measured on a NHW subset, the study population for SPIROMICS was also limited to NHW subjects. The selection of subjects accommodated the meta-analysis design chosen for the present work.

#### COPD phenotypes

For both studies, COPD was defined by spirometric evidence of airflow obstruction [post-bronchodilator forced expiratory volume at one second (FEV_1_)/forced vital capacity (FVC) <0.70], with severity defined as: mild or moderate (FEV_1_ >50% predicted) or severe (FEV_1_ ≤50% predicted). Chronic bronchitis was defined as self-reported chronic cough and sputum for at least three months in each of the two years prior to baseline. Emphysema was quantified by percent of lung voxels ≤-950 Hounsfield Units (% low attenuation areas: %LAA) on the full inspiratory CT scans. Exacerbations were defined as acute worsening of respiratory symptoms requiring treatment with oral corticosteroids and/or antibiotics, emergency room visit, or hospital admission [11].

#### Cohort description, SPIROMICS

Written informed consent was received from all subjects. Collection and use of subject information and samples was approved at each clinical center (see http://www2.cscc.unc.edu/spiromics/site-listing and S1 File) with the main approval from the IRB at the University of North Carolina at Chapel Hill (10–0048). Subjects were recruited into SPIROMICS in four strata [never smokers (stratum 1), smokers (≥20 packs/year) without COPD (stratum 2), smokers with mild/moderate COPD (stratum 3), smokers with severe COPD (stratum 4)] ([6] and http://www.spiromics.net). The data presented represents a 2012 interim analysis of baseline blood biomarkers and SNP genotyping. For the current study, only samples available at the time that the biomarker assays were conducted were used and these represent the first recruited subset of NHW SPIROMICS subjects. DNA from an overlapping, but not identical, subset of Stratum 2, 3, and 4 subjects was genotyped, and the overlapping subject data with both biomarker and genotype data were utilized. Investigator Dataset Release 3 (INV3), representing the first 1801 enrolled subjects, was utilized for capture of the clinical and demographic variables. Blood collection procedures (EDTA plasma and serum) at the baseline visit have been described [12].

#### Cohort description, COPDGene

Written informed consent was received from all subjects. Collection and use of subject information and samples was approved at each clinical center (see http://www.copdgene.org/locations and S1 File) with the main approval from the IRB at National Jewish Health (HS-1883a). This multi-center study of the genetic epidemiology of COPD enrolled 10,192 NHW and African-American individuals, aged 45–80 years with ≥10 pack-year smoking history and no exacerbation for >30 days [7]. The clinical dataset Final10000_Dataset_12MAR13 was used for the analysis, which represents the complete baseline dataset. Fresh frozen plasma was collected from 1839 non-fasting subjects (1599 NHW and 240 non-Hispanic Black) using a P100 tube (BD) at five COPDGene sites [National Jewish Health (N = 916), University of Iowa (N = 670), Los Angeles Biomedical Research Institute (N = 202), Temple University (N = 36), and Baylor Medical Center (N = 15)]. A subset of 602 NHW subjects was selected for comprehensive biomarker study as described [13]. The subset was selected to include a range of COPD severities from none to severe COPD. Of the 602 subjects, 590 had genome-wide genotyping, and the overlapping subjects were utilized for this study. The COPDGene data described in this manuscript is available through dbGaP phs000179.v4.p1 as well as GEO (accession GSE42057).

### Biomarker levels

114 candidate blood biomarkers (S1 Table) were initially evaluated using custom 13-panel multiplex assays (Myriad-RBM, Austin, TX). The 13-panel multiplexes were primarily selected because they contained at least one biomarker with known or putative links to COPD pathophysiology [12, 13]. Any analytes measured in addition to the pre-selected biomarkers were intended to be utilized for discovery purposes. Although reports of general assay performance are beyond the scope of the present work, details of a pilot study using the SPIROMICS samples on these assays is available that describes the coefficient of variation and reliability estimates for a majority of the analytes measured [12]. Details of the ability of the panels to detect the analyte above background [the lower limit of quantification (LLOQ)] are provided for both studies (S1 Table). Assay performance across the two cohorts was highly similar. Reproducibility of the platform was assessed for selected biomarkers (S1 Fig) using a subset of COPDGene subjects: for sRAGE using Quantikine human RAGE ELISA kit (R&D Systems, Minneapolis, MN) as previously described [14]; CRP (Roche Diagnostics, Mannheim, Germany) and fibrinogen (K-ASSAY fibrinogen test, Kamiya Biomedical Co., Seattle, WA, USA) levels were measured using immunoturbidometric assays as previously described [15]; surfactant protein D using colorimetric sandwich immunoassay method (BioVendor, Heidelberg, Germany) as previously described [16]. Additionally, serum from 63 SPIROMICS subjects who were either GG (N = 27) or TT (N = 36) at rs7041 were analyzed using a monoclonal antibody assay from R&D (Quanitkine ELISA kit) at the Clinical Research Unit Core Laboratory at Johns Hopkins. Polyclonal vitamin D binding protein measurements (ALPCO, Salem, NH) were performed in the same SPIROMICS subjects.

### Genotyping

#### SPIROMICS

This is the first reported use of SPIROMICS genotype data derived from OmniExpress plus Exome GeneChip (Illumina; San Diego, CA). The data presented utilizes a subset of SPIROMICS samples (in database release 1; n = 1143) in which we obtained Illumina OmniExpress plus Exome GeneChip genotypes. The cell lysate for DNA extraction was prepared at the clinical sites as per the SPIROMICS protocol, shipped to the UNC Biospecimen Processing Center for DNA extraction, and then provided to the Wake Forest Genotyping Core, where the DNA was hybridized to the chips.

For the present analysis, DNA hybridization was followed by several quality control steps, which were carried out in PLINK (http://pngu.mgh.harvard.edu/purcell/plink/) [17]. First, samples were evaluated for genetic versus reported/recorded sex, leading to removal of 5 samples due to discrepancy. Second, duplicated and/or related individuals were identified (7 pairs of related individuals were discovered with PI_HAT values > 0.1949). For these related individuals, the sample from the pair with the higher missing rate of genotype data was removed. After these clean up steps, principal component analysis (PCA) was conducted using common SNPs (N = 108,318) to identify individuals of divergent ancestry. HapMap3 populations (CEU—Utah residents with Northern and Western European ancestry from the CEPH collection; CHB—Han Chinese in Beijing, China; JPT—Japanese in Tokyo, YRI—Yoruba in Ibadan, Nigeria) were utilized in the ancestry analysis. For the cohort in the current analysis, we confirmed subject self-report as NHW by PCA. Of the genotyped samples, 856 were identified as NHW. From this subset, 761 were also evaluated in the biomarker dataset, and 11 of these subjects were dropped from the final dataset due to missing covariate values for these subjects. The final number utilized in these analyses was 750 NHW SPIROMICS subjects.

For SPIROMICS, missing genotype data rates were calculated, and SNPs with missing rate greater than 0.05 or minor allele frequency (MAF) < 0.01 were removed (2724 SNPs removed due to missing rate >0.05 and 225917 SNPs with MAF < 0.01 were removed). A Hardy Weinberg test statistic was calculated for each SNP and a test significance threshold of 0.001 was used to filter SNPs. Genotype principal components (PC’s) were then calculated after regressing out covariates site, age, gender, body mass index, smoking pack years, and current smoking status. Eigenvalues were calculated on the PCs to provide guidance for determining the number of genotype PCs to include in the final model (S2 Fig).

#### COPDGene

COPDGene subjects were of self-reported NHW or African-American ancestry, and genotyped using the HumanOmniExpress array (Illumina) [18]. Details on the processing of the COPDGene genotype data have been reported [18]. Briefly, genotyping was performed using the HumanOmniExpress array, and BeadStudio quality control, including reclustering on project samples was performed following Illumina guidelines. Subjects and markers with a call rate of < 95% were excluded. Population stratification exclusion and adjustment on self-reported white subjects was performed using EIGENSTRAT (EIGENSOFT Version 2.0).

### Statistical analysis

#### General features/overview

To accommodate the meta-analysis structure, statistical analysis was conducted separately within each study cohort followed by combined p-values meta-analysis. Regression analyses with covariates and genotype principal components were used to determine association of SNPs with analyte levels (pQTLs) [17]. Linear regression was used to identify pQTLs when percent of measurable values for the analyte was above 90%; otherwise the tobit model (also called the censored regression model) [19] was used. The set of independent pQTLs per analyte were identified using forward regression. Causal relations of SNP genotype, analyte levels, and disease phenotypes (*e*.*g*., chronic bronchitis, emphysema, exacerbation history, or airflow obstruction) were inferred by a conditional dependence testing approach that has been used in previous eQTL studies. Specific details of these analyses are provided below.

#### Handling of samples below LLOQ

Within each study (SPIROMICS and COPDGene), for each analyte, any measured values < LLOQ were imputed as half of LLOQ. LLOQ values specific to these assay runs were provided by Myriad-RBM. Then all measured values of each analyte were normalized by normal quantile transformation, as this type of rank-based transformation can effectively remove possible bias due to outliers or skewed distributions [20]. Regression analyses were conducted to determine the association of SNPs with analyte levels using the following criteria:

1. No analysis was conducted on analytes that had >90% of measurements <LLOQ. This criteria removed 28 analytes from the analysis.
2. Linear regression was conducted on analytes in which <10% of measurements < LLOQ.
3. For analytes with 10–90% of measured values <LLOQ, a censored regression (tobit) model was used (implemented using the censReg package in R). Because the data had first been normal quantile transformed, the normal distribution assumption of tobit model was automatically satisfied. The truncation value of tobit model was set as the minimum value above LLOQ (normal quantile transformation) minus a small constant (10^−10^). When such a biomarker is used as covariate for the Conditional Dependence analysis described below, values below the LLOQ for that biomarker were set to the conditional expectation [21].

#### Calculating pQTLs

In SPIROMICS, the following covariates were used for pQTL mapping (either linear or tobit model): genotype PC1, biomarker PC1, sites, sex, age, BMI, smoking pack years, current smoker status (0/1). In COPDGene, the following covariates were used for pQTL mapping (either linear or tobit model): genotype PC1—PC5, sites, sex, age, BMI, smoking pack years and current smoker status (0/1). We took this approach based on an initial PC analysis of the biomarker data across subjects from both cohorts. The model for SPIROMICS, but not COPDGene, included a biomarker principal component (PC1). (S2 Fig). For COPDGene, the first biomarker principal component was highly correlated with the other covariates (sex, age, BMI, etc.). By contrast, in SPIROMICS, the first biomarker PC was not associated with any of the covariates, indicating that there was additional structure in the data that needed to be adjusted for by including biomarker PC1; subsequent PCs were not included because they were either associated with other covariates or explained only a relatively small percentage of the variability. All pQTL analysis was performed by either PLINK (v 1.9; http://pngu.mgh.harvard.edu/~purcell/plink/, for linear regression) or censReg function of R package censReg (for tobit model).

We conducted meta-analysis combining the results of SPIROMICS and COPDGene studies using Stouffer's Z-score method adjusting for direction of effect. Specifically, let Φ and Φ^-1^ be cumulative distribution function (CDF) and inverse CDF of standard normal distribution. Let β_1_ and β_2_ be the regression coefficients from COPDGene and SPIROMICS studies, respectively, and let p_1_ and p_2_ be the corresponding p-values from COPDGene and SPIROMICS studies, respectively. Then the combined Z-statistic and meta p-value weighted by the sample sizes of the respective study is $Z=(\sqrt{n_{1}}z_{1}+\sqrt{n_{2}}z_{2})/(\sqrt{n_{1}}+\sqrt{n_{2}})$.

where *z*_1_ = *sign*(*β*_1_)|*Φ*^−1^(*p*_1_/2)| and *z*_2_ = *sign*(*β*_2_)|*Φ*^−1^(*p*_2_/2)|. Then, the meta-analysis p-value is 2*Φ*(−|*Z*|).

#### Recursive conditioning

The set of independent pQTLs per analyte were identified using a forward regression approach. If *K* SNPs were associated with an analyte with p-values smaller than 10^−8^, meta-p-values were calculated for each of the *K*-1 SNPs conditioning on the top SNP identified from meta-analysis. The SNP with the smallest meta-p-value was considered as an independent pQTL if the p-value < 0.05/(*K*-1), where 0.05/(*K*-1) was the p-value threshold by Bonferroni correction. We applied this procedure iteratively until the smallest meta-p-value was larger than 0.05/*T*, where *T* is the number of remaining SNPs.

#### Effect of blood cell counts on pQTLs

We also evaluated whether the pQTLs would be significantly affected by the cellular composition of the blood. Complete cell counts were only available for the SPIROMICS cohort, so we repeated the pQTL analysis adding cell counts of neutrophil, lymphocyte, monocyte, eosinophil, basophil, red blood cells, and platelet as covariates in the models. For either all possible (SNP, analyte) pairs or only those pairs corresponding to significant pQTLs, the concordance between the pQTL p-values with and without blood cell counts as covariates were tested in SPIROMICS cohort, but not COPDGene, in which cell counts were not available.

#### Studying causal relations by assessing (conditional) dependence

We adopted an approach used in previous eQTL studies to infer causal relations of a trio of SNP, biomarker, and disease phenotype. We assume any associations between SNP genotype and protein levels or disease phenotypes implies a causal relation that SNP genotype alterations causes changes in protein levels or disease phenotype. This is assumption can be justified by Mendelian Randomization, which argues that the passing of DNA alleles to offspring can be considered as a randomized experiment and causal relations can be inferred from the randomized experiment. Such inference of causal relation by Mendelian Randomization is also consistent with our intuition that genetic variation causes molecular or phenotypic changes rather than vice versa. Given this assumption on the causal relation between SNP and biomarker/disease phenotypes, different models involving a SNP, a biomarker, and a disease phenotype can be distinguished because these models encode different types of conditional independence information, and thus have different likelihoods. This approach has been used in previous studies, implemented by comparing different models based on their likelihoods [22, 23]. Later more rigorous statistical arguments have been established to compare different types of causal relations by testing (conditional) dependence [24–27] or likelihood ratio test [28]. We adopted the approach of testing (conditional) dependence in our study.

We seek to classify the relations of a trio of SNP, biomarker, and disease phenotype into five categories: causal, reactive, independent, collide, and complete. Some trios may not fall into any of these categories and they are classified as other. A causal model (SNP → biomarker → disease) would suggest a SNP’s effect on disease is mediated by a biomarker, and thus conditioning on that biomarker, SNP genotype is independent with disease. A reactive model (SNP → disease → biomarker) would suggest that a SNP’s effect on a biomarker is mediated by disease, and thus conditioning on disease, SNP genotype is independent with biomarker. In an independent model (biomarker ← SNP → disease), a pQTL SNP affects biomarker and disease separately and given SNP genotype, disease is independent with biomarker. In a collide model (SNP → biomarker ← disease), the abundance of a biomarker is affected by a SNP as well as disease, and there is no direct relation between the SNP and disease; however, SNP genotype and disease are dependent with each other conditioning on the biomarker. The complete model allows all possible relations of the three variable and each of the aforementioned models can be derived from the complete model after adding certain constraints on dependence or conditional dependence relation. The “collide” relationship is well known in graphical model studies [29], however, previous eQTL studies did not explore this model because they focused only on SNPs associated with disease phenotypes.

To examine conditional dependence between a trio of SNP, biomarker, and disease phenotype, we performed a series of linear or logistic regressions with a continuous disease phenotype (emphysema or FEV_1_% predicted) or a binary disease phenotype (chronic bronchitis or exacerbations) as response variable, as well as additional linear regression or tobit regression with biomarker as response variable. We assessed the conditional dependence of two variables by testing the hypothesis whether a slope parameter was 0. More specifically, we obtained p-values for a particular test from both SPIROMICS and COPDGene studies and combined them using the same meta-analysis approach used to calculate pQTLs (see above). Finally, we say a slope parameter is different from 0 [*i*.*e*., (conditional) dependence] if the meta-p-value is smaller than 0.01. A specific causal relation can be inferred based on a set of conditional dependence testing results.

For our eQTL analysis, this series of regressions were also fit using the trio for SNP, haptoglobin biomarker and haptoglobin gene expression to determine the conditional relationships. In this case, the models were only fit on the 102 subjects from COPDGene having both biomarker and gene expression data.

### Exploring pQTL features

pQTL features were characterized by: (1) Ensembl Variant Effect Predictor (VEP) [30]; (2) GWAS catalog [31]; and (3) comparison with gene expression QTLs (eQTLs) using subset of COPDGene blood microarrays [20, 32]. Details are provided below:

#### Variant effect predictor

We employed the Ensembl Variant Effect Predictor (VEP) tool to examine the consequences and locations of SNPs, using the “most severe consequence per variant” filter and genome version GRCh38.

#### GWAS catalog

The catalog of GWAS was obtained from NHGRI [31] containing 19,469 records (Feb 2015). For GWAS-pQTL SNP overlap, only unique entries by disease and publication were counted. Linkage disequilibrium (LD) information for the pQTL SNPs were obtained from LocusZoom [33] or HaploReg [34].

#### Defining relationship between pQTLs and eQTLs

Biomarkers were first mapped to gene identifiers and then to Affymetrix HGU133 plus 2 probe set symbols using Ensembl BioMart (www.ensembl.org/biomart). To examine biomarker-gene expression correlation, only the 80 biomarkers with <10% of measurements below the LLOQ were used. On average, these 80 biomarkers were encoded by genes with 2–3 Affymetrix probesets each. Overall, 199 probe sets were evaluated on n = 103 subjects with both gene expression and biomarker levels available for COPDGene. For the eQTL analysis, gene expression from all 131 NHW subjects from [32] were used with the same model as the pQTL analysis. For the 38 biomarkers with significant pQTL, 75 probesets corresponding to the genes encoding the biomarkers were used for a genome-wide eQTL analysis. The resulting eQTL were compared with the pQTL to identify if the same pQTL SNP is associated with both gene expression and protein levels for the biomarker. However, due to the loss of power with the smaller sample size for gene expression and to examine overall trends of variant effects for eQTL SNPs, we used a threshold of p-value < 10^−7^. This is larger than the pQTL threshold but would still correspond to the genome-wide significance threshold for local eQTL.

## Results

### Study subjects

Demographic and clinical characteristics of subjects from the SPIROMICS (n = 750) and COPDGene (n = 590) cohorts, including disease phenotypes, are shown (Table 1; S3 Fig). These NHW subjects were representative of NHWs in the parent cohorts (S2 Table).

**Table 1:** Demographics of participants by cohort at study entry^*^.

	SPIROMICS				COPDGene			
Characteristic	Overall	Current or former smokers without COPD	Mild or Moderate COPD	Severe COPD	Overall	Current or former smokers without COPD	Mild or Moderate COPD	Severe COPD
No. of participants	750	206	367	177	590	242	150	198
Age	66.5 ± 7.9	65.1 ± 9.0	67.5 ± 7.4	66.1 ± 7.1	63.7 ±8.6	61.2 ± 8.4	65.1 ± 8.9	65.6 ± 7.8
Male gender (%)	408 (54)	89 (43)	222 (60)	97 (55)	304 (52)	120 (50)	75 (50)	109 (55)
Current Smoker (%)	230 (31)	68 (33)	123 (34)	39 (22)	142 (24)	63 (26)	46 (31)	33 (17)
BMI	27.7 ± 5.0	28.3 ± 5.1	27.7 ± 5.0	26.8 ± 4.8	28.3 ± 5.6	29.0 ± 5.3	28.8 ±5.6	27.1 ± 5.6
Smoking pack-year	52.6 ± 24.4	45.2 ± 23.1	55.6 ± 25.5	54.9 ± 21.7	47.5 ± 26.6	38.2 ± 22.6	51.3 ± 27.7	55.7 ± 27.2
Emphysema (%)	8.7 ± 10.3)	1.9 ± 2.1	7.3 ± 7.4	19.6 ± 12.6	9.6 ± 11.8	2.3 ± 2.6	7.5 ±7.7	20.6 ±13.2
FEV_1_% predicted	71.0 ± 25.4	94.4 ± 13.7	75.0 ±15.8	35.7 ± 9.3	68.2 ± 29.8	98.1 ± 12.9	65.0 ±9.1	33.9 ± 10.3
FEV_1_/FVC	0.6 ±0.2	0.8 ±0.1	0.6 ±0.1	0.4 ±0.1	0.6 ± 0.2	0.8 ± 0.0	0.6 ± 0.1	0.4 ± 0.1
Exacerbations in prior 12 mo.	0.3 ± 0.8	0.2 ± 0.6	0.2 ± 0.7	0.6 ± 1.1	0.6 ± 1.2	0.2 ± 0.6	0.7 ± 1.2	1.1 ± 1.4
Chronic Bronchitis (%)	219 (29)	46 (22)	108 (29)	65 (37)	113 (19)	26 (11)	34 (23)	53 (27)

* means ± standard deviations.; BMI–body mass index; FEV_1_—forced expiratory volume at 1 second; FVC–forced vital capacity; Based on data retrieved August 19, 2013 (SPIROMICS Investigator Dataset Release INV3) and March 12, 2014 (COPDGene); see methods for definition of COPD severity.

### Identification of SNPs associated with blood biomarkers

**Fig 1:**
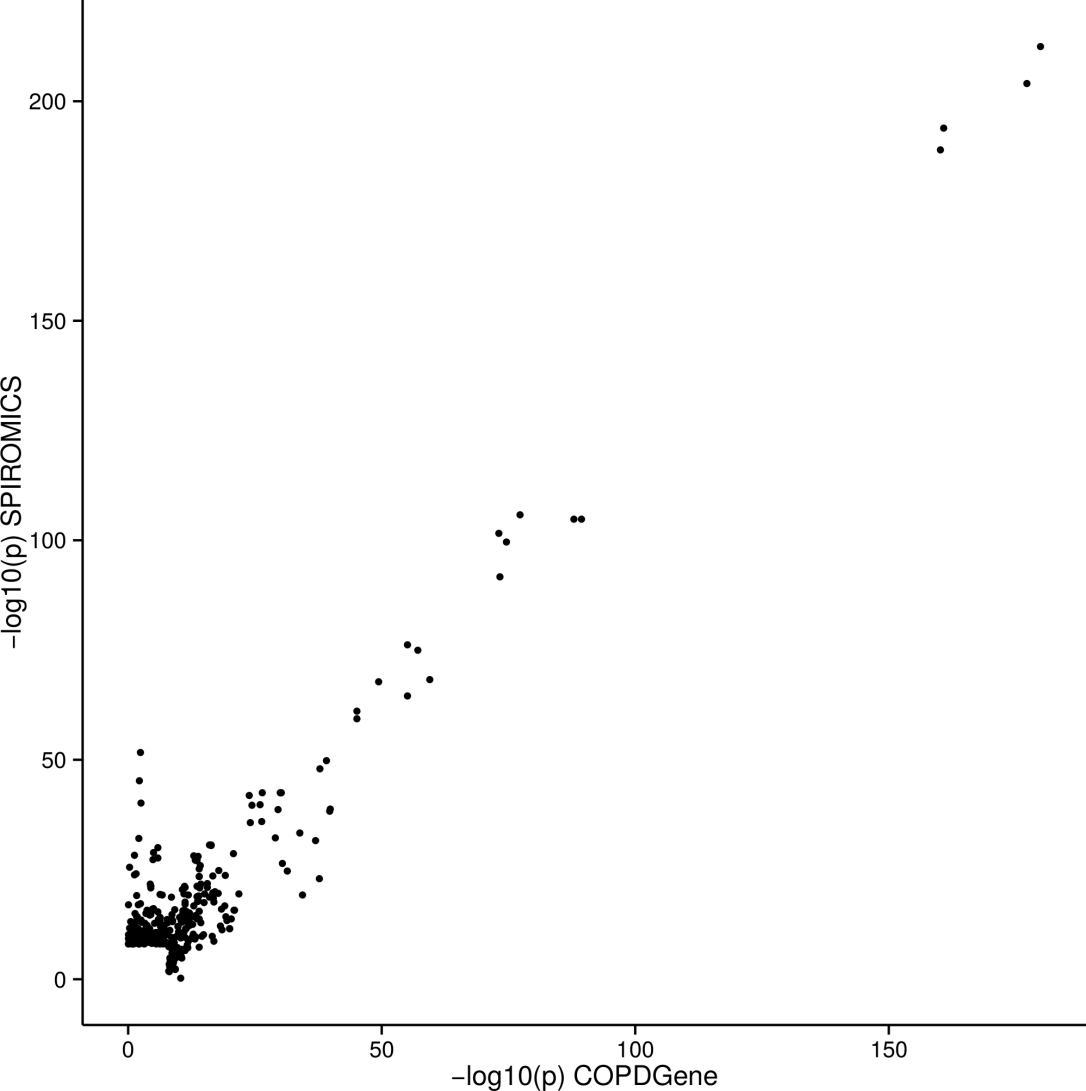
Comparison of–log10 p values for pQTL SNPs in SPIROMICS and COPDGene cohorts. The–log_10_(P value) of each pQTL SNP is plotted on the x-axis for COPDGene and the y-axis for SPIROMICS. 164 of 182 significant pQTLs in COPDGene were replicated in SPIROMICS at a P < (0.05/182 to correct for multiple tests). 209 of 290 significant pQTLs in SPIROMICS were replicated in COPDGene at a P < (0.05/290 to correct for multiple tests). See also S3 Table.

**Fig 2:**
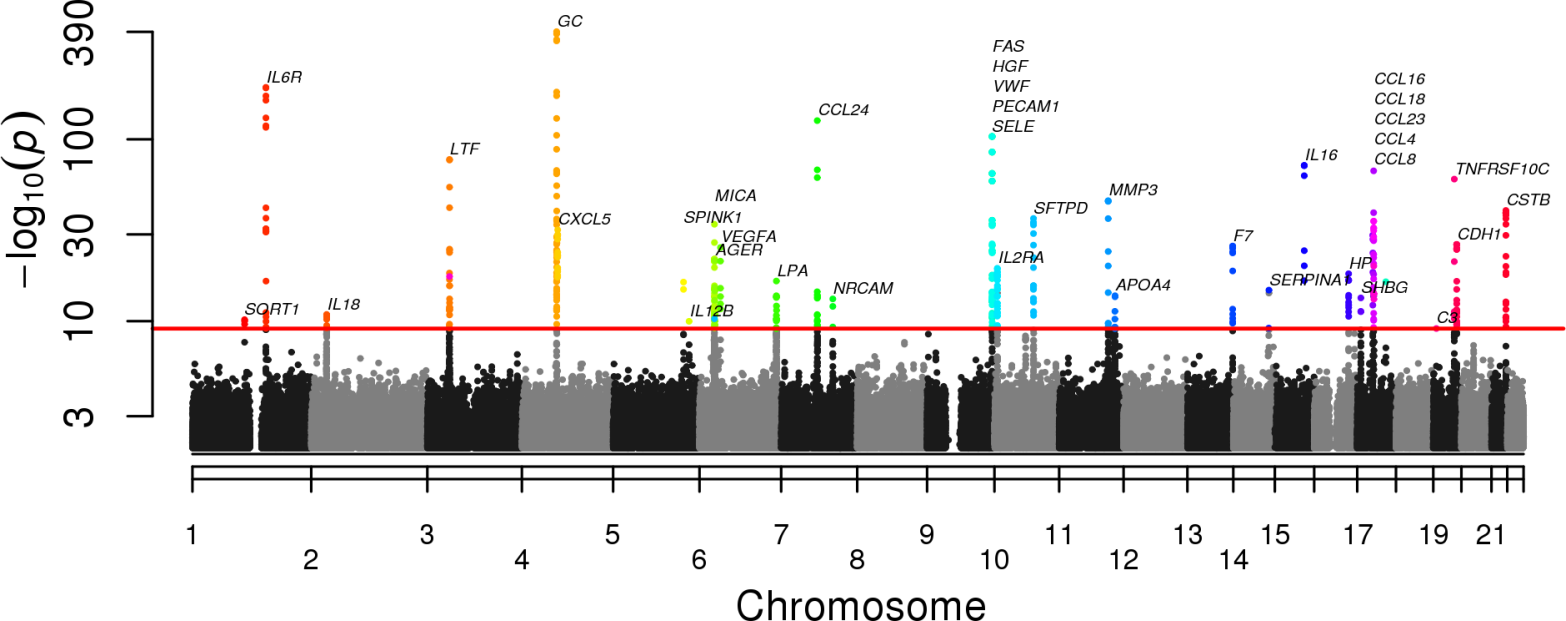
Genome wide associations between single nucleotide polymorphisms (SNPs) and blood biomarkers. Combined Manhattan plots show pQTL SNPs by chromosomal location for 38 biomarkers with at least one SNP significant at genome wide significance after adjustment for multiple testing (red line). The -10logP values are shown using results from a meta-analysis of both SPIROMICS and COPDGene SNPs. The abbreviation for the biomarker associated with the pQTL SNP can be found in S1 Table.

At a significance level of 8 X 10^−10^ we identified 290 pQTLs in the SPIROMICS cohort and 182 pQTLs in the COPDGene cohort (S3 Table). Many of the pQTLs SNPs were replicated between cohorts (Fig 1; S3 Table). Because of the similarity of the two studies in terms of sample size and subject characteristics as well as good replication of pQTLs between these two studies, we used a meta-analysis to increase power for finding pQTLs. Weighted meta-analysis identified 527 pQTL SNPs in 38 (44%) of the biomarkers (S4 Table) meeting genome-wide significance with Bonferroni correction for multiple testing of SNPs and biomarkers (P <8 X 10^−10^; Fig 2). The most significant independent pQTL SNP was rs7041 (P = 10^−392^) in *GC* (vitamin D binding protein—VDBP) on chromosome 4. Thirty-seven other biomarkers had significant pQTL SNPs (Table 2); corresponding Manhattan plots, Q-Q plots, and LocusZoom plots are shown for each individual analyte that had an associated pQTL (S4 Fig). Two or more independent pQTL SNPs were identified in 26 of 38 biomarkers using recursive conditioning (S5 Table).

**Table 2:** Summary of pQTLs by measured biomarker.

Analyte gene			Number of pQTLs^¶^			Top pQTL SNP†						
Analyte protein name {alternative name(s)}^‡^	Name	Chr	Local	Dis-tant	Multiple Independent	rs#	MAF SPIROMICS	MAF COPDGene	P-value	Chr	Novel^ƪ^	Function
Advanced glycosylation end product-specific receptor {RAGE}	*AGER*	6	10			rs2070600†	0.05	0.04	7 X 10^−22^	6	GWAS	mis
Apolipoprotein A-IV {Apo A-IV}	*APOA4*	11	10		yes	rs4938353	0.19	0.18	4 X 10^−14^	11	LD-GWAS	3'
Complement component 3	*C3*	19	1			rs2230203	0.20	0.18	1 X 10^−9^	19	Novel	Syn
Chemokine (C-C motif) ligand 16 (pulmonary and activation-regulated)	*CCL16*	17	6		yes	rs11080369	0.06	0.07	8 X 10^−67^	17	Novel	NA
C-C motif chemokine 18	*CCL18*	17	7		yes	rs854469	0.13	0.13	2 X 10^−35^	17	Novel	NA
Chemokine (C-C motif) ligand 23 {Myeloid Progenitor Inhibitory Factor 1; MPIF-1}	*CCL23*	17	11		yes	rs1617208	0.18	0.19	5 X 10^−29^	17	Novel	NA
Chemokine (C-C motif) ligand 24 {Eotaxin-2}	*CCL24*	7	14		yes	rs10755885	0.09	0.10	1 X 10^−126^	7	Novel	up
Chemokine (C-C motif) ligand 4 {Macrophage Inflammatory Protein-1 ß; MIP-1 ß}	*CCL4*	17	4	1	yes	rs3213636	0.20	0.19	6 X 10^−21^	17	Novel	NA
Chemokine (C-C motif) ligand 8 {Monocyte Chemotactic Protein 2; MCP-2}	*CCL8*	17	7		yes	rs3848464	0.10	0.10	1 X 10^−29^	17	Novel	inter
Cadherin-1 {E-cadherin (epithelial)}	*CDH1*	16		29	yes	rs516246	0.50	0.48	4 X 10^−27^	19	GWAS	int
Cystatin-B	*CSTB*	21	24		yes	rs1041456	0.42	0.43	9 X 10^−42^	21	Novel	up
C-X-C motif chemokine 5 {Epithelial-Derived Neutrophil-Activating Protein 78; ENA-78)}	*CXCL5*	4	14			rs425535	0.12	0.12	1 X 10^−33^	4	Novel	syn
Coagulation factor VII	*F7*	13	12		yes	rs10665	0.12	0.13	1 X 10^−26^	13	GWAS	3'
Tumor necrosis factor receptor superfamily member 6 {FASLG Receptor; CD95}	*FAS*	10		6	yes	rs687289	0.31	0.35	1 X 10^−23^	9	GWAS	int
Vitamin D-binding protein	*GC*	4	57		yes	rs7041†	0.42	0.44	1 X 10^−389^	4	GWAS	NA
Hepatocyte growth factor	*HGF*	7		15	yes	rs687289	0.31	0.35	3 X 10^−43^	9	GWAS	int
Haptoglobin	*HP*	16	11			rs1424241	0.17	0.18	6 X 10^−19^	16	Novel	int
Interleukin-12 subunit p40 {IL-12p40}	*IL12B*	5	1		yes	rs10045431	0.29	0.28	2 X 10^−10^	5	GWAS	inter
Interleukin-16	*IL16*	15	7			rs1803275	0.09	0.08	2 X 10^−72^	15	Novel	syn
Interleukin-18	*IL18*	11		6		rs7577696	0.40	0.41	9 X 10^−12^	2	GWAS	inter
Interleukin-23A	*IL23A*	12		1	yes	rs10665	0.12	0.13	5 X 10^−10^	13	GWAS	3'
Interleukin-2 receptor subunit alpha	*IL2RA*	10	26		yes	rs12722489	0.14	0.16	2 X 10^−20^	10	GWAS	int
Interleukin-6 receptor subunit alpha	*IL6R*	1	26		yes	rs8192284	0.40	0.40	9 X 10^−193^	1	GWAS	NA
Apolipoprotein(a)	*LPA*	6	19		yes	rs9457925	0.02	0.02	5 X 10^−18^	6	LD-GWAS	int
Lactotransferrin	*LTF*	3	23			rs11707471	0.32	0.31	2 X 10^−77^	3	Novel	int
MHC class I polypeptide-related sequence A	*MICA*	6	60			rs2256175	0.48	0.47	9 X 10^−34^	6	Novel	int
Stromelysin-1 {Matrix Metalloproteinase-3; MMP-3}	*MMP3*	11	10			rs645419	0.49	0.49	5 X 10^−47^	11	LD-GWAS	up
Neuronal cell adhesion molecule	*NRCAM*	7	3		yes	rs10487851	0.30	0.30	8 X 10^−14^	7	Novel	int
Platelet endothelial cell adhesion molecule	*PECAM1*	17	1	18	yes	rs507666	0.18	0.20	3 X 10^−57^	9	GWAS	int
E-selectin	*SELE*	1		29	yes	rs507666	0.18	0.20	4 X 10^−104^	9	GWAS	int
Alpha-1-antitrypsin	*SERPINA1*	14	3		yes	rs4905179	0.20	0.21	2 X 10^−15^	14	LD-GWAS	inter
Pulmonary surfactant-associated protein D {SP-D}	*SFTPD*	10	22	1	yes	rs2146192	0.11	0.10	4 X 10^−37^	10	Novel	int
Sex hormone-binding globulin	*SHBG*	17	3		yes	rs727428	0.42	0.42	3 X 10^−14^	17	GWAS	down
Sortilin	*SORT1*	1	3			rs7528419	0.22	0.22	1 X 10^−10^	1	GWAS	3'
Pancreatic secretory trypsin inhibitor {TATI}	*SPINK1*	5	2			rs6580502	0.41	0.40	2 X 10^−16^	5	Novel	int
Tumor necrosis factor receptor superfamily member 10C {TNF-Related Apoptosis-Inducing Ligand Receptor 3; TRAIL-R3)}	*TNFRSF10C*	8		5		rs4760	0.16	0.16	1 X 10^−60^	19	Novel	mis
Vascular endothelial growth factor A	*VEGFA*	6	6			rs7767396	0.47	0.47	5 X 10^−26^	6	LD-GWAS	inter
von Willebrand factor	*VWF*	12		13		rs687289	0.31	0.35	5 X 10^−36^	9	GWAS	int

†indicates that the analyte associated with the SNP has been associated with obstructive lung disease or emphysema (PubMed accession 23947473, 23267696 for AGER and 24857306, 21228423, 19996341 for GC).

^¶^Local SNPs are defined as within 1 Mb of the analyte gene; distant (*trans*) SNPs are denoted by red.

^‡^ Protein names are UniProKB/Swiss-Prot suggested names.

^ƪ^Novel SNPs are defined as not listed in the *Catalog of Published Genome-Wide Association Studies* (GWAS) as of May 8, 2015, not listed in LD with any GWAS catalog SNPs (LD-GWAS), and not found on PubMed Search associated with analyte levels or disease phenotypes (see S3 Table); not all non-novel pQTL SNPs are previously linked to analyte levels (some are disease associations). Multiple Independent pQTL SNPs are listed in S4 Table. SPIROMICS/COPDGene. Chr = chromosome. Functional annotation of SNPs (variant effect predictor): up (upstream gene variant); 5’ (5’ untranslated region); syn (synonymous variant); mis (missense); spl (splice region); int (intron); exon (non-coding exon variant); mis (missense); 3’ (3’ untranslated region); up (upstream), down (downstream); inter (intergenetic).

**Fig 3:**
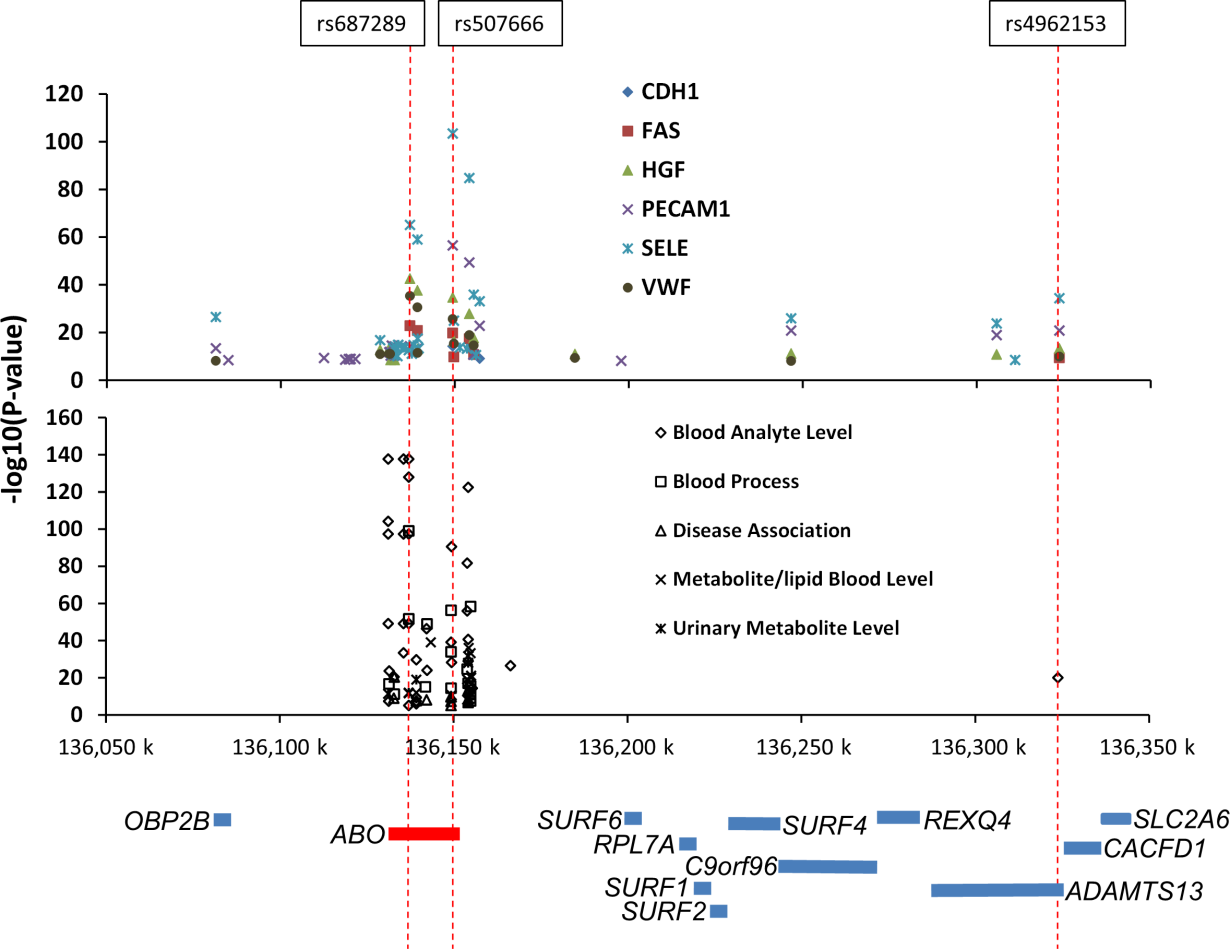
SNPs in *ABO* are the strongly associated with many blood biomarker levels as well as other non-blood analyte measurements. Trans pQTLs in the ABO region (shown in schematic form below the plots) were common in this study (top panel) and in published studies (GWAS Catalog). The rs687289 or rs507666 SNPs in the *ABO* blood group locus on chromosome 9, which encodes alpha 1-3-N-acetylgalactosaminyltransferase, are a major determinant of ABO blood type. In this study, these SNPs were the strongest pQTLs for 6 blood biomarkers, all distant (*trans*) from their biomarker genes. Other biologic features (such as clotting time), metabolites (proteins, lipids, hormones), and urinary features have been noted to have strong association with this locus (see [10, 35–73]).

To determine whether pQTLs SNPs were local (*cis*) or distant (*trans)*, we examined proximity of each SNP to its assigned biomarker gene. The majority (76%) of pQTL SNPs were local (S5 Fig; S4 Table). However, distant pQTLs were observed for eleven biomarkers, and nine biomarkers had a distant pQTL SNP as their most significant pQTL (S2 Table). Five biomarkers had their most significant pQTL SNPs (either rs687289 or rs507666) in the *ABO* blood group locus on chromosome 9, which encodes alpha 1-3-N-acetylgalactosaminyltransferase, a major determinant of ABO blood type. This SNP is in the same genetic region as other QTLs and disease associations reported from a wide variety of a sources, including metabolites from the urine (Fig 3). An additional region on chromosome 19 contained distant pQTLs for more than one biomarker (S4 Table). The pQTLs represented SNPs with a broad range of minor allele frequencies (MAF) with distributions of MAFs of pQTL SNPs similar to all SNPs studied (S6 Fig).

**Fig 4:**
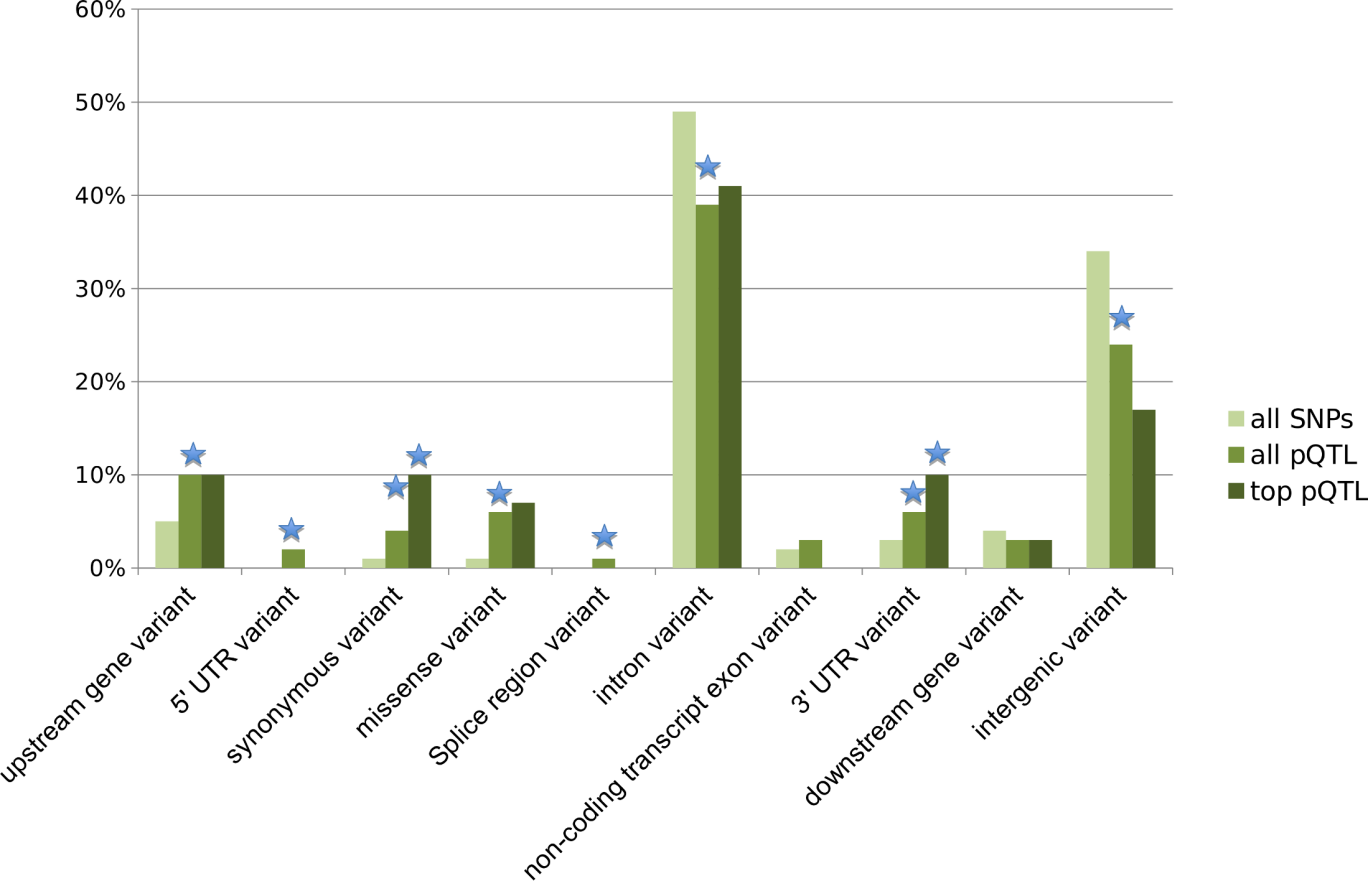
Consequences of pQTL SNPs. We examined the most significant SNP for each biomarker (top pQTL) and all 590 significant pQTL SNPs (all pQTLs), compared to all 664,913 SNPs (all SNPs) used for testing with the Ensemble Variant Effect Predictor (release 78). Upstream refers to within 5 kb and downstream refers to more than 5kb distant. All pQTL SNPs were enriched for missense, synonymous, upstream and 3′ UTR variants compared to all SNPs tested on the genotyping platform, while pQTL SNPs occurred less frequently in introns and intergenic regions (binomial test p-value < 0.05 starred in blue). Most of these variant classes showed additional enrichment or reduction for the top pQTL SNPs.

Using VEP, we found intronic SNPs to be the most represented pQTL SNP category (43%), followed by intergenic variants (22%); however, missense variants showed the most significant enrichment (P<10^−12^) compared to all SNPs on the genotyping platform (Fig 4). Importantly, pQTLs were robust and concordant across the two source cohorts (S4 Table; S7 Fig).

### Biologic significance of pQTL SNPs

**Fig 5:**
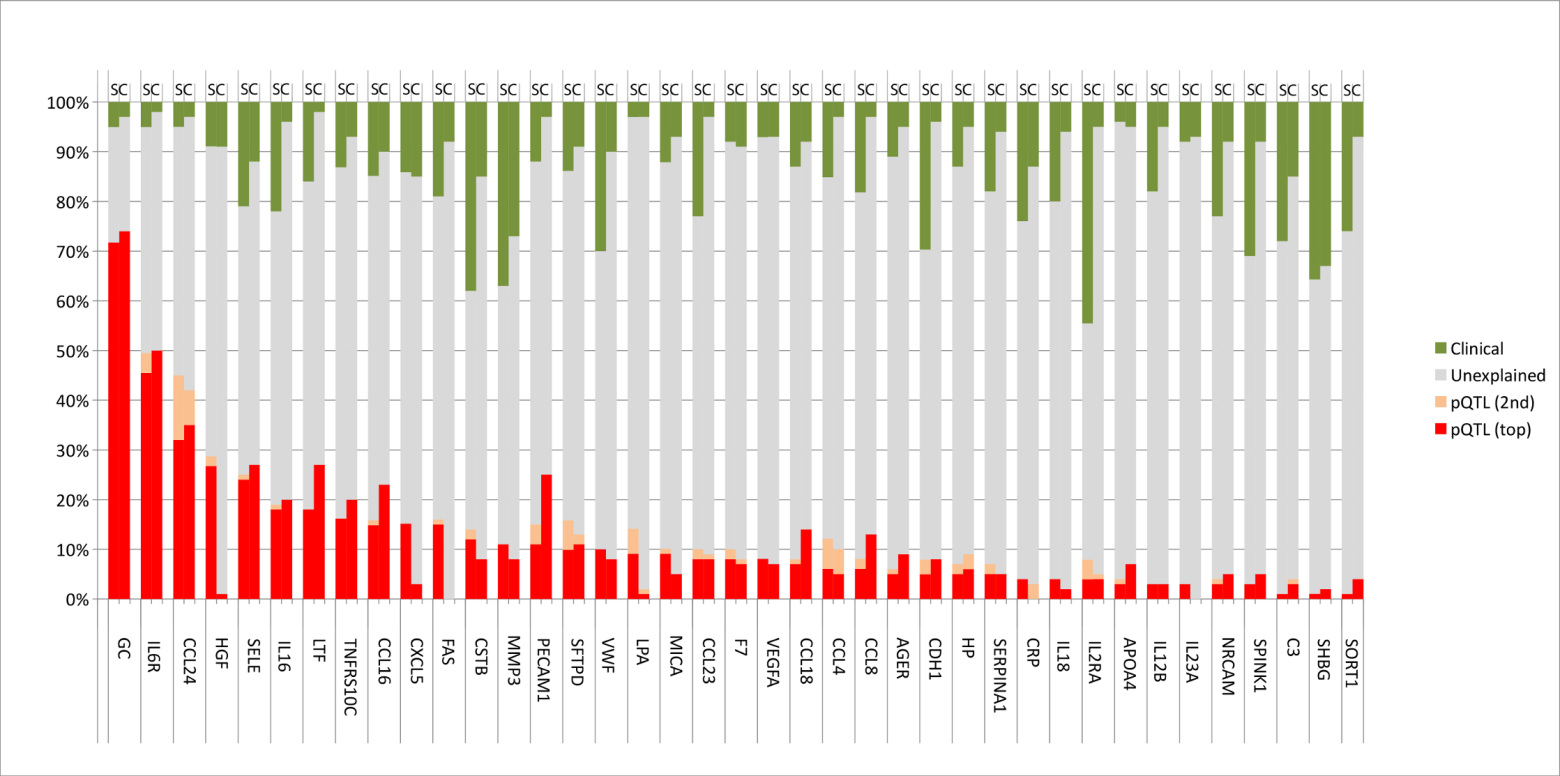
Blood biomarker variance explained by top two pQTLs SNPs and clinical covariates. The percent variation for 39 blood biomarkers explained by clinical (green) top pQTL SNP (red), second top independent pQTL SNP (peach), other unknown factors (grey). Clinical factors include age, gender, body mass index, smoking status, and principal components of ancestral genetic markers as described in the methods. The analysis includes subjects from SPIROMICS (S) and COPDGene (C) cohorts. TNRF (TNF-Related Apoptosis-Inducing Ligand Receptor 3 (TRAIL-R3)); PCAM (Platelet endothelial cell adhesion molecule (PECAM-1)); SRP1 (Alpha-1-Antitrypsin (alpha-1 (AAT)); NRC (Neuronal Cell Adhesion Molecule (Nr-CAM)); SPK (Pancreatic secretory trypsin inhibitor (TATI)); SRT1 (Sortilin); other abbreviations are listed in Table 1 and S2 Table.

**Fig 6:**
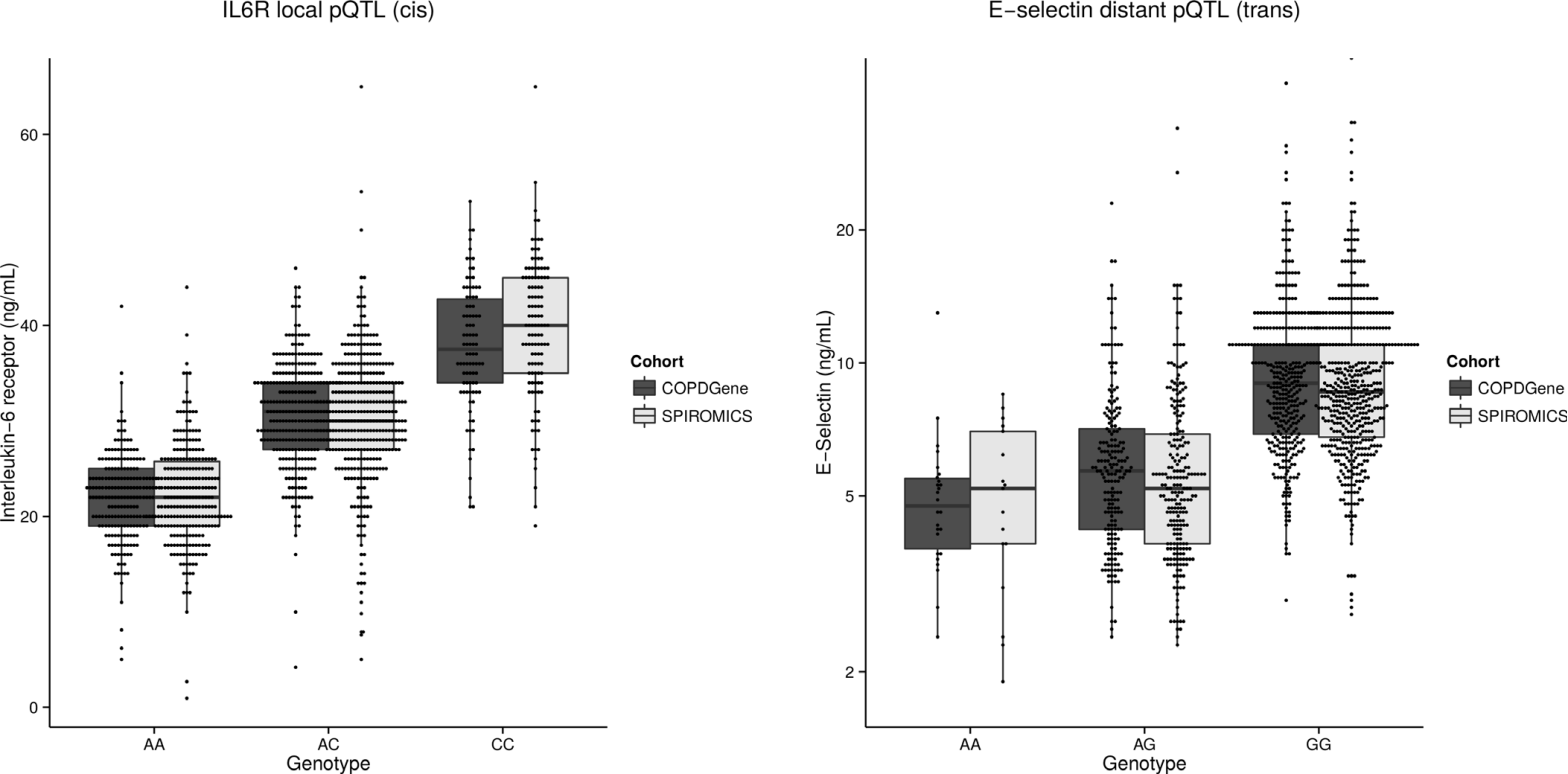
Examples of biomarker pQTL SNPs. Plasma levels of IL6R (*A*) and E-selectin (*B*) are strongly influenced by pQTL SNPs (P = 10^−193^ and P = 4 X 10^−104^). The pQTL SNP for IL6R is on chromosome 4, which is local (*cis*) to *IL6R*, the gene coding for its protein. The pQTL SNP for E-selectin protein is on chromosome 9, which is distant (trans) from *SELE* (chromosome 1), the gene coding for its protein. This pQTL SNP is in the *ABO* locus, which encodes alpha 1-3-N-acetylgalactosaminyltransferase.

Nine biomarkers had at least 10% of their variance explained by a single pQTL SNP in both SPIROMICS and COPDGene (Fig 5). For example, a single local pQTL SNP (rs8192284 SNP in *IL6R*) explained 45% of variance of plasma IL6R in SPIROMICS and 50% of this variance in COPDGene, and a single distant pQTL SNP (rs507666 SNP in *ABO*) explained 25% of variance of blood E-selectin (*SELE*) in SPIROMICS and 27% of variance in COPDGene (Fig 6). In many cases, pQTL SNPs explained more variance in the quantitative biomarker than did clinical covariates.

To assess the novelty of these pQTL SNPs, we cross-referenced the unique 478 pQTL SNPs we identified with SNPs associated with any published GWAS based on NHGRI GWAS catalog, including those related to COPD phenotypes or pulmonary function (n = 242). By these criteria, 90% of pQTL SNPs were novel (P < 10^−34^; S4 Table), even after removing SNPs in linkage disequilibrium [280 significant pQTL SNPs remained and, of those, 29 (10.4%) overlapped with at least one GWAS report (P < 10^−20^)].

**Fig 7:**
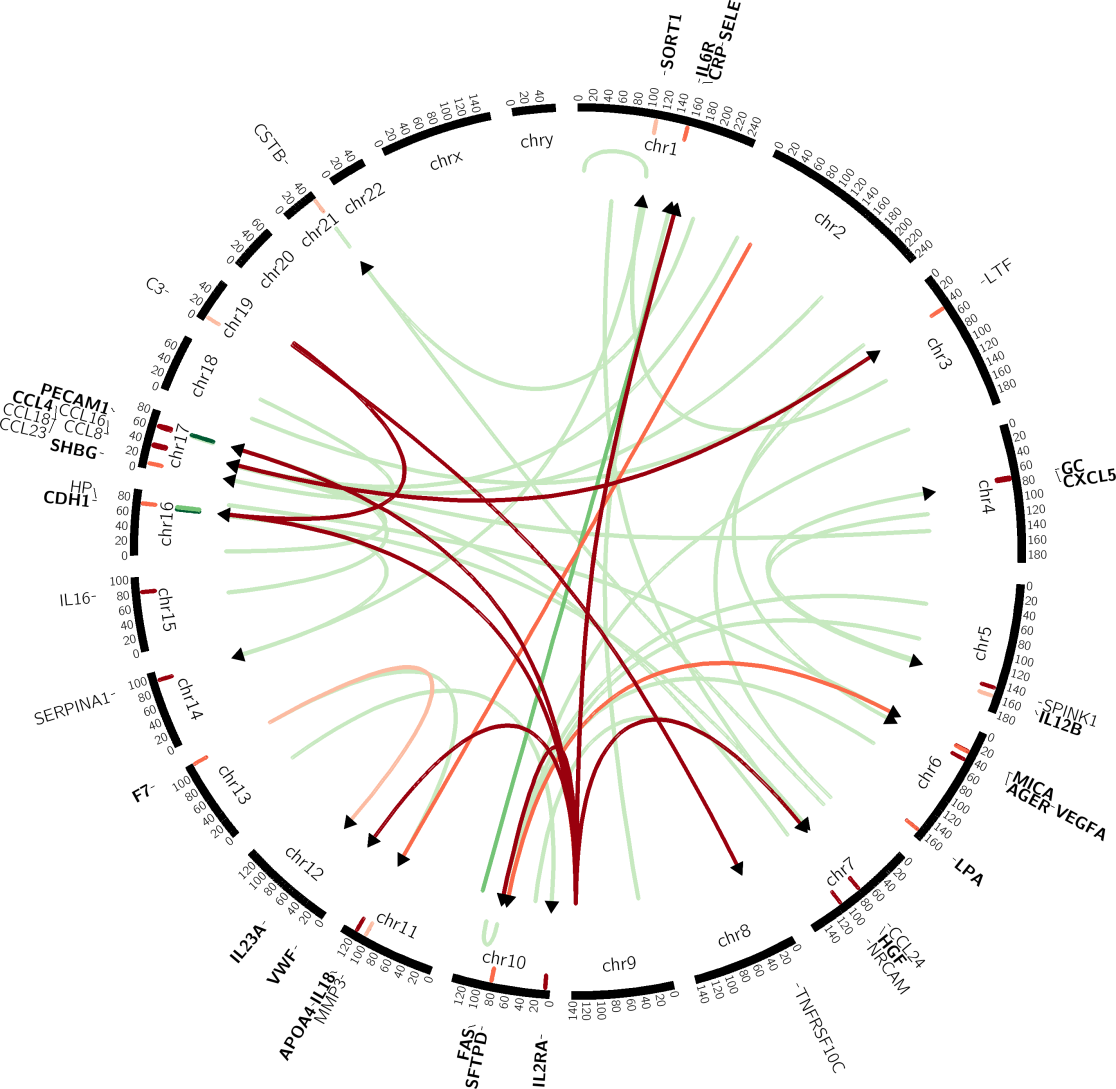
Circos plot showing distant (*trans*) pQTLs and their relationships to eQTL SNPs. The arrows in the inner circle represent pQTL SNPs significantly associated (beginning of arrow) with biomarker (end of arrow). Biomarker abbreviations (see text for full list) are shown on the outer ring. Local (*cis*) pQTL SNPs are shown as hash marks adjacent to biomarker gene location. The color of represents significance of association. Red lines are associations between genes. The thinnest, darkest red lines signify associations with significance of P = 10^−12^, and the lines become slightly thicker and darker at the significance levels of P = 10^−10^ and P = 10^−8^. The green lines signify eQTL associations. The cutoffs for line thickness and darkness for the green lines are P = 10^−7^ and P = 10^−6^. The only eQTL association with significance P < 10^−8^ were local, near the HP and PECAM1 genes.

We next evaluated whether pQTL SNPs were also eQTLs, by utilizing an overlapping dataset of peripheral blood mononuclear cell gene expression from COPDGene [32]. In this analysis, only COPDGene data were available, so results are limited to this dataset. Although there were more positive correlations between gene expression and protein levels than expected by chance (sign test P = 0.0009), the overall magnitudes of such correlations were low (S8 Fig), and there was little overlap between pQTL and eQTL SNPs (Fig 7; S6 Table). Furthermore, as previously shown, although both eQTL and pQTL SNPs were more likely to be intronic [20], among those that were not, pQTL SNPs were more likely to be in 5′ or 3′ untranslated region or to be missense SNPs, compared to eQTL SNPs (S9 Fig). Only one biomarker (haptoglobin, corresponding to gene HP) had pQTL SNPs that were also eQTL SNPs, and this is the only case where regression modeling suggested that measured biomarker levels are mediated by gene expression (S6 Table).

Given that QTLs may be dependent upon the cellular/tissue-specific expression [74], we examined whether the pQTLs would be significantly affected by the cellular composition of the blood by repeating the pQTL analysis adding cell counts (red blood cells, neutrophils, lymphocytes, basophils, monocytes, eosinophils, and platelets) as covariates in the models. For either all possible SNPs or only significant pQTL SNPs, the correlation between the p-values of the pQTLs with and without blood cell counts added as covariates was > 0.985, indicating that the pQTLs were not markedly dependent upon blood cell type composition (S10 Fig).

A recent report suggests that monoclonal antibodies for vitamin D binding protein may preferentially recognize a selected protein isoform [75] caused by the rs7041 pQTL, which is a missense mutation causing aspartic acid to glutamic acid change at position 432 (D432E). Therefore we used a polyclonal antibody to compare to measurements to the monoclonal assay used on the RBM platform in a subset of SPIROMICS subjects. Indeed, the measurements using the monoclonal antibody were significantly lower for the TT genotype compared to the GG genotype (P < 0.001), suggesting that measurements using the monoclonal antibody assay detected the D432E protein isoform less well compared to the polyclonal assay (S11 Fig).

### The relationship between pQTL SNPs and COPD disease phenotypes

**Fig 8:**
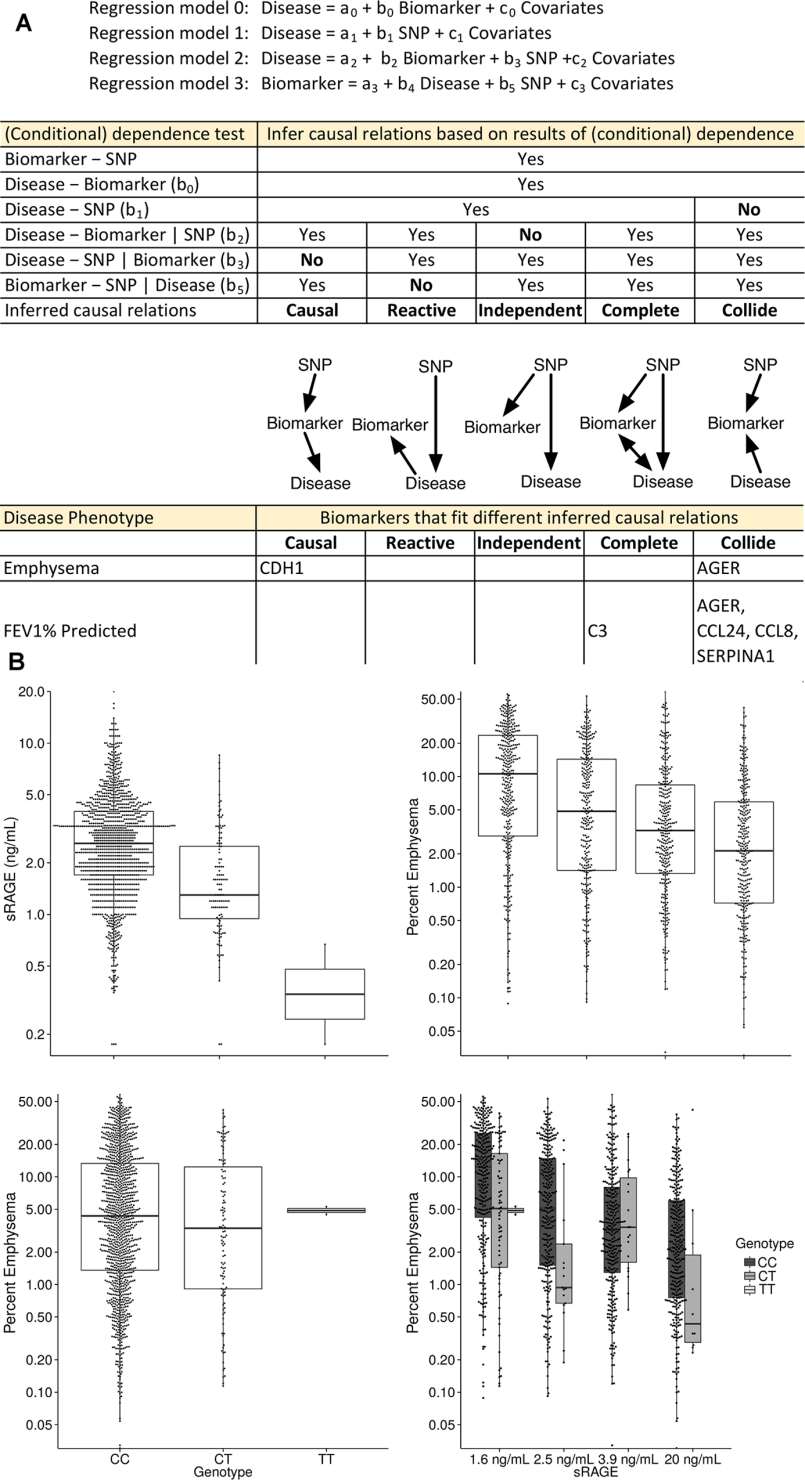
Clinical and biologic significance of pQTL SNPs. (A) Biomarker pQTL SNPs were tested for association with four different COPD disease phenotypes: emphysema, airflow limitation (FEV_1_%), chronic bronchitis, and exacerbations using four different statistical regression models to infer the causal relations of causal, reactive, independent, complete or collide. A complete listing of pQTL SNPs disease association p-values for both cohorts can be found in S8 Table. Note that testing b_2_ = 0 and b_4_ = 0 are equivalent because in both cases, we are testing whether the disease and biomarker is conditionally dependent given SNP. Therefore, we only examined b2 in our analysis. No significant results were obtained for chronic bronchitis or exacerbations and so these two phenotypes are not shown. (B) The T allele of rs2070600 is associated with lower plasma levels of sRAGE and (C) lower plasma levels of sRAGE (shown by sRAGE quartile) are associated with more emphysema on quantitative CT scan (model 0); (D) the T allele is not clearly associated with emphysema when considering only the SNP-disease association (model 1); however, (E) the T allele is associated with less emphysema within each biomarker quartile (model 2), and the SNP fits the collide model.

With SNPs, biomarker levels, and disease phenotypes all available for both cohorts, statistical modeling could be used to infer the relationships among these three data types employing methods previously applied to eQTL-gene expression-phenotype relationships [22–27]. We chose four clinically important COPD phenotypes [airflow obstruction (FEV_1_% predicted), emphysema, chronic bronchitis, and a history of exacerbations] and applied regression models adjusted for covariates and PCs [22, 26]. We categorized the relationships of all 2,108 trios of SNP, biomarker, and disease phenotype (527 pQTL SNP/biomarker pairs and four disease phenotypes) into five categories, based on (conditional) dependence testing (Fig 8 and full results supporting Fig 8, including regression coefficients, are in S7 Table). Results for biomarker associations to disease phenotype for pQTL SNPs are also provided (S8 Table).

Significant evidence for inferred causal, complete, or collide relationships were found for emphysema and airflow obstruction for six biomarkers, with AGER represented by the same model in both phenotypes (Fig 8). In all of these cases, the direction of the regression coefficients were the same between SPIROMICS and COPDGene (S7 Table). By contrast, no significant relationships were found for chronic bronchitis or exacerbations. In the case of the collide model, the association between pQTL SNP and disease phenotype is strengthened given the biomarker, and thus inclusion of pQTL SNP information in biomarker-disease association testing will add predictive value. An example is AGER, which is classified as the “collide” model for the phenotype of emphysema. Including both AGER levels and its top pQTL SNP improved the explanation of variance (R^2^) for emphysema to 40%, compared to just 30% for the biomarker alone, and 22% when only clinical covariates were used.

## Discussion

In this study we identified hundreds of novel SNPs significantly associated with nearly 40% of blood biomarkers commonly used in both pulmonary and non-pulmonary clinical research. For many biomarkers, a single pQTL SNP accounted for a large percentage of measured variance. We demonstrated that pQTLs provide unique information compared to eQTLs and that inclusion of pQTL SNPs can improve explanation of variance when added to clinical covariates in statistical models, *e*.*g*., sRAGE and emphysema. Although the subjects in this study were recruited for COPD phenotypes, many of the pQTLs identified and the biomarkers studied have been associated with other diseases or traits, suggesting that the pQTL-biomarker relationships reported here are broadly relevant to human pathophysiology. Furthermore, the pQTL-biomarker-disease phenotype relationship is frequently not a simple SNP → gene expression → biomarker → disease phenotype association. These findings suggest that modeling with inclusion of measurements from multiple omics technologies may be needed to optimize precision medicine predictions.

A significant finding in this study is the number of distant pQTLs associated with the *ABO* locus (commonly associated with ABO blood group). PQTLs at the *ABO* locus were the strongest genetic association among six proteins encoded by genes on six different chromosomes. This *ABO* region, along with the *FUT2* gene (galactoside 2-alpha-L-fucosyltransferase 2), which contained pQTLs for CDH1, was found to overlap with a growing number of previously reported QTLs for a variety of blood analytes, blood processes (such as clotting time), metabolites, lipids, and even urinary metabolites (Fig 3). The most likely explanation is these two loci affect enzymes that post-translationally modify multiple proteins leading to impaired protein function, half-life, or detection. Interestingly, older literature, prior to extensive genotyping and biomarker analysis, has reported association between ABO blood group and COPD [76] and has been associated with other diseases such as goiter [77] and hepatitis [78] in the candidate gene era. The extensive number of associations now reported at the ABO blood group from a wide variety of studies suggests that greater attention should be paid to ABO status for blood biomarker studies.

Much of the recent effort to identify genetic variants and genomic signatures associated with clinical disease has extensively used eQTLs to understand the function of loci identified in GWAS, including for COPD [4, 79–81]. We demonstrate a clear distinction between known eQTLs and pQTLs, which is consistent with previous work that compared variants associated with three different levels of gene regulation (transcription, translation and protein levels) in a study of 62 HapMap Yoruba (Ibadan, Nigeria) lymphoblastoid cell lines (LCLs) [5]. The authors used SILAC mass spectrometry to quantitate proteins and showed that only 35% of the pQTL variants overlapped with eQTLs using RNAseq. Some of the variance in protein expression was due to ribosomal occupation (ribosomal profiling); however, there were many pQTLs in which there was little variation in the mRNA or ribosomal profiling, suggesting that post-translational events may be responsible for differences in protein abundance. Similar to what we report, this is supported by the observation that the pQTLs are significantly enriched in protein coding (missense) and potential translational regulation *(e*.*g*., 3’ UTR) regions. They hypothesize this may be due to differences in protein degradation; however one cannot exclude that the peptide variants may be differentially measured with mass spectrometry, or that there may be altered biomarker stability, secretion rates, or processing/release from the cell surface. Another limitation of this study is that they only considered genetic variants within a 20-kb window around the corresponding gene; however, we found a significant number of pQTL SNPs mapped outside of this region. Another study of 441 transcription factors and signaling proteins in the Yoruban LCLs found that many pQTLs were not associated with gene expression and were also distant from the corresponding gene [82]. These studies highlight the general need to include protein expression in large-scale population variation studies such as GTEx to better understand the relationship between genome and protein in humans. Although such efforts are ongoing on a small scale (*e*.*g*. Chromosome-Centric Human Proteome Project [83]), our results imply these efforts can also be incorporated cost-effectively into large existing clinical cohorts.

These findings will be useful for GWAS and biomarker studies of other diseases. For instance, we identified novel pQTL SNPs explaining greater than 25% of variance in blood proteins such as interleukin 6 receptor, eotaxin-2, and E-selectin, which could be useful in studies of asthma and of non-pulmonary diseases. The sRAGE-emphysema example demonstrates that the application of causal modeling can provide new insights to the relationship between SNP, measured biomarker levels, and disease phenotypes. Additionally, this example demonstrates how predictive models of disease phenotype can be improved by adding pQTL information.

Furthermore, evaluating all possible statistical relationships among pQTL SNPs, biomarkers, and disease phenotypes suggests that many pQTL SNP effects may not be causally mediated directly through measured biomarkers. For instance, the minor allele rs2070600 SNP in *AGER* is associated with lower sRAGE in blood; COPD severity and emphysema extent have also been negatively associated with lower blood sRAGE concentrations in cross-sectional studies [13, 14]. Paradoxically, however, in large GWAS studies, the minor allele of rs2070600 is associated with reduced COPD severity and reduced emphysema [80, 81] suggesting potentially opposite effects of the SNP. Indeed, our evidence points to a “collide” relationship; however, given the previous published large scale genetic association studies have shown that rs2070600 is associated with COPD and emphysema, it is likely that this study is underpowered to distinguish between the “collide” and the “complete” model, which can be distinguished by a statistically significant association between the pQTL SNP and disease phenotype. Nevertheless, the association between pQTL SNP and disease phenotype becomes much stronger given the biomarker, which implies the collide relation. Regardless of whether rs2070600 is “collide” or “complete”, it is a missense SNP that causes a G82S amino acid change and thus illustrates the enrichment of coding SNPs in pQTL analysis. The mechanism by which rs2070600 causes disease is unknown, but the resultant amino acid substitution may block shedding of this cell surface receptor, reducing blood levels but at the same time improving sensing of damage-associated molecular pattern molecules, with a net protective effect [84]. However, once emphysema progresses, the source of sRAGE in the blood (the alveolar cells) is reduced, so that emphysema progression would be manifested by reduced sRAGE levels.

Several other relationships identified are also worth considering. For example, we identified evidence for the “collide” relationship for rs926144, an intergenic SNP in SERPINA1 (alpha-1-antitrypsin; AAT), a protein whose normal function is linked directly to the development of emphysema. Although we find strong pQTL SNPs for SERPINA1, and we see a relationship between COPD and SERPINA1 levels, we see no statistically significant evidence that pQTL SNPs associate directly with disease. This is similar to what authors of an GWAS of AAT serum levels have recently reported in this journal [85], in which they identified strong serum AAT pQTLs, but their association with lung function was driven by the rare disease variants (PiSZ and pZZ, who were excluded from SPIROMICS and COPDGene). Since SERPINA1 is produced by the liver and is well-known as marker of systemic inflammation, an established feature of COPD, this would support the finding that common SNPs may not be representative of the known disease-causing variants, which are rare, and that both non-disease causing variants and the disease itself may be associated with changes in biomarker levels.

We found that a “complete” model was suggested for the Complement Factor 3 (C3) pQTL SNP rs2230203. In a study of 111 subjects with COPD and 111 matched controls, blood C3 was noted to be lower in COPD subjects [86]. Similarly in a more recent study of 15 COPD subjects and 15 matched controls serum C3 was lower in COPD subjects [87]. Our findings confirm the relationship between C3 and COPD and emphysema and further suggest that it is partly mediated through C3 genetic variants. Although the rs2230203 variant is in the coding region of *C3*, it is a synonymous variant and was the only pQTL we identified for C3. The variant might affect protein levels though siRNA binding or other pre-translational mechanisms, but mechanistic studies will be necessary to confirm this.

As a final example, the “causal” relationship suggested for CDH1 (E-cadherin) for both emphysema and FEV_1_% predicted is also intriguing at a mechanistic level. The CDH1 pQTL SNPs are distant (*trans*) and are located in *FUT2*, which codes for a fucosyltransferase that, along with *ABO*, determines the expression of distinct blood group antigens. Evidence for a role of CDH1 and COPD is growing [13, 88, 89], yet the underlying mechanisms are not entirely clear. Our results suggest that future studies should focus on a direct role of CDH1 in the pathogenesis of disease.

Strengths of this study include the large number of subjects and the inclusion of validation cohorts. However, there are some limitations. Although it is one of the largest biomarker-GWAS studies reported, 1,340 subjects is still small compared to clinical GWAS studies, thus we are likely underpowered to detect some of the SNP-disease phenotype associations. Thus, we cannot say for certain, for example, that a causal or collide model might not actually be a complete model (e.g. for rs207060 in *AGER* with sRAGE). Second, because we identified distinct and independent pQTL SNPs for some biomarkers, there may be multiple mechanisms by which pQTL biomarkers mediate SNP-biomarker-disease phenotype interactions. Proving the validity of the causal inference models will require detailed mechanistic studies at both a genomic and proteomic level. Additionally, like nearly all biomarker assays, we used antibody based detection methods to measure biomarkers. Since antibodies recognize specific epitopes on proteins, it is possible that our pQTL may detect a specific isoforms of a protein rather than total protein. This has recently been suggested, but not proven, as an explanation for the strong genetic (racial) associations observed for vitamin D binding protein and the *cis*-SNP rs7041 (Asp432Glu). As we have and others have shown for vitamin D binding protein [75], assays that use polyclonal antibodies compared to the monoclonal sandwich immunoassay (R&D Systems) may overcome this limitation. Another example in the literature is a pQTL identified for TNF-alpha was not replicated when a different assay was applied to the same samples [10]. However, similar pQTLs for plasma proteins such as HP, SERPINA1, C3, APOE, and AHSG were identified using mass spectrometry [90] and for IL6R, F7, and others using aptamer-based detection [91], suggesting many pQTLs we identified were not platform specific. Thus, knowing that antibody used in biomarker measurement may preferentially detect a specific isoform of a protein does not discount its importance, particularly if the pQTL SNP has also been associated with the disease phenotype in genetic association studies, as is the case with vitamin D binding protein, sRAGE, and several other pQTL SNPs described in this study (see Table 2). Thus, investigators who conduct biomarker studies need to consider the possibility that genotype plays a role when measuring blood biomarkers.

An additional limitation of the study is using a candidate panel of 114 biomarkers that are overrepresented for inflammation and lung proteins. At the time, this was state of the art for large scale human studies; however, in the future there will be high-throughput, 1000+ biomarker panels that may be used such as SomaScan (Somalogic, Boulder, Colorado). Other limitations of this study include that it was limited to subjects over 45 years of age and only NHW subjects. Future studies should include other populations and the types of variants assessed, *e*.*g*., rare variants. Finally, due to the nature of the available data, evaluating quantitative change in biomarkers with disease progression was not conducted, but would be expected to enhance understanding of disease mechanisms in future studies.

In summary, this large scale, dual-cohort, combined GWAS and biomarker study represents a powerful approach to combine different omics data sets to better understand complex diseases such as COPD. We replicated some previously reported pQTL associations and discovered a large number of novel pQTLs, including distant pQTLs, which many studies are poorly powered to detect. Integration of pQTL genotypes with biomarker measurements will improve the precision of disease prediction for some clinically relevant phenotypes, and improve the mechanistic understanding of others, thus increasing the implementation of targeted clinical care.

## Supporting Information

S1 Table Analyte measurement details for SPIROMICS and COPDGene. LLOQ = Lower Limit of Quantification. All COPDGene samples were P100 plasma. Green cells under “BIOMARKER Variable Name” represent analytes that were evaluated in this work (those not analyzed had a high % below LLOQ).
(DOCX)

S2 Table Demographic features of cohort in current manuscript compared to comparable non-Hispanic White cohort data from the larger study cohorts. (DOCX)

S3 Table SNPs associated with biomarker levels in each cohort at P < 10^−5^ and designation of those that replicate by both significance and direction. (XLS)

S4 Table All significant meta-analysis pQTLs, their minor allele frequencies (MAF), designation of uniqueness, and predicted functional consequences. The table is sorted alphabetically by gene name and then sorted by "weighted meta-analysis P-value". Distant pQTLs are denoted by light tan shading. pQTLs determined by the tobit model are designated by an * next to the gene name. The NHGRI GWAS catalog was searched 5-8-2015; pQTLs are unique if they are not listed in the GWAS catalog (GWAS) or not in LD with any SNP in the GWAS catalog (LD-GWAS). Traits listed for GWAS catalog SNPs and PMID numbers for the appropriate references are provided. Functional annotation of SNPs (variant effect predictor): up (upstream), 5′ (5′ untranslated region), syn (synonymous variant), mis (missense), int (intron), stop (stop gained), splice (splice region variant), 3′ (3′ untranslated region), down (downstream), inter (intergenetic), nc (non-coding transcript exon). The SNPs that do not replicate in direction of effect between the two cohorts are indicated in red.
(XLSX)

S5 Table pQTL SNPs that show independent evidence for association with blood analyte levels as compared to the top reported eQTL SNP. The method utilized to determine these pQTLS is described in detail in the Methods (recursive conditioning). Results for both COPDGene and SPIROMICS are shown. None = all pQTLs are in strong linkage disequilibrium with the top SNP.
(DOCX)

S6 Table Significant expression eQTLs for the blood biomarkers tested in this study (as described in methods). Only those for HP (red) were also pQTLs (S4 Table). CDH1 and PECAM eQTLs are local, while pQTLs for these two analytes were distant. RNA expression levels of PECAM1 are measured by two different ProbeSetIDs in the Affymetrix arrays used for the gene expression studies. For HP, the model that best fits the evidence is listed. Causal in this case indicates that the evidence supports gene expression levels producing altered protein levels. Modeling for HP was conducted as for Fig 8 in the main manuscript using HP levels in place of disease.
(DOCX)

S7 Table Significant results of pQTL-biomarker-disease association testing. Specifics of the analysis are described in Fig 8 of the main text and in the methods section. Results are shown separately for the two COPD phenotypes with significant associations (percent emphysema and FEV1 percent predicted (FEVpp)]. While all pQTL SNPs were tested (results for biomarker associations shown in S8 Table), only those showing evidence for the models causal, reactive, independent, complete or collide are indicated in this table.
(XLSX)

S8 Table Complete of pQTL-biomarker-disease association testing results as indicated for S7 Table. (XLS)

S1 Fig Examples of assay validation. A) Comparison of selected biomarker values on two different platforms by Quotient Bio Research (QBR) and Myriad Rules Based Medicine (RBM) from a selected subset of COPDGene subjects. The correlation coefficients are shown in the upper right panel and the scatterplots in the lower left panel. A histogram of the biomarker values are shown on the diagonal plots. B) Comparison of an R&D Quantikine ELISA (X axis) from serum of selected SPIROMICS subjects to the RBM (Y axis) for vitamin D binding protein. The two assays are highly concordant. See methods for details.
(DOCX)

S2 Fig Barplot of eigen-values of PCA analysis in SPIROMICS biomarker data. We ran PCA on biomarker data after regressing out all the other covariates used in pQTL analyses. Based on the sizes of eigen-values shown in this plot, we choose to include the first PC into our pQTL analysis to account for unobserved confounding effects.
(DOCX)

S3 Fig Histograms demonstrating phenotype frequencies. SPIROMICS (top) and COPDGene (bottom) for (A) chronic bronchitis (0 = no; 1 = yes), (B) frequency of exacerbations in the 12 months prior to enrollment (exacerbations include respiratory events that required doctor visit, emergency room visit, hospitalization, or a change in antibiotic or steroid use), (C) FEV_1_% predicted, (D) percent total lung emphysema as defined by Hounsfield units -950; and (E) log transformation of percent emphysema.
(DOCX)

S4 Fig Manhattan plots, q-q plots, and LocusZoom plots of pQTL findings for all analytes where significant pQTLs were identified. LocusZoom plots show a 90 kb window (center) or a 500 kb window (right). On the Manhattan plots, the red line is the significant threshold after correction for multiple comparisons. For all LocusZoom plots, the top pQTL SNP is indicated. Red boxes in the LocusZoom plots show the location of the analyte gene (not all plots show this location but local pQTLs will have a box in one or both plots; for distant pQTLs, no red box will be present in either plot). Analyte gene location is shown with red arrow in Manhattan plots.
(PDF)

S5 Fig Summary matrix of pQTLs. Each dot represents a pQTL with P < 10^−8^. The x-axis denotes the location of the pQTL SNP and the y-axis denotes the location of the biomarker. The color of each dot denotes range of P-value as indicated in the legend. Dots more than 1 Mb from the identity line represent distant pQTL SNPs. Dots on the identity line represent local pQTLs. The bottom panel is useful to highlight the peak of pQTL SNPs located on chromosome 9 (*ABO* locus).
(DOCX)

S6 Fig **The highly significant pQTL SNPs (right panels) represent a distribution of minor allele frequencies similar in distribution to all SNPs in the study (left panels).** SPIROMICS (top panel); COPDGene (bottom panel).
(DOCX)

S7 Fig Percent variance explained within and between studies. a)-b) For both cohorts, the percent variance explained (R2) was greater in the full model, which includes all covariates in addition to the top two independent SNP genotypes, compared to the genotype only model. The correlation (rho) between the two models was higher for COPDGene (0.92) compared to SPIROMICS (0.72). This indicates that utilized covariates are relatively more predictive of biomarker levels in SPIROMICS compared to COPDGene. c)-d) Percent variance explained correlated between COPDGene and SPIROMICS, with only genotype producing a stronger correlation (rho 0.88) compared to the full model (rho = 0.72). Thus, genotype in both cohorts have similar contributions to the percent variation in biomarker levels, while the contribution by the covariates is more variable and study dependent.
(DOCX)

S8 Fig Correlation between gene expression and biomarker level. For 103 subjects, both gene expression and biomarker data were available from the COPDGene cohort. In those subjects, 80 biomarkers had available gene expression data in 199 probesets (multiple probesets may be available for a gene). For these 199 biomarker-gene expression pairs, there is a significant number of positive correlations (0.007, sign test) indicating that eQTLs (based on mRNA) can effect blood biomarker levels.
(DOCX)

S9 Fig VEP analysis to evaluate the characteristics of the pQTL SNPs in comparison to eQTLs from various sources and the published findings from the NHGRI Catalog. This figure represents an expanded version of Fig 3 in the main text.
(DOCX)

S10 Fig Significant pQTLs are not affected by total blood cell counts (CBC). When cell counts (eosinophils, basophils, neutrophils, monocytes, platelets, and red blood cells) were included in the regression models (Tobit or Linear as described in the methods) the significance of the pQTLs (X axis) did not vary significantly from results that did not include the CBC data (Y-axis). This was true for all pQTLs (left) and for the significant pQTLs (right). CBC data was only available from SPIROMICS and so these graphs represent SPIROMICS-only p-values. The correlation = 0.9854 for all SNP biomarker pairs and >0.999 for significant pQTL biomarker pairs.
(DOCX)

S11 Fig Comparison of vitamin D binding protein levels using a monoclonal antibody assay versus polyclonal assay for selected SPIROMICS subjects with GG or TT genotypes at rs7041. (DOCX)

S1 File IRB approvals for both studies. (DOCX)

## References

[1] Locke AE, Kahali B, Berndt SI, Justice AE, Pers TH, Day FR, et al Genetic studies of body mass index yield new insights for obesity biology. Nature. 2015;518(7538):197–206. 10.1038/nature14177 25673413 PMC4382211

[2] Wood AR, Esko T, Yang J, Vedantam S, Pers TH, Gustafsson S, et al Defining the role of common variation in the genomic and biological architecture of adult human height. Nat Genet. 2014;46(11):1173–86. 10.1038/ng.3097 25282103 PMC4250049

[3] Consortium GT. Human genomics. The Genotype-Tissue Expression (GTEx) pilot analysis: multitissue gene regulation in humans. Science. 2015;348(6235):648–60. 10.1126/science.1262110 . 25954001 PMC4547484

[4] Westra HJ, Franke L. From genome to function by studying eQTLs. Biochim Biophys Acta. 2014;1842(10):1896–902. 10.1016/j.bbadis.2014.04.024 . 24798236

[5] Battle A, Khan Z, Wang SH, Mitrano A, Ford MJ, Pritchard JK, et al Genomic variation. Impact of regulatory variation from RNA to protein. Science. 2015;347(6222):664–7. 10.1126/science.1260793 . 25657249 PMC4507520

[6] Couper D, LaVange LM, Han M, Barr RG, Bleecker E, Hoffman EA, et al Design of the Subpopulations and Intermediate Outcomes in COPD Study (SPIROMICS). Thorax. 2014;69(5):491–4. 10.1136/thoraxjnl-2013-203897 24029743 PMC3954445

[7] Regan EA, Hokanson JE, Murphy JR, Make B, Lynch DA, Beaty TH, et al Genetic epidemiology of COPD (COPDGene) study design. COPD. 2010;7(1):32–43. 10.3109/15412550903499522 20214461 PMC2924193

[8] Burney PG, Patel J, Newson R, Minelli C, Naghavi M. Global and regional trends in COPD mortality, 1990–2010. Eur Respir J. 2015;45(5):1239–47. 10.1183/09031936.00142414 . 25837037 PMC4531307

[9] Faner R, Tal-Singer R, Riley JH, Celli B, Vestbo J, MacNee W, et al Lessons from ECLIPSE: a review of COPD biomarkers. Thorax. 2014;69(7):666–72. 10.1136/thoraxjnl-2013-204778 . 24310110

[10] Melzer D, Perry JR, Hernandez D, Corsi AM, Stevens K, Rafferty I, et al A genome-wide association study identifies protein quantitative trait loci (pQTLs). PLoS Genet. 2008;4(5):e1000072 10.1371/journal.pgen.1000072 18464913 PMC2362067

[11] Bowler RP, Kim V, Regan E, Williams AA, Santorico SA, Make BJ, et al Prediction of acute respiratory disease in current and former smokers with and without COPD. Chest. 2014;146(4):941–50. 10.1378/chest.13-2946 24945159 PMC4188150

[12] O'Neal WK, Anderson W, Basta PV, Carretta EE, Doerschuk CM, Barr RG, et al Comparison of serum, EDTA plasma and P100 plasma for luminex-based biomarker multiplex assays in patients with chronic obstructive pulmonary disease in the SPIROMICS study. J Transl Med. 2014;12:9 10.1186/1479-5876-12-9 24397870 PMC3928911

[13] Carolan BJ, Hughes G, Morrow J, Hersh CP, O'Neal WK, Rennard S, et al The association of plasma biomarkers with computed tomography-assessed emphysema phenotypes. Respir Res. 2014;15:127 10.1186/s12931-014-0127-9 25306249 PMC4198701

[14] Cheng DT, Kim DK, Cockayne DA, Belousov A, Bitter H, Cho MH, et al Systemic soluble receptor for advanced glycation endproducts is a biomarker of emphysema and associated with AGER genetic variants in patients with chronic obstructive pulmonary disease. Am J Respir Crit Care Med. 2013;188(8):948–57. 10.1164/rccm.201302-0247OC . 23947473

[15] Agusti A, Edwards LD, Rennard SI, MacNee W, Tal-Singer R, Miller BE, et al Persistent systemic inflammation is associated with poor clinical outcomes in COPD: a novel phenotype. PLoS One. 2012;7(5):e37483 10.1371/journal.pone.0037483 22624038 PMC3356313

[16] Lomas DA, Silverman EK, Edwards LD, Locantore NW, Miller BE, Horstman DH, et al Serum surfactant protein D is steroid sensitive and associated with exacerbations of COPD. Eur Respir J. 2009;34(1):95–102. 10.1183/09031936.00156508 . 19164344

[17] Purcell S, Neale B, Todd-Brown K, Thomas L, Ferreira MA, Bender D, et al PLINK: a tool set for whole-genome association and population-based linkage analyses. Am J Hum Genet. 2007;81(3):559–75. 10.1086/519795 17701901 PMC1950838

[18] Cho MH, McDonald ML, Zhou X, Mattheisen M, Castaldi PJ, Hersh CP, et al Risk loci for chronic obstructive pulmonary disease: a genome-wide association study and meta-analysis. Lancet Respir Med. 2014;2(3):214–25. 10.1016/S2213-2600(14)70002-5 24621683 PMC4176924

[19] Greene WH. Econometric analysis 6th ed. Upper Saddle River, N.J.: Prentice Hall; 2008 xxxvii, 1177 p. p.

[20] Wright FA, Sullivan PF, Brooks AI, Zou F, Sun W, Xia K, et al Heritability and genomics of gene expression in peripheral blood. Nat Genet. 2014;46(5):430–7. 10.1038/ng.2951 24728292 PMC4012342

[21] Richardson DB, Ciampi A. Effects of exposure measurement error when an exposure variable is constrained by a lower limit. Am J Epidemiol. 2003;157(4):355–63. . 12578806 10.1093/aje/kwf217

[22] Schadt EE, Lamb J, Yang X, Zhu J, Edwards S, Guhathakurta D, et al An integrative genomics approach to infer causal associations between gene expression and disease. Nat Genet. 2005;37(7):710–7. 10.1038/ng1589 15965475 PMC2841396

[23] Chen Y, Zhu J, Lum PY, Yang X, Pinto S, MacNeil DJ, et al Variations in DNA elucidate molecular networks that cause disease. Nature. 2008;452(7186):429–35. 10.1038/nature06757 18344982 PMC2841398

[24] Millstein J, Zhang B, Zhu J, Schadt EE. Disentangling molecular relationships with a causal inference test. BMC Genet. 2009;10:23 10.1186/1471-2156-10-23 19473544 PMC3224661

[25] Li Y, Tesson BM, Churchill GA, Jansen RC. Critical reasoning on causal inference in genome-wide linkage and association studies. Trends Genet. 2010;26(12):493–8. 10.1016/j.tig.2010.09.002 20951462 PMC2991400

[26] LaMontagne AD, Milner A, Krnjacki L, Kavanagh AM, Blakely TA, Bentley R. Employment arrangements and mental health in a cohort of working Australians: are transitions from permanent to temporary employment associated with changes in mental health? Am J Epidemiol. 2014;179(12):1467–76. 10.1093/aje/kwu093 . 24872351

[27] Chen LS, Emmert-Streib F, Storey JD. Harnessing naturally randomized transcription to infer regulatory relationships among genes. Genome Biol. 2007;8(10):R219 10.1186/gb-2007-8-10-r219 17931418 PMC2246293

[28] Sun W, Yu T, Li KC. Detection of eQTL modules mediated by activity levels of transcription factors. Bioinformatics. 2007;23(17):2290–7. 10.1093/bioinformatics/btm327 . 17599927

[29] Greenland S, Pearl J, Robins JM. Causal diagrams for epidemiologic research. Epidemiology. 1999;10(1):37–48. . 9888278

[30] Yourshaw M, Taylor SP, Rao AR, Martin MG, Nelson SF. Rich annotation of DNA sequencing variants by leveraging the Ensembl Variant Effect Predictor with plugins. Brief Bioinform. 2015;16(2):255–64. 10.1093/bib/bbu008 . 24626529 PMC6283364

[31] Welter D, MacArthur J, Morales J, Burdett T, Hall P, Junkins H, et al The NHGRI GWAS Catalog, a curated resource of SNP-trait associations. Nucleic Acids Res. 2014;42(Database issue):D1001–6. 10.1093/nar/gkt1229 24316577 PMC3965119

[32] Bahr TM, Hughes GJ, Armstrong M, Reisdorph R, Coldren CD, Edwards MG, et al Peripheral blood mononuclear cell gene expression in chronic obstructive pulmonary disease. Am J Respir Cell Mol Biol. 2013;49(2):316–23. 10.1165/rcmb.2012-0230OC 23590301 PMC3824029

[33] Pruim RJ, Welch RP, Sanna S, Teslovich TM, Chines PS, Gliedt TP, et al LocusZoom: regional visualization of genome-wide association scan results. Bioinformatics. 2010;26(18):2336–7. 10.1093/bioinformatics/btq419 20634204 PMC2935401

[34] Ward LD, Kellis M. HaploReg: a resource for exploring chromatin states, conservation, and regulatory motif alterations within sets of genetically linked variants. Nucleic Acids Res. 2012;40(Database issue):D930–4. 10.1093/nar/gkr917 22064851 PMC3245002

[35] Amundadottir L, Kraft P, Stolzenberg-Solomon RZ, Fuchs CS, Petersen GM, Arslan AA, et al Genome-wide association study identifies variants in the ABO locus associated with susceptibility to pancreatic cancer. Nat Genet. 2009;41(9):986–90. 10.1038/ng.429 19648918 PMC2839871

[36] Band G, Le QS, Jostins L, Pirinen M, Kivinen K, Jallow M, et al Imputation-based meta-analysis of severe malaria in three African populations. PLoS Genet. 2013;9(5):e1003509 10.1371/journal.pgen.1003509 23717212 PMC3662650

[37] Barbalic M, Dupuis J, Dehghan A, Bis JC, Hoogeveen RC, Schnabel RB, et al Large-scale genomic studies reveal central role of ABO in sP-selectin and sICAM-1 levels. Hum Mol Genet. 2010;19(9):1863–72. 10.1093/hmg/ddq061 20167578 PMC2850624

[38] Chambers JC, Zhang W, Sehmi J, Li X, Wass MN, Van der Harst P, et al Genome-wide association study identifies loci influencing concentrations of liver enzymes in plasma. Nat Genet. 2011;43(11):1131–8. 10.1038/ng.970 22001757 PMC3482372

[39] Chu X, Pan CM, Zhao SX, Liang J, Gao GQ, Zhang XM, et al A genome-wide association study identifies two new risk loci for Graves' disease. Nat Genet. 2011;43(9):897–901. 10.1038/ng.898 . 21841780

[40] Chung CM, Wang RY, Chen JW, Fann CS, Leu HB, Ho HY, et al A genome-wide association study identifies new loci for ACE activity: potential implications for response to ACE inhibitor. Pharmacogenomics J. 2010;10(6):537–44. 10.1038/tpj.2009.70 . 20066004

[41] Comuzzie AG, Cole SA, Laston SL, Voruganti VS, Haack K, Gibbs RA, et al Novel genetic loci identified for the pathophysiology of childhood obesity in the Hispanic population. PLoS One. 2012;7(12):e51954 10.1371/journal.pone.0051954 23251661 PMC3522587

[42] de Boer RA, Verweij N, van Veldhuisen DJ, Westra HJ, Bakker SJ, Gansevoort RT, et al A genome-wide association study of circulating galectin-3. PLoS One. 2012;7(10):e47385 10.1371/journal.pone.0047385 23056639 PMC3467202

[43] Desch KC, Ozel AB, Siemieniak D, Kalish Y, Shavit JA, Thornburg CD, et al Linkage analysis identifies a locus for plasma von Willebrand factor undetected by genome-wide association. Proc Natl Acad Sci U S A. 2013;110(2):588–93. 10.1073/pnas.1219885110 23267103 PMC3545809

[44] Dichgans M, Malik R, Konig IR, Rosand J, Clarke R, Gretarsdottir S, et al Shared genetic susceptibility to ischemic stroke and coronary artery disease: a genome-wide analysis of common variants. Stroke. 2014;45(1):24–36. 10.1161/STROKEAHA.113.002707 24262325 PMC4112102

[45] Germain M, Saut N, Greliche N, Dina C, Lambert JC, Perret C, et al Genetics of venous thrombosis: insights from a new genome wide association study. PLoS One. 2011;6(9):e25581 10.1371/journal.pone.0025581 21980494 PMC3181335

[46] He M, Wu C, Xu J, Guo H, Yang H, Zhang X, et al A genome wide association study of genetic loci that influence tumour biomarkers cancer antigen 19–9, carcinoembryonic antigen and alpha fetoprotein and their associations with cancer risk. Gut. 2014;63(1):143–51. 10.1136/gutjnl-2012-303434 . 23300138

[47] Heit JA, Armasu SM, Asmann YW, Cunningham JM, Matsumoto ME, Petterson TM, et al A genome-wide association study of venous thromboembolism identifies risk variants in chromosomes 1q24.2 and 9q. J Thromb Haemost. 2012;10(8):1521–31. 10.1111/j.1538-7836.2012.04810.x 22672568 PMC3419811

[48] Kamatani Y, Matsuda K, Okada Y, Kubo M, Hosono N, Daigo Y, et al Genome-wide association study of hematological and biochemical traits in a Japanese population. Nat Genet. 2010;42(3):210–5. 10.1038/ng.531 . 20139978

[49] Kim YJ, Go MJ, Hu C, Hong CB, Kim YK, Lee JY, et al Large-scale genome-wide association studies in East Asians identify new genetic loci influencing metabolic traits. Nat Genet. 2011;43(10):990–5. 10.1038/ng.939 . 21909109

[50] Li J, Gui L, Wu C, He Y, Zhou L, Guo H, et al Genome-wide association study on serum alkaline phosphatase levels in a Chinese population. BMC Genomics. 2013;14:684 10.1186/1471-2164-14-684 24094242 PMC3851471

[51] Liang Y, Tang W, Huang T, Gao Y, Tan A, Yang X, et al Genetic variations affecting serum carcinoembryonic antigen levels and status of regional lymph nodes in patients with sporadic colorectal cancer from Southern China. PLoS One. 2014;9(6):e97923 10.1371/journal.pone.0097923 24941225 PMC4062418

[52] Naitza S, Porcu E, Steri M, Taub DD, Mulas A, Xiao X, et al A genome-wide association scan on the levels of markers of inflammation in Sardinians reveals associations that underpin its complex regulation. PLoS Genet. 2012;8(1):e1002480 10.1371/journal.pgen.1002480 22291609 PMC3266885

[53] Pare G, Ridker PM, Rose L, Barbalic M, Dupuis J, Dehghan A, et al Genome-wide association analysis of soluble ICAM-1 concentration reveals novel associations at the NFKBIK, PNPLA3, RELA, and SH2B3 loci. PLoS Genet. 2011;7(4):e1001374 10.1371/journal.pgen.1001374 21533024 PMC3080865

[54] Paterson AD, Lopes-Virella MF, Waggott D, Boright AP, Hosseini SM, Carter RE, et al Genome-wide association identifies the ABO blood group as a major locus associated with serum levels of soluble E-selectin. Arterioscler Thromb Vasc Biol. 2009;29(11):1958–67. 10.1161/ATVBAHA.109.192971 19729612 PMC3147250

[55] Porcu E, Medici M, Pistis G, Volpato CB, Wilson SG, Cappola AR, et al A meta-analysis of thyroid-related traits reveals novel loci and gender-specific differences in the regulation of thyroid function. PLoS Genet. 2013;9(2):e1003266 10.1371/journal.pgen.1003266 23408906 PMC3567175

[56] Qi L, Cornelis MC, Kraft P, Jensen M, van Dam RM, Sun Q, et al Genetic variants in ABO blood group region, plasma soluble E-selectin levels and risk of type 2 diabetes. Hum Mol Genet. 2010;19(9):1856–62. 10.1093/hmg/ddq057 20147318 PMC2850622

[57] Reilly MP, Li M, He J, Ferguson JF, Stylianou IM, Mehta NN, et al Identification of ADAMTS7 as a novel locus for coronary atherosclerosis and association of ABO with myocardial infarction in the presence of coronary atherosclerosis: two genome-wide association studies. Lancet. 2011;377(9763):383–92. 10.1016/S0140-6736(10)61996-4 21239051 PMC3297116

[58] Rueedi R, Ledda M, Nicholls AW, Salek RM, Marques-Vidal P, Morya E, et al Genome-wide association study of metabolic traits reveals novel gene-metabolite-disease links. PLoS Genet. 2014;10(2):e1004132 10.1371/journal.pgen.1004132 24586186 PMC3930510

[59] Schunkert H, Konig IR, Kathiresan S, Reilly MP, Assimes TL, Holm H, et al Large-scale association analysis identifies 13 new susceptibility loci for coronary artery disease. Nat Genet. 2011;43(4):333–8. 10.1038/ng.784 21378990 PMC3119261

[60] Shin SY, Fauman EB, Petersen AK, Krumsiek J, Santos R, Huang J, et al An atlas of genetic influences on human blood metabolites. Nat Genet. 2014;46(6):543–50. 10.1038/ng.2982 24816252 PMC4064254

[61] Smith NL, Huffman JE, Strachan DP, Huang J, Dehghan A, Trompet S, et al Genetic predictors of fibrin D-dimer levels in healthy adults. Circulation. 2011;123(17):1864–72. 10.1161/CIRCULATIONAHA.110.009480 21502573 PMC3095913

[62] Suhre K, Shin SY, Petersen AK, Mohney RP, Meredith D, Wagele B, et al Human metabolic individuality in biomedical and pharmaceutical research. Nature. 2011;477(7362):54–60. 10.1038/nature10354 21886157 PMC3832838

[63] Tang W, Schwienbacher C, Lopez LM, Ben-Shlomo Y, Oudot-Mellakh T, Johnson AD, et al Genetic associations for activated partial thromboplastin time and prothrombin time, their gene expression profiles, and risk of coronary artery disease. Am J Hum Genet. 2012;91(1):152–62. 10.1016/j.ajhg.2012.05.009 22703881 PMC3397273

[64] Tanikawa C, Urabe Y, Matsuo K, Kubo M, Takahashi A, Ito H, et al A genome-wide association study identifies two susceptibility loci for duodenal ulcer in the Japanese population. Nat Genet. 2012;44(4):430–4, S1-2. 10.1038/ng.1109 . 22387998

[65] Teslovich TM, Musunuru K, Smith AV, Edmondson AC, Stylianou IM, Koseki M, et al Biological, clinical and population relevance of 95 loci for blood lipids. Nature. 2010;466(7307):707–13. 10.1038/nature09270 20686565 PMC3039276

[66] Teupser D, Baber R, Ceglarek U, Scholz M, Illig T, Gieger C, et al Genetic regulation of serum phytosterol levels and risk of coronary artery disease. Circ Cardiovasc Genet. 2010;3(4):331–9. 10.1161/CIRCGENETICS.109.907873 . 20529992

[67] Timmann C, Thye T, Vens M, Evans J, May J, Ehmen C, et al Genome-wide association study indicates two novel resistance loci for severe malaria. Nature. 2012;489(7416):443–6. 10.1038/nature11334 . 22895189

[68] Tregouet DA, Heath S, Saut N, Biron-Andreani C, Schved JF, Pernod G, et al Common susceptibility alleles are unlikely to contribute as strongly as the FV and ABO loci to VTE risk: results from a GWAS approach. Blood. 2009;113(21):5298–303. 10.1182/blood-2008-11-190389 . 19278955

[69] van der Harst P, Zhang W, Mateo Leach I, Rendon A, Verweij N, Sehmi J, et al Seventy-five genetic loci influencing the human red blood cell. Nature. 2012;492(7429):369–75. 10.1038/nature11677 23222517 PMC3623669

[70] Williams FM, Carter AM, Hysi PG, Surdulescu G, Hodgkiss D, Soranzo N, et al Ischemic stroke is associated with the ABO locus: the EuroCLOT study. Ann Neurol. 2013;73(1):16–31. 10.1002/ana.23838 23381943 PMC3582024

[71] Yuan X, Waterworth D, Perry JR, Lim N, Song K, Chambers JC, et al Population-based genome-wide association studies reveal six loci influencing plasma levels of liver enzymes. Am J Hum Genet. 2008;83(4):520–8. 10.1016/j.ajhg.2008.09.012 18940312 PMC2561937

[72] Zhao SX, Xue LQ, Liu W, Gu ZH, Pan CM, Yang SY, et al Robust evidence for five new Graves' disease risk loci from a staged genome-wide association analysis. Hum Mol Genet. 2013;22(16):3347–62. 10.1093/hmg/ddt183 . 23612905

[73] Zhou L, He M, Mo Z, Wu C, Yang H, Yu D, et al A genome wide association study identifies common variants associated with lipid levels in the Chinese population. PLoS One. 2013;8(12):e82420 10.1371/journal.pone.0082420 24386095 PMC3875415

[74] Dimas AS, Deutsch S, Stranger BE, Montgomery SB, Borel C, Attar-Cohen H, et al Common regulatory variation impacts gene expression in a cell type-dependent manner. Science. 2009;325(5945):1246–50. 10.1126/science.1174148 19644074 PMC2867218

[75] Hoofnagle AN, Eckfeldt JH, Lutsey PL. Vitamin D-Binding Protein Concentrations Quantified by Mass Spectrometry. N Engl J Med. 2015;373(15):1480–2. 10.1056/NEJMc1502602 26397952 PMC4654614

[76] Cohen BH, Ball WC Jr., Brashears S, Diamond EL, Kreiss P, Levy DA, et al Risk factors in chronic obstructive pulmonary disease (COPD). Am J Epidemiol. 1977;105(3):223–32. . 300564 10.1093/oxfordjournals.aje.a112378

[77] Harrison GA, Boyce AJ, Hornabrook RW, Serjeantson S, Craig WJ. Evidence for an association between ABO blood group and goitre. Hum Genet. 1976;32(3):335–7. . 939553 10.1007/BF00295825

[78] Padma T, Valli VV. ABO blood groups, intestinal alkaline phosphatase and haptoglobin types in patients with serum hepatitis. Hum Hered. 1988;38(6):367–71. . 3246377 10.1159/000153816

[79] Obeidat M, Fishbane N, Nie Y, Chen V, Hollander Z, Tebbutt SJ, et al The Effect of Statins on Blood Gene Expression in COPD. PLoS One. 2015;10(10):e0140022 10.1371/journal.pone.0140022 26462087 PMC4604084

[80] Hansel NN, Pare PD, Rafaels N, Sin DD, Sandford A, Daley D, et al Genome-Wide Association Study Identification of Novel Loci Associated with Airway Responsiveness in Chronic Obstructive Pulmonary Disease. Am J Respir Cell Mol Biol. 2015;53(2):226–34. 10.1165/rcmb.2014-0198OC 25514360 PMC4566043

[81] Castaldi PJ, Cho MH, Litonjua AA, Bakke P, Gulsvik A, Lomas DA, et al The association of genome-wide significant spirometric loci with chronic obstructive pulmonary disease susceptibility. Am J Respir Cell Mol Biol. 2011;45(6):1147–53. 10.1165/rcmb.2011-0055OC 21659657 PMC3262664

[82] Hause RJ, Stark AL, Antao NN, Gorsic LK, Chung SH, Brown CD, et al Identification and validation of genetic variants that influence transcription factor and cell signaling protein levels. Am J Hum Genet. 2014;95(2):194–208. 10.1016/j.ajhg.2014.07.005 25087611 PMC4129400

[83] Horvatovich P, Franke L, Bischoff R. Proteomic studies related to genetic determinants of variability in protein concentrations. J Proteome Res. 2014;13(1):5–14. 10.1021/pr400765y . 24237071

[84] Yonchuk JG, Silverman EK, Bowler RP, Agusti A, Lomas DA, Miller BE, et al Circulating Soluble Receptor for Advanced Glycation End Products (sRAGE) as a Biomarker of Emphysema and the RAGE Axis in the Lung. Am J Respir Crit Care Med. 2015;192(7):785–92. 10.1164/rccm.201501-0137PP . 26132989

[85] Thun GA, Imboden M, Ferrarotti I, Kumar A, Obeidat M, Zorzetto M, et al Causal and synthetic associations of variants in the SERPINA gene cluster with alpha1-antitrypsin serum levels. PLoS Genet. 2013;9(8):e1003585 10.1371/journal.pgen.1003585 23990791 PMC3749935

[86] Miller RD, Kueppers F, Offord KP. Serum concentrations of C3 and C4 of the complement system in patients with chronic obstructive pulmonary disease. J Lab Clin Med. 1980;95(2):266–71. . 7354236

[87] Chauhan S, Gupta MK, Goyal A, Dasgupta DJ. Alterations in immunoglobulin & complement levels in chronic obstructive pulmonary disease. Indian J Med Res. 1990;92:241–5. . 2228068

[88] Nishioka M, Venkatesan N, Dessalle K, Mogas A, Kyoh S, Lin TY, et al Fibroblast-epithelial cell interactions drive epithelial-mesenchymal transition differently in cells from normal and COPD patients. Respir Res. 2015;16:72 10.1186/s12931-015-0232-4 26081431 PMC4473826

[89] Milara J, Peiro T, Serrano A, Cortijo J. Epithelial to mesenchymal transition is increased in patients with COPD and induced by cigarette smoke. Thorax. 2013;68(5):410–20. 10.1136/thoraxjnl-2012-201761 . 23299965

[90] Johansson A, Enroth S, Palmblad M, Deelder AM, Bergquist J, Gyllensten U. Identification of genetic variants influencing the human plasma proteome. Proc Natl Acad Sci U S A. 2013;110(12):4673–8. 10.1073/pnas.1217238110 23487758 PMC3606982

[91] Lourdusamy A, Newhouse S, Lunnon K, Proitsi P, Powell J, Hodges A, et al Identification of cis-regulatory variation influencing protein abundance levels in human plasma. Hum Mol Genet. 2012;21(16):3719–26. 10.1093/hmg/dds186 . 22595970 PMC6446535

