# Supplementary material for: Common Genetic Polymorphisms Influence Blood Biomarker Measurements in COPD
Source: PLoS Genet. 2016 Aug 17;12(8):e1006011. doi: 10.1371/journal.pgen.1006011 (PMC4988780; doi:10.1371/journal.pgen.1006011)
Supplement: S1 Table — LLOQ = Lower Limit of Quantification. All COPDGene samples were P100 plasma. Green cells under “BIOMARKER Variable Name” represent analytes that were evaluated in this work (those not analyzed had a high % below LLOQ). (DOCX) [file pgen.1006011.s001.docx]

| **S1 Table.** Analyte measurement details for SPIROMICS and COPDGene | | | | | | | | | | |
| --- | --- | --- | --- | --- | --- | --- | --- | --- | --- | --- |
| **BIOMARKER Variable Name** | **Biomarker name (SwissPro Protein Name)** | **Biomarker Name (NCBI Gene Name)** | **Units** | **SPIROMICS** | | | **COPDGene** | | | **SPIROMICS Sample Type** |
|  |  |  |  | **LLOQ** | **% below LLOQ** | **Median (25th, 75th percentile)** | **LLOQ** | **% below LLOQ** | **Median (25th, 75th percentile)** |  |
| A2M | Alpha-2-Macroglobulin (A2Macro) | Alpha-2-macroglobulin (A2M) | mg/mL | 0.02 | 0% | 1.6 (1.4, 2.0) | 0.023 | 0% | 1.0 (0.94, 1.2) | EDTA plasma |
| ADIPOQ | Adiponectin | Adiponectin, C1Q and collagen domain containing (ADIPOQ) | ug/mL | 0.05 | 0% | 5.9 (3.8, 9.2) | 0.051 | 0% | 5.3 (3.5, 8.1) | EDTA plasma |
| AGER | Receptor for advanced glycosylation end products (RAGE) | Advanced glycosylation end product-specific receptor (AGER) | ng/mL | 0.35 | 1% | 2.6 (1.6, 4.3) | 0.35 | 1.33% | 2.2 (1.4, 3.6) | EDTA plasma |
| ANGPT1 | Angiopoietin-1 (ANG-1) | Angiopoietin 1 (ANGPT1) | ng/mL | 2.60 | 0% | 35 (27, 44) | 2.1 | 0.50% | 7 (5.3, 9.3) | Serum |
| APCS | Serum Amyloid P-Component (SAP) | Amyloid P-component, serum (APCS) | ug/mL | 0.09 | 0% | 20 (16, 23) | 0.093 | 0% | 17 (14, 21) | EDTA plasma |
| APOA4 | Apolipoprotein A-IV (Apo A-IV) | Apolipoprotein A-IV (APOA4) | ug/mL | 0.96 | 0% | 12 (9.9, 15) | 1.6 | 0.17% | 7.8 (6.7, 9.1) | EDTA plasma |
| AXL | AXL Receptor Tyrosine Kinase (AXL) | AXL receptor tyrosine kinase (AXL) | ng/mL | 0.04 | 0% | 11(8.6, 13.0) | 0.05 | 0% | 12 (9.7, 15) | Serum |
| B2M | Beta-2-Microglobulin (B2M) | Beta-2-microglobulin (B2M) | ug/mL | 0.01 | 0% | 2 (1.6, 2.4) | 0.0069 | 0% | 1.8 (1.4, 2.3) | EDTA plasma |
| BDNF | Brain-Derived Neurotrophic Factor (BDNF) | Brain-derived neurotrophic factor (BDNF) | ng/mL | 0.06 | 0% | 2.9 (1.7, 4.9) | 0.062 | 0.50% | 3 (1.5, 5.5) | EDTA plasma |
| C3 | Complement C3 (C3) | Complement component C3 (C3) | mg/mL | 0.00 | 0% | 1.3 (1.1, 1.4) | 0.02 | 0% | 1.2 (1.1, 1.4) | EDTA plasma |
| CA9 | Carbonic anhydrase 9 (CA-9) | Carbonic anhydrase IX (CA9) | ng/mL | 0.18 | 83% | 0.09 (0.09, 0.09) | 0.22 | 43.28% | NA | Serum |
| CCL11 | Eotaxin-1 | Chemokine (C-C motif) ligand 11 (eotaxin-1) (CCL11) | pg/mL | 144.00 | 57% | 72 (72, 171) | 144 | 56.38% | NA | EDTA plasma |
| CCL13 | Monocyte Chemotactic Protein 4 (MCP-4) | Chemokine (C-C motif) ligand 13 (CCL13) | pg/mL | 972.00 | 2% | 1870 (1500, 2420) | 972 | 2.65% | 1640 (1340, 2130) | EDTA plasma |
| CCL16 | Chemokine CC-4 (HCC-4) | Chemokine (C-C motif) ligand 16 (CCL16) | ng/mL | 0.03 | 0% | 5.6 (4.0, 7.1) | 0.047 | 0% | 5.3 (4, 7) | Serum |
| CCL18 | Pulmonary and Activation-Regulated Chemokine (PARC) | Chemokine (C-C motif) ligand 18 (pulmonary and activation-regulated) (CCL18) | ng/mL | 5.80 | 0% | 99 (75, 134) | 6.2 | 0% | 89 (63, 120) | Serum |
| CCL2 | Monocyte Chemotactic Protein 1 (MCP-1) | Chemokine (C-C motif) ligand 2 (CCL2) | pg/mL | 24.00 | 0% | 125 (95, 161) | 45 | 0.33% | 139 (113, 176) | EDTA plasma |
| CCL20 | Macrophage Inflammatory Protein-3 alpha (MIP-3 alpha) | Chemokine (C-C motif) ligand 20 (CCL20) | pg/mL | 38.00 | 58% | 19 (19, 48) | 38 | 73.13% | NA | EDTA plasma |
| CCL22 | Macrophage-Derived Chemokine (MDC) | chemokine (C-C motif) ligand 22 (CCL22) | pg/mL | 19.00 | 0% | 603 (494, 746) | 19 | 0% | 400 (309, 488) | Serum |
| CCL23 | Myeloid Progenitor Inhibitory Factor 1 (MPIF-1) | Chemokine (C-C motif) ligand 23 (CCL23) | ng/mL | 0.18 | 0% | 1.4 (1.2, 1.7) | 0.18 | 0% | 1.4 (1.1, 1.7) | EDTA plasma |
| CCL24 | Eotaxin-2 | Chemokine (C-C motif) ligand 24 (CCL24) | pg/mL | 50.00 | 0% | 749 (480, 1270) | 50 | 0% | 553 (359, 844) | EDTA plasma |
| CCL3 | Macrophage Inflammatory Protein-1 alpha (MIP-1 alpha) | Chemokine (C-C motif) ligand 3 (CCL3) | pg/mL | 15.00 | 19% | 27 (18, 35) | 42 | 84.74% | NA | EDTA plasma |
| CCL4 | Macrophage Inflammatory Protein-1 beta (MIP-1 beta) | Chemokine (C-C motif) ligand 4 (CCL4) | pg/mL | 37.00 | 0% | 215 (165, 277) | 31 | 0% | 197 (152, 255) | EDTA plasma |
| CCL5 | T-Cell-Specific Protein RANTES (RANTES) | chemokine (C-C motif) ligand 5 (CCL5) | ng/mL | 0.02 | 0% | 11 (5.8, 18) | 0.024 | 0% | 9.8 (5.3, 17) | EDTA plasma |
| CCL8 | Monocyte Chemotactic Protein 2 (MCP-2) | Chemokine (C-C motif) ligand 8 (CCL8) | pg/mL | 8.60 | 1% | 30 (24, 36) | 8.6 | 0.33% | 27 (21, 34) | EDTA plasma |
| CDH1 | Cadherin-1 (E-Cad) | Cadherin, type 1, E-cadherin (epithelial) (CDH1) | ng/mL | 6.00 | 0% | 3510 (2810, 4380) | 6 | 0% | 3110 (2570, 4000) | EDTA plasma |
| CDH13 | Cadherin-13 (T-cad) | Cadherin 13, H-cadherin (heart) (CDH13) | ng/mL | 1.20 | 0% | 5.1 (4.4, 5.8) | 2.2 | 0% | 18 (15, 22) | Serum |
| CEACAM1 | Carcinoembryonic antigen-related cell adhesion molecule 1 (CEACAM1) | Carcinoembryonic antigen-related cell adhesion molecule 1 (CEACAM1) | ng/mL | 2.90 | 2% | 14 (11, 17) | 3.1 | 0% | 14 (12, 16) | Serum |
| CHGA | Chromogranin-A (CgA) | Chromogranin A (parathyroid secretory protein 1) (CHGA) | ng/mL | 13.00 | 0% | 431 (299, 795) | 13 | 0% | 485.5 (334, 775) | EDTA plasma |
| CKM_CKB | Creatine Kinase-MB (CK-MB) | Creatine kinase, muscle; Creatine kinase, brain (heterodimer) [CK(M/B)] | ng/mL | 0.35 | 0% | 1.9 (1.2, 2.8) | 0.35 | 0.50% | 1.4 (0.97, 2.1) | Serum |
| CRP | C-Reactive Protein (CRP) | C-reactive protein, pentraxin-related (CRP) | ug/mL | 0.05 | 0% | 3.1 (1.4, 6.6) | 0.048 | 0% | 2.7 (1.3, 6) | EDTA plasma |
| CSF2 | Granulocyte-Macrophage Colony-Stimulating Factor (GM-CSF) | Colony stimulating factor 2 (granulocyte-macrophage) (CSF2) | pg/mL | 71.00 | 100% | NA | 88 | 99.67% | NA | EDTA plasma |
| CSTB | Cystatin-B | Cystatin B (stefin B) (CSTB) | ng/mL | 0.34 | 0% | 12 (9.3, 16) | 0.34 | 0% | 9.4 (7.3, 12) | EDTA plasma |
| CXCL10 | Interferon gamma Induced Protein 10 (IP-10) | Chemokine (C-X-C motif) ligand 10 (CXCL10) | pg/mL | 100.00 | 0% | 293 (231, 397) | 100 | 0% | 262 (206, 354) | EDTA plasma |
| CXCL5 | Epithelial-Derived Neutrophil-Activating Protein 78 (ENA-78) | chemokine (C-X-C motif) ligand 5 chemokine 5 (CXCL5) | ng/mL | 0.08 | 0% | 2 (1.3, 3.0) | 0.084 | 1.00% | 0.7 (0.39, 1.2) | Serum |
| CXCL9 | Monokine Induced by Gamma Interferon (MIG) | Chemokine (C-X-C motif) ligand 9 (CXCL9) | pg/mL | 143.00 | 0% | 1150 (788, 1740) | 143 | 0% | 1020 (698, 1550) | EDTA plasma |
| DCN | Decorin | Decorin (DCN) | ng/mL | 0.22 | 0% | 1.9 (1.6, 2.2) | 0.13 | 0% | 1.9 (1.7, 2.2) | Serum |
| F7 | Factor VII | Coagulation factor VII (serum prothrombin conversion accelerator) (F7) | ng/mL | 1.80 | 0% | 568 (460, 688) | 1.8 | 0% | 563 (456, 688) | EDTA plasma |
| FABP3 |  | Fatty acid-binding protein 3, muscle and heart (mammary-derived growth inhibitor) (FABP3) | ng/mL | 6.90 | 43% | 7.9 (3.45, 12) | 6.9 | 57% | 3.5 (3.5, 9.7) | Serum |
| FAS | FASLG Receptor (FAS) | Fas cell surface death receptor (FAS) | ng/mL | 2.80 | 0% | 24 (19, 33) | 5.8 | 3.48% | 15 (11, 20) | Serum |
| FGA_FGB_FGG | Fibrinogen | Fibrinogen (trimer; alpha chain, beta chain, gamma chain) [FG(A/B/G)] | mg/mL | 0.03 | 0% | 5 (4.3, 5.7) | 0.049 | 0.17% | 4.2 (3.6, 4.8) | EDTA plasma |
| FTL_FTH1 | Ferritin (FRTN) | Ferritin [FT(L/H1)] | ng/mL | 4.30 | 1% | 106 (56, 193) | 4.3 | 0% | 120 (66, 218) | EDTA plasma |
| GC | Vitamin D-Binding Protein (VDBP) | Group-specific component (vitamin D binding protein) (GC) | ug/mL | 5.50 | 0% | 313 (248, 398) | 5.6 | 0% | 278 (220, 349) | EDTA plasma |
| HGF | Hepatocyte Growth Factor (HGF) | Hepatocyte growth factor (hepapoietin A; scatter factor) (HGF) | ng/mL | 0.66 | 0% | 13 (8.4, 20) | 1 | 1.33% | 5.6 (4.1, 6.8) | Serum |
| HP | Haptoglobin | Haptoglobin (HP) | mg/mL | 0.02 | 1% | 1.9 (1.4, 2.7) | 0.064 | 1.49% | 1.4 (0.94, 2.0) | EDTA plasma |
| HSPD1 | Heat Shock Protein 60 (HSP-60) | Heat shock 60kDa protein 1 (chaperonin) (HSPD1) | ng/mL | 45.00 | 97% | NA | 45 | 98.34% | NA | EDTA plasma |
| ICAM1 | Intercellular Adhesion Molecule 1 (ICAM-1) | Intercellular adhesion molecule 1 (ICAM1) | ng/mL | 1.50 | 0% | 133 (109, 161) | 1.5 | 0% | 125 (102, 151) | EDTA plasma |
| IFNG | Interferon gamma (IFN-gamma) | Interferon, gamma (IFNG) | pg/mL | 2.30 | 96% | NA | 1.5 | 8.62% | 3.2 (2.3, 4.3) | EDTA plasma |
| IGA | Immunoglobulin A (IgA) | Immunoglobulin A (IgA) | mg/mL | 0.05 | 0% | 2.4 (1.6, 3.3) | 0.056 | 0.33% | 2.0 (1.4, 2.8) | EDTA plasma |
| IGE | Immunoglobulin E (IgE) | Immunoglobulin E (IgE) | U/mL | 18.00 | 74% | 9 (9, 21) | 18 | 43.62% | NA | Serum |
| IGM | Immunoglobulin M (IgM) | Immunoglobulin M (IgM) | mg/mL | 0.14 | 0% | 1.7 (1.2, 2.6) | 0.094 | 0% | 1.6 (1.1, 2.3) | EDTA plasma |
| IL10 | Interleukin-10 (IL-10) | Interleukin 10 (IL10) | pg/mL | 5.10 | 74% | 2.55 (2.55, 5.3) | 6.9 | 90.55% | NA | EDTA plasma |
| IL12A_IL12B | Interleukin-12 Subunit p70 (IL-12p70) | Interleukin12 subunit p70 (IL12A/IL12B heterodimer) | pg/mL | 38.00 | 100% | NA | 38 | 99.34% | NA | EDTA plasma |
| IL12B | Interleukin-12 Subunit p40 (IL-12p40) | Interleukin 12B (natural killer cell stimulatory factor 2, cytotoxic lymphocyte maturation factor 2, p40) (IL12B) | ng/mL | 0.22 | 16% | 0.32 (0.25, 0.40) | 0.22 | 38.31% | NA | EDTA plasma |
| IL13 | Interleukin-13 (IL-13) | Interleukin 13 (IL13) | pg/mL | 6.20 | 100% | NA | 6.2 | 99.17% | NA | Serum |
| IL15 | Interleukin-15 (IL-15) | Interleukin 15 (IL15) | ng/mL | 0.39 | 89% | 0.195 (0.195, 0.195) | 0.39 | 58.37% | NA | EDTA plasma |
| IL16 | Interleukin-16 (IL-16) | Interleukin 16 (IL16) | pg/mL | 87.00 | 0% | 416 (336, 506) | 87 | 0% | 393 (330, 465) | Serum |
| IL17A | Interleukin-17 (IL-17) | Interleukin 17 (IL17A) | pg/mL | 2.90 | 92% | NA | 2.9 | 94.03% | NA | EDTA plasma |
| IL18 | Interleukin-18 (IL-18) | Interleukin 18 (interferon-gamma-inducing factor) (IL18) | pg/mL | 19.00 | 0% | 228 (176, 306) | 41 | 0.17% | 229 (169, 302) | EDTA plasma |
| IL18BP | Interleukin-18-binding protein (IL-18bp) | Interleukin 18 binding protein (IL18BP) | ng/mL | 0.05 | 0% | 15 (12, 19) | 0.096 | 0% | 11 (9.1, 14) | Serum |
| IL1A | Interleukin-1 alpha (IL-1 alpha) | Interleukin 1, alpha (IL1A) | ng/mL | 0.00 | 88% | 0.0006 (0.0006, 0.0006) | 0.0012 | 95.52% | NA | EDTA plasma |
| IL1B | Interleukin-1 beta (IL-1 beta) | Interleukin 1, beta (IL1B) | pg/mL | 4.80 | 92% | NA | 4.8 | 98.67% | NA | EDTA plasma |
| IL1RN | Interleukin-1 receptor antagonist (IL-1ra) | Interleukin 1 receptor antagonist (IL1RN) | pg/mL | 220.00 | 46% | 230 (110, 294) | 220 | 76.62% | NA | EDTA plasma |
| IL2 | Interleukin-2 (IL-2) | Interleukin 2 (IL2) | pg/mL | 8.90 | 98% | NA | 8.3 | 98.84% | NA | EDTA plasma |
| IL23A | Interleukin-23 (IL-23) | Interleukin 23, alpha subunit p19 (IL23A) | ng/mL | 0.68 | 22% | 0.95 (0.70, 1.2) | 0.68 | 75.95% | NA | EDTA plasma |
| IL2RA | Interleukin-2 receptor alpha (IL-2 receptor alpha) | Interleukin 2 receptor, alpha (IL2RA) | pg/mL | 420.00 | 0% | 2310 (1830, 2950) | 420 | 0% | 2100 (1713, 2668) | EDTA plasma |
| IL3 | Interleukin-3 (IL-3) | Interleukin 3 (IL3) | ng/mL | 0.02 | 100% | NA | 0.016 | 99.83% | NA | EDTA plasma |
| IL4 | Interleukin-4 (IL-4) | Interleukin 4 (IL4) | pg/mL | 28.00 | 99% | NA | 29 | 99.34% | NA | EDTA plasma |
| IL5 | Interleukin-5 (IL-5) | Interleukin 5 (colony stimulating factor, eosinophil) (IL5) | pg/mL | 10.00 | 100% | NA | 13 | 99.83% | NA | EDTA plasma |
| IL6 | Interleukin-6 (IL-6) | Interleukin 6 (interferon, beta 2) (IL6) | pg/mL | 7.20 | 95% | NA | 11 | 98.18% | NA | EDTA plasma |
| IL6R | Interleukin-6 receptor (IL-6r) | Interleukin-6 receptor (IL6R) | ng/mL | 0.02 | 0% | 29 (23, 34) | 0.018 | 0% | 28 (23, 34) | EDTA plasma |
| IL7 | Interleukin-7 (IL-7) | Interleukin 7 (IL7) | pg/mL | 12.00 | 97% | NA | 8.8 | 98.84% | NA | EDTA plasma |
| IL8 | Interleukin-8 (IL-8) | Interleukin 8 (IL8) | pg/mL | 2.60 | 1% | 8 (5.9, 10) | 4 | 1.33% | 9.4 (7.3, 13) | EDTA plasma |
| INS_INTACT | Proinsulin, Intact | Proinsulin, Intact (INS_intact) | pM | 7.10 | 93% | NA | 7.1 | 83.75% | NA | EDTA plasma |
| INS_TOTAL | Proinsulin, Total | Proinsulin, Total (INS_total) | pM | 34.00 | 93% | NA | 34 | 83.75% | NA | EDTA plasma |
| KIT | Mast/stem cell growth factor receptor (SCFR) | v-kit Hardy-Zuckerman 4 feline sarcoma viral oncogene homolog (KIT) | ng/mL | 0.51 | 0% | 9 (7.6, 11) | 0.51 | 0% | 8.1 (6.8, 9.3) | EDTA plasma |
| KITLG | Stem Cell Factor (SCF) | KIT ligand (KITLG) | pg/mL | 119.00 | 1% | 345 (283, 415) | 119 | 0.83% | 301 (237, 367) | EDTA plasma |
| KLK3_F | Prostate-Specific Antigen, Free (PSA-f) | Kallikrein-related peptidase 3 (free) (PSAF) | ng/mL | 0.01 | 54% | 0.0065 (0.01, 0.08) | 0.013 | 49.75% | NA | EDTA plasma |
| LPA | Apolipoprotein(a) (Lp(a)) | Lipoprotein, (Lp(a) (LPA) | ug/mL | 1.60 | 0% | 203 (80, 568) | 0.96 | 0% | 167 (66, 460) | EDTA plasma |
| LTA | Tumor Necrosis Factor beta (TNF-beta) | Lymphotoxin alpha (LTA) | pg/mL | 27.00 | 99% | NA | 9.7 | 98.51% | NA | EDTA plasma |
| LTF | Lactoferrin (LTF) | Lactotransferrin | ng/mL | 6.40 | 2% | 15 (11, 20) | 6.4 | 4.15% | 14 (10, 19) | EDTA plasma |
| MB | Myoglobin | Myoglobin (MB) | ng/mL | 2.10 | 0% | 43 (32, 63) | 2.1 | 0% | 34 (25, 48) | EDTA plasma |
| MDA_LDL | Malondialdehyde-Modified Low-Density Lipoprotein (MDA-LDL) | Malondialdehyde-modified low-density lipoprotein (MDA-LDL) | ng/mL | 22.00 | 83% | 11 (11, 11) | 22 | 82.42% | NA | Serum |
| MDK | Midkine | Midkine (neurite growth-promoting factor 2) (MDK) | ng/mL | 1.40 | 43% | 1.6 (0.70, 2.2) | 0.46 | 0.66% | 2 (1.6, 2.5) | Serum |
| MICA | MHC class I chain-related protein A (MICA) | MHC class I polypeptide-related sequence A (MICA) | pg/mL | 73.00 | 56% | 36.5 (36.5, 100) | 73 | 45.94% | NA | EDTA plasma |
| MMP3 | Matrix Metalloproteinase-3 (MMP-3) | Matrix metalloproteinase 3 (stromelysin 1, progelatinase) (MMP3) | ng/mL | 0.05 | 0% | 8.6 (6.2, 12) | 0.049 | 0% | 8.2 (5.6, 12) | EDTA plasma |
| MMP9 | Matrix Metalloproteinase-9 (MMP-9) | Matrix Metalloproteinase 9 (gelatinase B, 92kDA type IV collagenase) (MMP9) | ng/mL | 37.00 | 3% | 188 (109, 324) | 37 | 0.17% | 299 (201, 451) | EDTA plasma |
| NGF | Nerve Growth Factor beta (NGF-beta) | Nerve growth gactor (beta polypeptide) (NGF) | ng/mL | 0.08 | 100% | NA | 0.078 | 100% | NA | EDTA plasma |
| NPPB_PH | N-terminal prohormone of brain natriuretic peptide (NT proBNP) | Natriuretic peptide (NPPB; N-terminal prohormone) | pg/mL | 16.00 | 0% | 393 (188, 798) | 16 | 0.17% | 460 (235, 838) | Serum |
| NRCAM | Neuronal Cell Adhesion Molecule (Nr-CAM) | Neuronal cell adhesion molecule (NRCAM) | ng/mL | 0.20 | 1% | 0.96 (0.73, 1.3) | 0.2 | 0.17% | 0.9 (0.70, 1.2) | EDTA plasma |
| OLR1 | Lectin-Like Oxidized LDL Receptor 1 (LOX-1) | Oxidized low density lipoprotein (lectin-like) receptor 1 (OLR1) | ng/mL | 0.75 | 17% | 1.2 (0.85, 1.8) | 0.75 | 94.53% | NA | Serum |
| PECAM1 | Platelet endothelial cell adhesion molecule (PECAM-1) | Platelet endothelial cell adhesion molecule (PECAM1) | ng/mL | 18.00 | 0% | 53 (46, 63) | 11 | 0% | 44 (37, 52) | Serum |
| S100B | S100 calcium-binding protein B (S100-B) | S100 calcium binding protein B (S100B) | ng/mL | 0.50 | 100% | NA | 0.5 | 100% | NA | EDTA plasma |
| SELE | E-Selectin | Selectin E (SELE) | ng/mL | 0.31 | 0% | 7.7 (5.6, 10) | 0.31 | 0% | 7.6 (5.6, 10) | EDTA plasma |
| SERPINA1 | Alpha-1-Antitrypsin (AAT) | Serpin peptidase inhibitor, clade A (alpha-1 antiproteinase, antitrypsin) (SERPINA1) | mg/mL | 0.01 | 0% | 1.9 (1.7, 2.2) | 0.016 | 0% | 1.8 (1.6, 2.1) | EDTA plasma |
| SERPINA3 | Alpha-1-Antichymotrypsin (AACT) | Serpin peptidase inhibitor, clade A (alpha-1 antiproteinase, antitrypsin), member 3 (SERPINA3) | ug/mL | 13.00 | 0% | 758 (661, 880) | 13 | 0.17% | 748 (665, 861) | EDTA plasma |
| SERPINA7 | Thyroxine-Binding Globulin (TBG) | Serpin peptidase inhibitor, clade A (alpha-1 antiproteinase, antitrypsin), member 7 (SERPINA7) | ug/mL | 0.22 | 0% | 40 (34, 46) | 0.22 | 0% | 37 (32, 42) | EDTA plasma |
| SERPINE1 | Plasminogen Activator Inhibitor 1 (PAI-1) | Serpin peptidase inhibitor, clade E (nexin, plasminogen activator inhibitor 1 type 1), member 1 (SERPINE1) | ng/mL | 2.80 | 0% | 44 (28, 66) | 2.8 | 0.83% | 34 (21, 49.5) | EDTA plasma |
| SFTPD | Pulmonary surfactant-associated protein D (SP-D) | Surfactant protein D (SFTPD) | ng/mL | 0.57 | 0% | 15 (10, 21) | 0.19 | 0% | 6.6 (4.7, 8.8) | Serum |
| SHBG | Sex Hormone-Binding Globulin (SHBG) | Sex hormone-binding globulin (SHBG) | nmol/L | 3.10 | 0% | 59 (42, 86) | 3.1 | 0% | 54 (39, 77) | EDTA plasma |
| SLPI | Antileukoproteinase (ALP) | Secretory leukocyte peptidase inhibitor (SLPI) | ng/mL | 0.98 | 0% | 42 (37, 49) | 0.98 | 0% | 37 (33, 42) | EDTA plasma |
| SOD1 | Superoxide Dismutase 1, soluble (SOD-1) | Superoxide dismutase 1, soluble (SOD1) | ng/mL | 0.12 | 0% | 35 (26, 48) | 0.12 | 0% | 32 (25, 41) | EDTA plasma |
| SORT1 | Sortilin | Sortilin 1 (SORT1) | ng/mL | 0.22 | 0% | 4.5 (3.8, 5.5) | 0.22 | 0% | 5.8 (4.9, 6.9) | EDTA plasma |
| SPINK1 | Pancreatic secretory trypsin inhibitor (TATI) | Serine peptidase inhibitor, Kazal type 1 (SPINK1) | ng/mL | 0.16 | 0% | 15 (12, 22) | 0.16 | 0% | 13 (10, 18) | EDTA plasma |
| TGFB1_LAP | Latency-Associated Peptide of Transforming Growth Factor beta 1 (LAP TGF-b1) | Transforming growth factor, beta 1 (TGFB1; latency associated peptide) | ng/mL | 0.13 | 0% | 4.2 (2.8, 6.2) | 0.13 | 0% | 3.8 (2.5, 6.2) | EDTA plasma |
| THBD | Thrombomodulin (TM) | Thrombomodulin (THBD) | ng/mL | 0.05 | 0% | 4.6 (3.9, 5.6) | 0.052 | 0% | 4.4 (3.8, 5.2) | Serum |
| TIMP1 | Tissue Inhibitor of Metalloproteinases 1 (TIMP-1) | TIMP metallopeptidase inhibitor 1 (TIMP1) | ng/mL | 1.20 | 0% | 84 (72, 99) | 1.2 | 0% | 72 (63, 87) | EDTA plasma |
| TIMP2 | Tissue Inhibitor of Metalloproteinases 2 (TIMP-2) | TIMP metallopeptidase inhibitor 2 (TIMP2) | ng/mL | 1.50 | 0% | 75 (67, 84) | 1.5 | 0% | 66 (59, 73) | EDTA plasma |
| TNF | Tumor Necrosis Factor alpha (TNF-alpha) | Tumor necrosis factor (TNF) | pg/mL | 18.00 | 96% | NA | 23 | 97.68% | NA | EDTA plasma |
| TNFRSF10C | TNF-Related Apoptosis-Inducing Ligand Receptor 3 (TRAIL-R3) | Tumor necrosis factor receptor superfamily, member 10c, decoy without an intracellular domain (TNFRSF10C) | ng/mL | 0.28 | 0% | 17 (12, 22) | 0.96 | 0% | 13 (9.1, 17) | Serum |
| TNFRSF11B | Osteoprotegerin (OPG) | Tumor necrosis factor receptor superfamily, member 11b (TNFRSF11B) | pM | 0.45 | 0% | 5.5 (4.6, 6.6) | 0.45 | 0% | 5.5 (4.5, 6.6) | EDTA plasma |
| TNFRSF1A | Tumor Necrosis Factor Receptor I (TNF RI) | Tumor necrosis factor receptor superfamily, member 1A (TNFRSF1A) | pg/mL | 36.00 | 0% | 1800 (1410, 2280) | 36 | 0% | 1630 (1350, 2018) | EDTA plasma |
| TNFRSF1B | Tumor necrosis factor receptor 2 (TNFR2) | Tumor necrosis factor receptor superfamily, member 1B (TNFRSF1B) | ng/mL | 0.86 | 0% | 7 (5.6, 8.9) | 0.86 | 0% | 5.6 (4.6, 7.1) | EDTA plasma |
| VCAM1 | Vascular Cell Adhesion Molecule-1 (VCAM-1) | Vascular cell adhesion molecule 1 (VCAM1) | ng/mL | 2.40 | 0% | 582 (498, 699) | 2.4 | 0% | 505 (431, 598) | EDTA plasma |
| VEGFA | Vascular Endothelial Growth Factor (VEGF) | Vascular endothelial growth factor (VEGFA) | pg/mL | 50.00 | 1% | 128 (100, 158) | 50 | 1.33% | 115 (92, 149) | EDTA plasma |
| VWF | von Willebrand Factor (vWF) | von Willebrand factor (VWF) | ug/mL | 25.00 | 0% | 99 (78, 127) | 25 | 1.16% | 77 (58, 102) | EDTA plasma |

. LLOQ=Lower Limit of Quantification. All COPDGene samples were P100 plasma. Green cells under “BIOMARKER Variable Name” represent analytes that were evaluated in this work (those not analyzed had a high % below LLOQ).
