# Supplementary material for: Common Genetic Polymorphisms Influence Blood Biomarker Measurements in COPD
Source: PLoS Genet. 2016 Aug 17;12(8):e1006011. doi: 10.1371/journal.pgen.1006011 (PMC4988780; doi:10.1371/journal.pgen.1006011)
Supplement: S2 Table — (DOCX) [file pgen.1006011.s002.docx]

| **S2 Table. Demographic features of cohort in current manuscript compared to comparable non-Hispanic White cohort data from the larger study cohorts.** | | | | |
| --- | --- | --- | --- | --- |
| **Characteristic** | **SPIROMICS**  **for present**  **manuscript** | **SPIROMICS**  **overall** | **COPDGene**  **for present**  **manuscript** | **COPDGene**  **overall** |
| No. of participants | 750 | 2100 | 590 | 6884 |
| Age at study entry ̶ yr  Mean  [25^th^,75^th^ percentiles] | 66.5 ±7.9  [63.0, 72.0] | 64.6 ±8.6  [59, 71] | 63.7 ±8.6  [57.4, 70.4] | 62.0 ±8.9  [55.1, 68.7] |
| Male sex ̶ no. (%) | 408 (54) | 1139 (54) | 304 (52) | 3598 (52) |
| Current Smoker ̶ no. (%) | 230 (31) | 945 (31) | 142 (24) | 2680 (39) |
| BMI ̶ kg/m^2^  Mean  [25^th^,75^th^ percentiles] | 27.7 ±5.0  [23.9, 31.3] | 27.8 ±5.0  [24.2, 31.2] | 28.3 ±5.6  [24.4, 31.2] | 28.7 ±6.0  [24.4, 32.0] |
| ATS Smoking pack-yrs  Mean  [25^th^,75^th^ percentiles] | 52.6 ±24.4  [35.0, 64.5] | 48.2 ±28.2  [30, 60] | 47.5 ±26.6  [28.5, 61.4] | 47.2 ±26.0  [30.0, 58.6] |
| Emphysema ̶ % -950 HU  Mean  [25^th^,75^th^ percentiles] | 8.7 ±10.3  [1.4, 12.1] | 7.8 ±10.0  [1.1, 10.5] | 9.6 ±11.8  [1.1, 14.7] | 7.3 ±10.2  [0.8, 8.9] |
| FEV_1_ ̶ % predicted  Mean  [25^th^,75^th^ percentiles] | 71.0 ±25.4  [51.7, 90.0] | 73.6 ±26.5  [53.8, 94.0] | 68.2 ±29.8  [42.8, 94.0] | 74.0 ±26.0  [55.4, 93.5] |
| FEV_1_/FVC  Mean  [25^th^,75^th^ percentiles] | 0.59 ±0.16  [0.48, 0.72] | 0.61 ±0.17  [0.5, 0.75] | 0.58 ±0.19  [0.41, 0.76] | 0.64 ±0.16  [0.54, 0.77] |
| Exacerbations in year prior to baseline - Mean | 0.35 ±0.83 | 0.37 ±0.85 | 0.59 ±1.2 | 0.43 ±0.98 |
| Chronic Bronchitis (%) | 157 (22) | 422 (21) | 113 (19) | 1415 (21) |
