# Supplementary material for: Common Genetic Polymorphisms Influence Blood Biomarker Measurements in COPD
Source: PLoS Genet. 2016 Aug 17;12(8):e1006011. doi: 10.1371/journal.pgen.1006011 (PMC4988780; doi:10.1371/journal.pgen.1006011)
Supplement: S5 Table — The method utilized to determine these pQTLS is described in detail in the Methods (recursive conditioning). Results for both COPDGene and SPIROMICS are shown. None = all pQTLs are in strong linkage disequilibrium with the top SNP. (DOCX) [file pgen.1006011.s005.docx]

**S5 Table.** pQTL SNPs that show independent evidence for association with blood analyte levels as compared to the top reported eQTL SNP.

| **Protein name {alternative name(s)}** | **Gene** | **Top SNP** | **SNPs Adding Additional Value (independently significant after conditioning)** | |
| --- | --- | --- | --- | --- |
|  |  |  | **COPDGene** | **SPIROMICS** |
| Advanced glycosylation end product-specific receptor {RAGE} | *AGER* | rs2070600 | None | None |
| Apolipoprotein A-IV {Apo A-IV} | *APOA4* | rs4938353 | None | rs10892063 |
| Complement component 3 | *C3* | rs2230203 | None | None |
| Chemokine (C-C motif) ligand 16 (pulmonary and activation-regulated) | *CCL16* | rs11080369 | None | rs854685; rs2063979 |
| C-C motif chemokine 18 | *CCL18* | rs854469 | None | rs9892586 |
| Chemokine (C-C motif) ligand 23 {Myeloid Progenitor Inhibitory Factor 1; MPIF-1} | *CCL23* | rs1617208 | rs7217473 | rs854678; rs854625 |
| Chemokine (C-C motif) ligand 24 {Eotaxin-2} | *CCL24* | rs10755885 | rs11465307; rs2024049 | rs6946822; rs11465307 |
| Chemokine (C-C motif) ligand 4 {Macrophage Inflammatory Protein-1 ß; MIP-1 ß} | *CCL4* | rs3213636 | rs34045601; rs4796217 | rs34045601; rs8064426 |
| Chemokine (C-C motif) ligand 8 {Monocyte Chemotactic Protein 2; MCP-2} | *CCL8* | rs3848464 | rs365654 | rs9914367 |
| Cadherin-1 {E-cadherin (epithelial)} | *CDH1* | rs516246 | rs507666 | rs579459 |
| Cystatin-B | *CSTB* | rs1041456 | rs13052408; rs4819314 | rs13052408 |
| C-X-C motif chemokine 5 {Epithelial-Derived Neutrophil-Activating Protein 78; ENA-78)} | *CXCL5* | rs425535 | None | None |
| Coagulation Factor VII | *F7* | rs10665 | None | rs555212 |
| Tumor necrosis factor receptor superfamily member 6 {FASLG Receptor; CD95} | *FAS* | rs687289 | None | rs507666 |
| Vitamin D-Binding Protein | *GC* | *rs7041* | rs4694105 | rs4588 |
| Hepatocyte Growth Factor | *HGF* | rs687289 | None | rs507666; rs8176743 |
| Haptoglobin | *HP* | rs1424241 | rs1050362 | rs2240243 |
| Interleukin-12 Subunit p40 {IL-12p40} | *IL12B* | rs10045431 | None | None |
| Interleukin-16 | *IL16* | rs1803275 | None | rs3848180 |
| Interleukin-18 | *IL18* | rs7577696 | None | None |
| Interleukin-23A | IL23A | rs10665 | None | None |
| Interleukin-2 receptor subunit alpha | *IL2RA* | rs12722489 | rs7898880 | rs11594656 |
| Interleukin-6 receptor subunit alpha | *IL6R* | rs8192284 | rs4845618; rs751899 | rs4845625 |
| Apolipoprotein(a) | *LPA* | rs9457925 | rs11751605 | rs3120137; rs783147 |
| Lactotransferrin | *LTF* | rs11707471 | None | rs7430431 |
| MHC class I polypeptide-related sequence A | *MICA* | rs2256175 | rs9263871 | rs28367646 |
| Stromelysin-1 {Matrix Metalloproteinase-3; MMP-3} | *MMP3* | rs645419 | None | None |
| Neuronal Cell Adhesion Molecule | *NRCAM* | rs10487851 | None | rs2267887 |
| Platelet endothelial cell adhesion molecule | *PECAM1* | rs507666 | rs7503550; rs8176746 | rs7503550; rs8176743 |
| E-Selectin | *SELE* | rs507666 | rs9411381 | rs687289 |
| Alpha-1-Antitrypsin | *SERPINA1* | rs4905179 | rs998520 | rs998520 |
| Pulmonary surfactant-associated protein D {SP-D} | *SFTPD* | *rs2146192* | rs7084667; rs9266629 | rs7084667; rs9266629 |
| Sex Hormone-Binding Globulin | *SHBG* | rs727428 | None | rs1799941 |
| Sortilin | *SORT1* | rs7528419 | None | None |
| Pancreatic secretory trypsin inhibitor {TATI} | *SPINK1* | rs6580502 | None | None |
| Tumor necrosis factor receptor superfamily member 10C {TNF-Related Apoptosis-Inducing Ligand Receptor 3; TRAIL-R3)} | *TNFRSF10C* | *rs4760* | None | None |
| Vascular Endothelial Growth Factor A | *VEGFA* | rs7767396 | None | None |
| von Willebrand Factor | *VWF* | rs687289 | None | None |

Protein names are UniProKB/Swiss-Prot suggested names. The method utilized to determine these pQTLS is described in detail in the Methods (recursive conditioning). Results for both COPDGene and SPIROMICS are shown. None = all pQTLs are in strong linkage disequilibrium with the top SNP.
