# Supplementary material for: Common Genetic Polymorphisms Influence Blood Biomarker Measurements in COPD
Source: PLoS Genet. 2016 Aug 17;12(8):e1006011. doi: 10.1371/journal.pgen.1006011 (PMC4988780; doi:10.1371/journal.pgen.1006011)
Supplement: S6 Table — Only those for HP (red) were also pQTLs (S4 Table). CDH1 and PECAM eQTLs are local, while pQTLs for these two analytes were distant. RNA expression levels of PECAM1 are measured by two different ProbeSetIDs in the Affymetrix arrays used for the gene expression studies. For HP, the model that best fits the evidence is listed. Causal in this case indicates that the evidence supports gene expression levels producing altered protein levels. Modeling for HP was conducted as for Fig 8 in the main manuscript using HP levels in place of disease. (DOCX) [file pgen.1006011.s006.docx]

| **S6 Table.** Significant expression eQTLs for the blood biomarkers tested in this study (as described in methods). | | | | |
| --- | --- | --- | --- | --- |
| **Gene_ProbeSetID** | **eQTL SNP** | **eQTL Chr** | **eQTL P-value** | **Type/Model** |
| CDH1_201131_s_at | rs2274240 | 16 | 6.16E-11 |  |
| CDH1_201131_s_at | rs8059194 | 16 | 8.08E-11 |  |
| CDH1_201131_s_at | rs17715450 | 16 | 1.07E-10 |  |
| CDH1_201131_s_at | rs11642413 | 16 | 3.66E-10 |  |
| HP_206697_s_at | rs8062041 | 16 | 1.07E-17 |  |
| **HP_206697_s_at** | **rs1050362** | **16** | **1.07E-17** | **Causal** |
| **HP_206697_s_at** | **rs2240243** | **16** | **1.07E-17** | **Causal** |
| **HP_206697_s_at** | **rs2074626** | **16** | **1.07E-17** | **Causal** |
| **HP_206697_s_at** | **rs6680** | **16** | **4.02E-16** | **Causal** |
| **HP_206697_s_at** | **rs1035559** | **16** | **4.76E-13** | **eQTL only** |
| **HP_206697_s_at** | **rs11864453** | **16** | **4.76E-13** | **eQTL only** |
| HP_206697_s_at | rs2303285 | 16 | 6.70E-12 |  |
| HP_206697_s_at | rs7186207 | 16 | 3.38E-11 |  |
| HP_206697_s_at | rs3213422 | 16 | 5.13E-11 |  |
| PECAM1_1558397_at | rs1050382 | 17 | 1.77E-22 |  |
| PECAM1_1558397_at | rs2812 | 17 | 2.36E-18 |  |
| PECAM1_1558397_at | rs9303470 | 17 | 3.42E-16 |  |
| PECAM1_1558397_at | rs6504218 | 17 | 3.42E-16 |  |
| PECAM1_1558397_at | rs7213889 | 17 | 7.78E-16 |  |
| PECAM1_1558397_at | rs1867625 | 17 | 1.77E-15 |  |
| PECAM1_1558397_at | rs1108591 | 17 | 5.08E-14 |  |
| PECAM1_1558397_at | rs4968620 | 17 | 5.63E-13 |  |
| PECAM1_1558397_at | rs12939215 | 17 | 1.12E-12 |  |
| PECAM1_1558397_at | rs1470453 | 17 | 4.68E-12 |  |
| PECAM1_208982_at | rs1050382 | 17 | 1.26E-18 |  |
| PECAM1_208982_at | rs2812 | 17 | 9.14E-15 |  |
| PECAM1_208982_at | rs9303470 | 17 | 1.69E-14 |  |
| PECAM1_208982_at | rs6504218 | 17 | 1.69E-14 |  |
| PECAM1_208982_at | rs7213889 | 17 | 3.15E-11 |  |
| PECAM1_208982_at | rs1108591 | 17 | 1.60E-10 |  |
| Only those for HP (red) were also pQTLs (Table S3). CDH1 and PECAM eQTLs are local, while pQTLs for these two analytes were distant. RNA expression levels of PECAM1 are measured by two different ProbeSetIDs in the Affymetrix arrays used for the gene expression studies. For HP, the model that best fits the evidence is listed. Causal in this case indicates that the evidence supports gene expression levels producing altered protein levels. Modeling for HP was conducted as for Figure 7A in the main manuscript using HP levels in place of disease. | | | | |
