# Supplementary material for: Common Genetic Polymorphisms Influence Blood Biomarker Measurements in COPD
Source: PLoS Genet. 2016 Aug 17;12(8):e1006011. doi: 10.1371/journal.pgen.1006011 (PMC4988780; doi:10.1371/journal.pgen.1006011)
Supplement: S1 Fig — A) Comparison of selected biomarker values on two different platforms by Quotient Bio Research (QBR) and Myriad Rules Based Medicine (RBM) from a selected subset of COPDGene subjects. The correlation coefficients are shown in the upper right panel and the scatterplots in the lower left panel. A histogram of the biomarker values are shown on the diagonal plots. B) Comparison of an R&D Quantikine ELISA (X axis) from serum of selected SPIROMICS subjects to the RBM (Y axis) for vitamin D binding protein. The two assays are highly concordant. See methods for details. (DOCX) [file pgen.1006011.s009.docx]

| 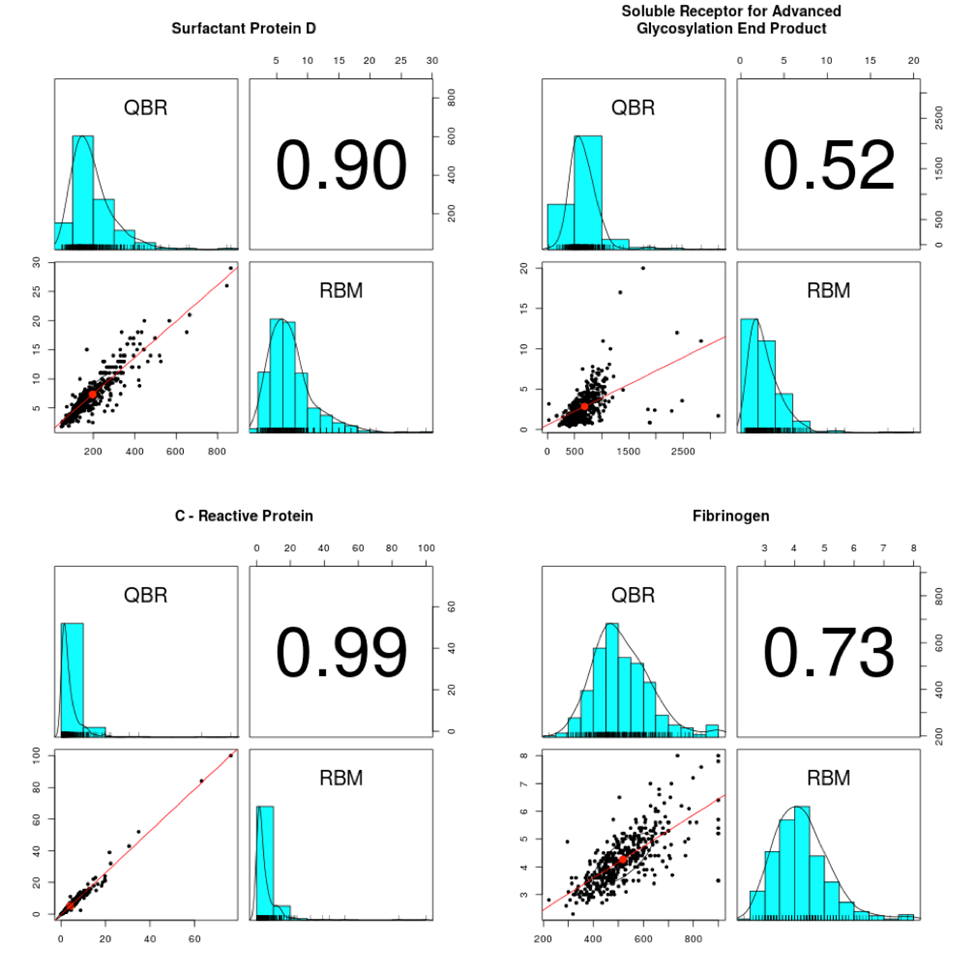  A. |
| --- |
| 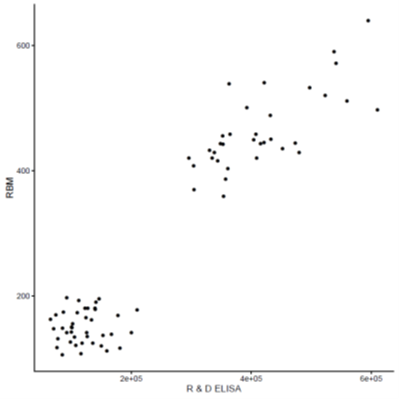  B. |
| **S1 Fig.** Examples of assay validation. A) Comparison of selected biomarker values on two different platforms by Quotient Bio Research (QBR) and Myriad Rules Based Medicine (RBM) from a selected subset of COPDGene subjects. The correlation coefficients are shown in the upper right panel and the scatterplots in the lower left panel. A histogram of the biomarker values are shown on the diagonal plots. B) Comparison of an R&D Quantikine ELISA (X axis) from serum of selected SPIROMICS subjects to the RBM (Y axis) for vitamin D binding protein. The two assays are highly concordant. See methods for details. |
