# Supplementary material for: Common Genetic Polymorphisms Influence Blood Biomarker Measurements in COPD
Source: PLoS Genet. 2016 Aug 17;12(8):e1006011. doi: 10.1371/journal.pgen.1006011 (PMC4988780; doi:10.1371/journal.pgen.1006011)
Supplement: S2 Fig — We ran PCA on biomarker data after regressing out all the other covariates used in pQTL analyses. Based on the sizes of eigen-values shown in this plot, we choose to include the first PC into our pQTL analysis to account for unobserved confounding effects. (DOCX) [file pgen.1006011.s010.docx]

**S2 Fig.** Barplot of eigen-values of PCA analysis in SPIROMICS biomarker data. We ran PCA on biomarker data after regressing out all the other covariates used in pQTL analyses. Based on the sizes of eigen-values shown in this plot, we choose to include the first PC into our pQTL analysis to account for unobserved confounding effects.
