# Supplementary material for: Common Genetic Polymorphisms Influence Blood Biomarker Measurements in COPD
Source: PLoS Genet. 2016 Aug 17;12(8):e1006011. doi: 10.1371/journal.pgen.1006011 (PMC4988780; doi:10.1371/journal.pgen.1006011)
Supplement: S3 Fig — SPIROMICS (top) and COPDGene (bottom) for (A) chronic bronchitis (0 = no; 1 = yes), (B) frequency of exacerbations in the 12 months prior to enrollment (exacerbations include respiratory events that required doctor visit, emergency room visit, hospitalization, or a change in antibiotic or steroid use), (C) FEV1% predicted, (D) percent total lung emphysema as defined by Hounsfield units -950; and (E) log transformation of percent emphysema. (DOCX) [file pgen.1006011.s011.docx]

| 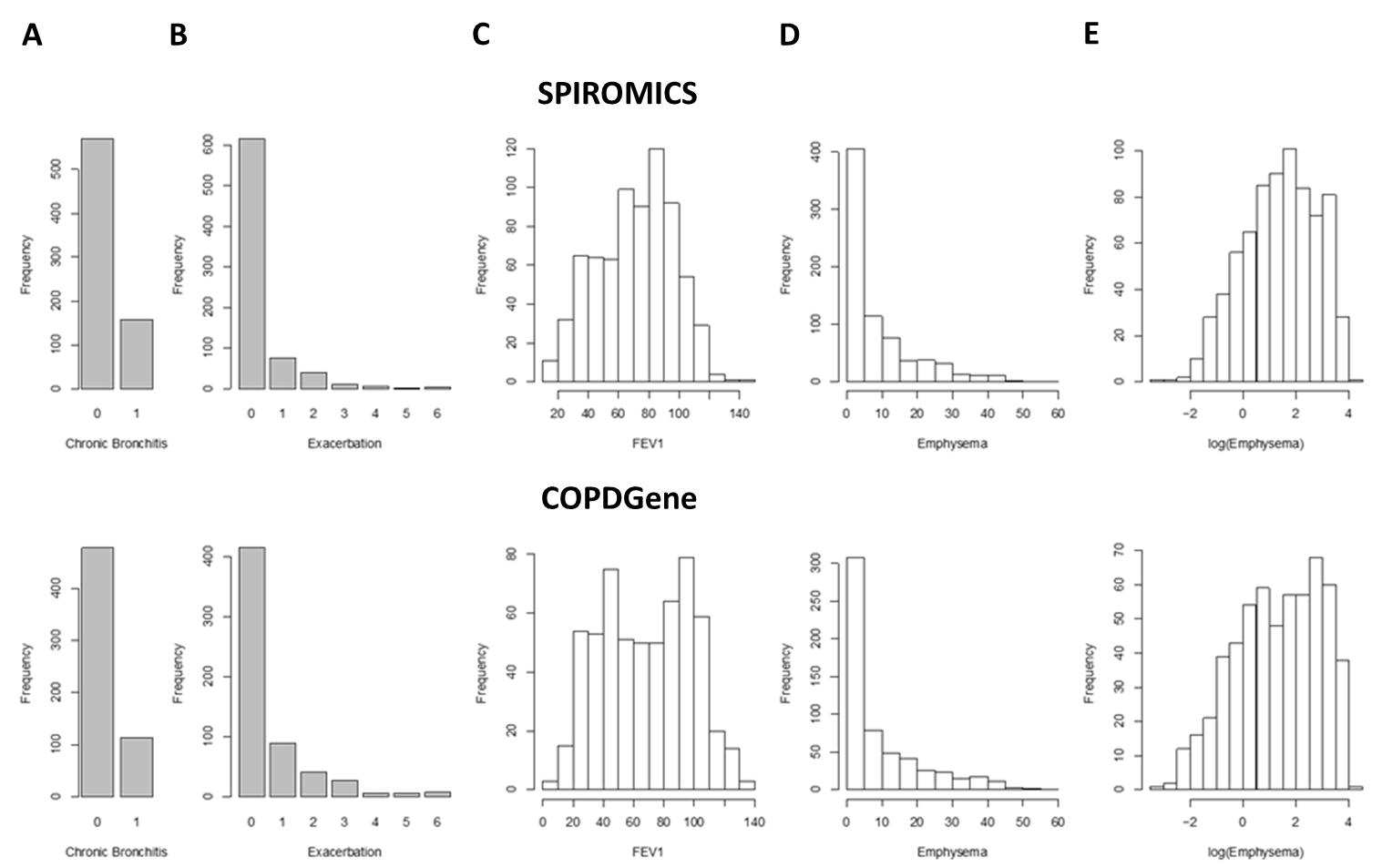 |
| --- |
| **S3 Fig.** Histograms demonstrating phenotype frequencies for SPIROMICS (top) and COPDGene (bottom) for (A) chronic bronchitis (0=no; 1=yes), (B) frequency of exacerbations in the 12 months prior to enrollment (exacerbations include respiratory events that required doctor visit, emergency room visit, hospitalization, or a change in antibiotic or steroid use, (C) FEV_1_% predicted, (D) percent total lung emphysema as defined by Hounsfield units -950; and (E) log transformation of percent emphysema. |
