# Supplementary material for: Common Genetic Polymorphisms Influence Blood Biomarker Measurements in COPD
Source: PLoS Genet. 2016 Aug 17;12(8):e1006011. doi: 10.1371/journal.pgen.1006011 (PMC4988780; doi:10.1371/journal.pgen.1006011)
Supplement: S4 Fig — LocusZoom plots show a 90 kb window (center) or a 500 kb window (right). On the Manhattan plots, the red line is the significant threshold after correction for multiple comparisons. For all LocusZoom plots, the top pQTL SNP is indicated. Red boxes in the LocusZoom plots show the location of the analyte gene (not all plots show this location but local pQTLs will have a box in one or both plots; for distant pQTLs, no red box will be present in either plot). Analyte gene location is shown with red arrow in Manhattan plots. (PDF) [file pgen.1006011.s012.pdf]

Supplemental Figure S3. Manhattan plots, q-q plots, and LocusZoom plots of pQTL findings for all analytes where significant pQTLs were identified. LocusZoom plots show a 90 kb window (center) or a 500 kb window (right). On the Manhattan plots, the red line is the significant threshold after correction for multiple comparisons. For all LocusZoom plots, the top pQTL SNP is indicated. Red boxes in the LocusZoom plots show the location of the analyte gene (not all plots show this location but local pQTLs will have a box in one or both plots; for distant pQTLs, no red box will be present in either plot). Analyte gene location is shown with red arrow in Manhattan plots.

Advanced glycosylation end product-specific receptor (RAGE) — **Gene Name: *AGER***

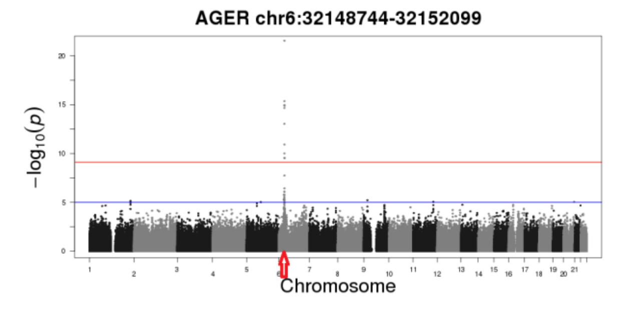

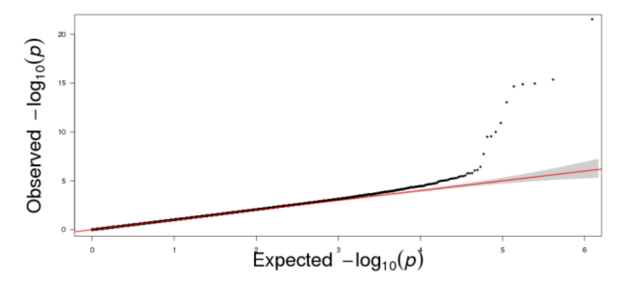

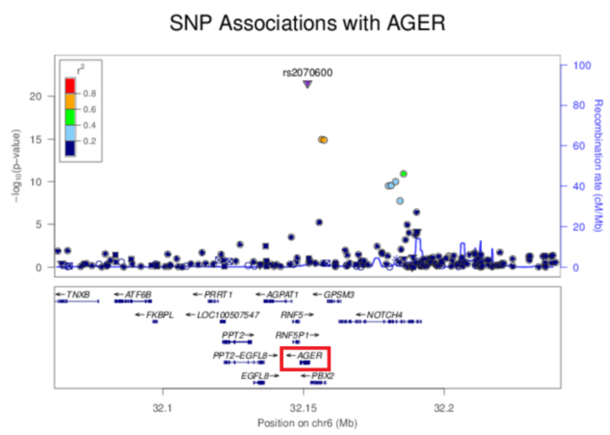

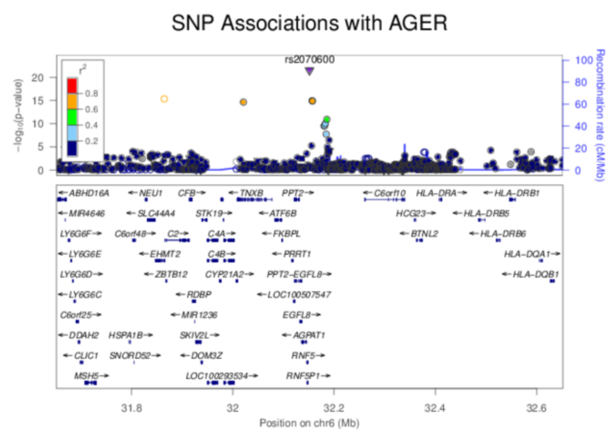

Apolipoprotein A-IV (Apo A-IV)	Gene Name: *APOA4*

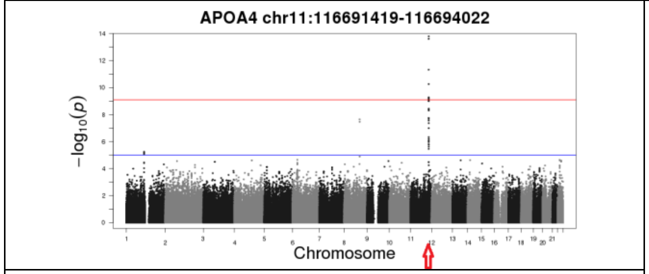

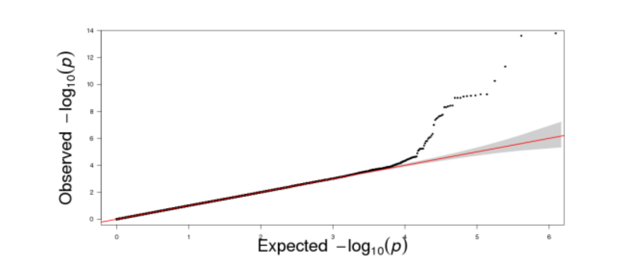

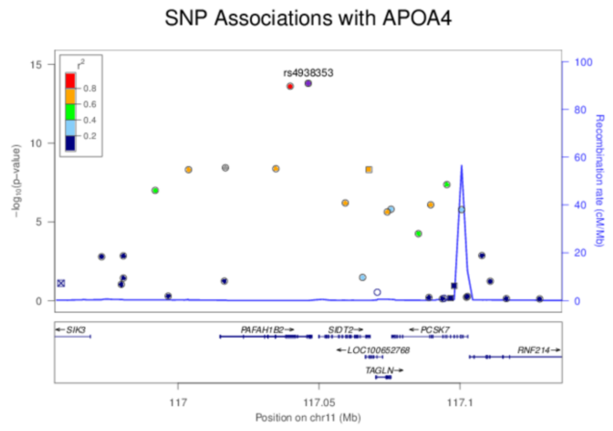

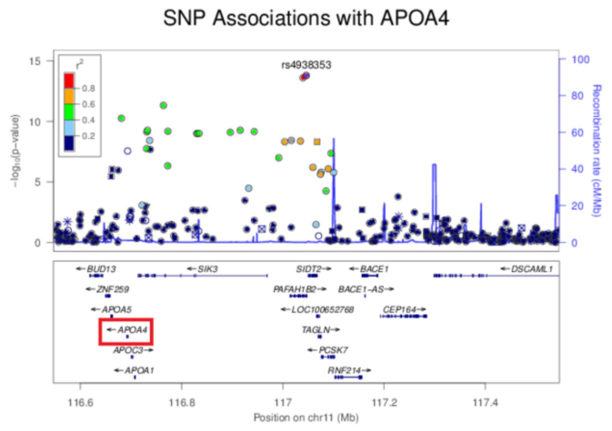

Complement Component 3	Gene Name: *C3*

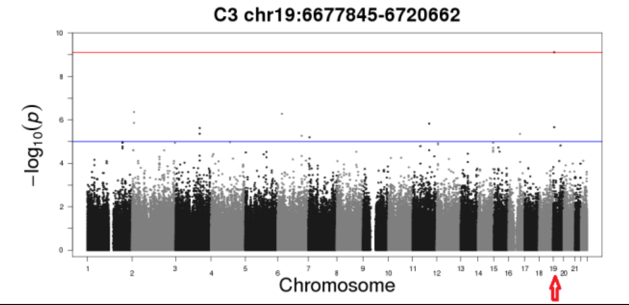

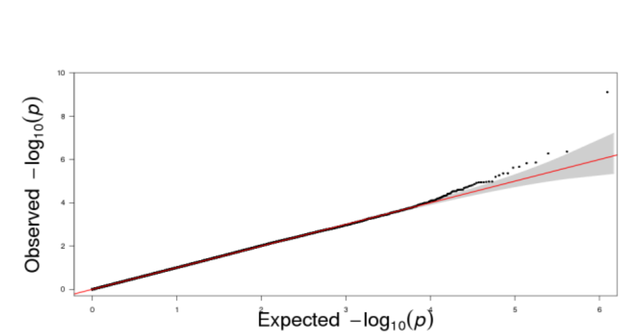

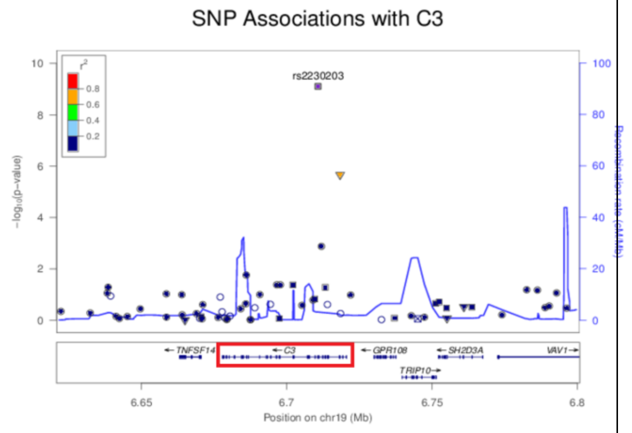

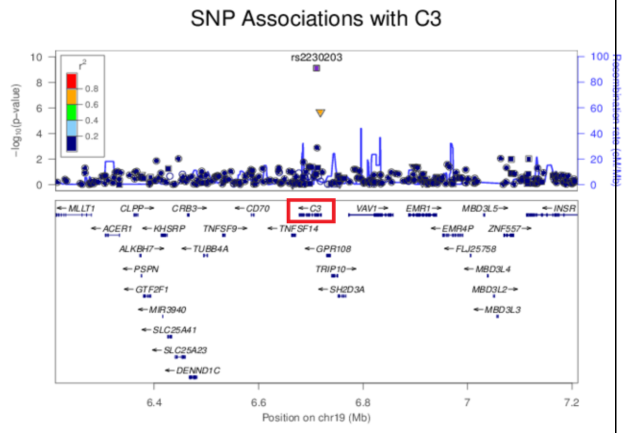

Chemokine (C-C motif) ligand 16 (pulmonary and activation-regulated) — **Gene Name: *CCL16***

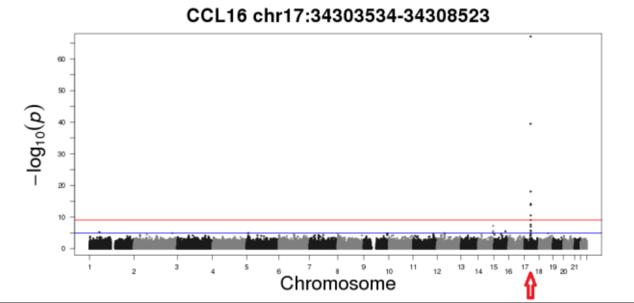

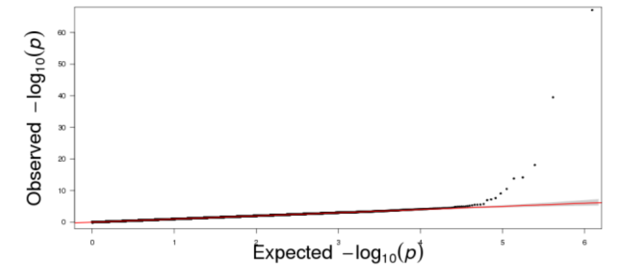

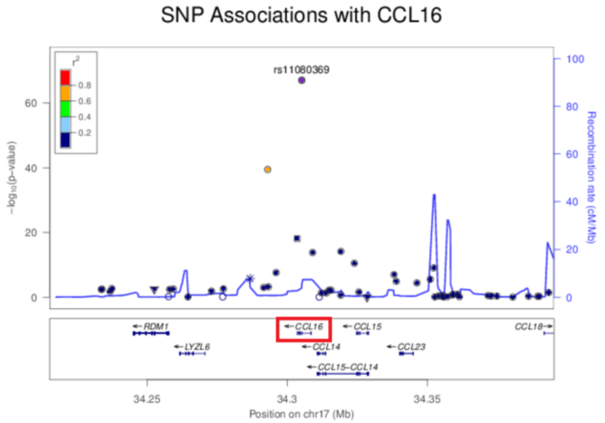

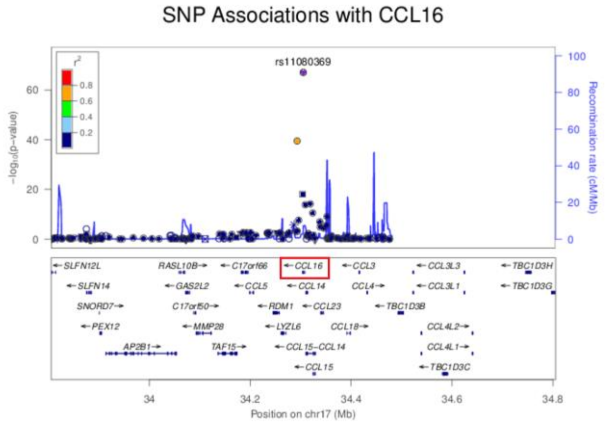

C-C motif chemokine 18 — **Gene Name: *CCL18***

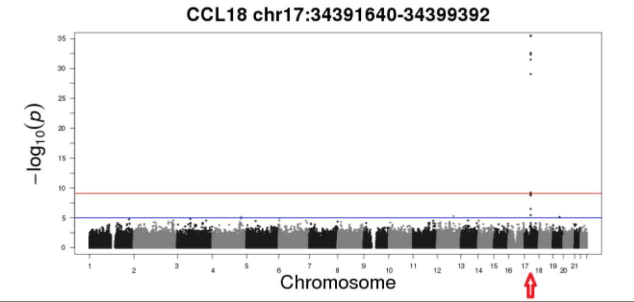

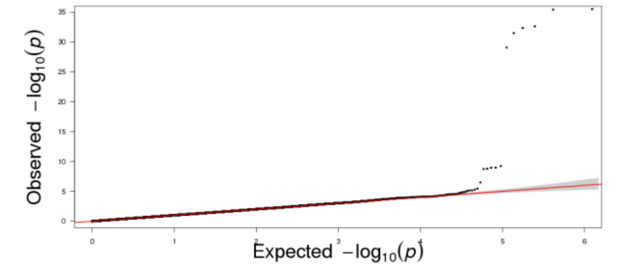

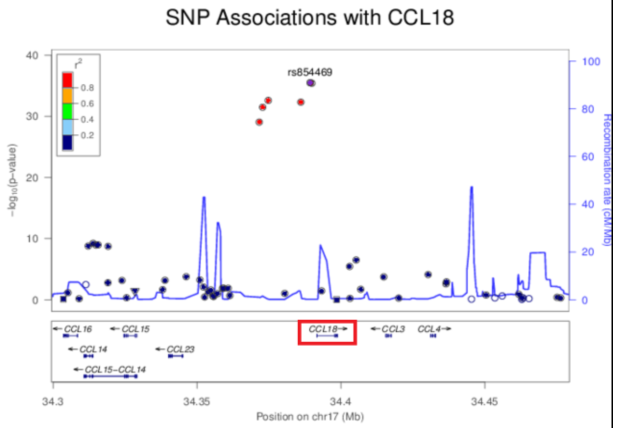

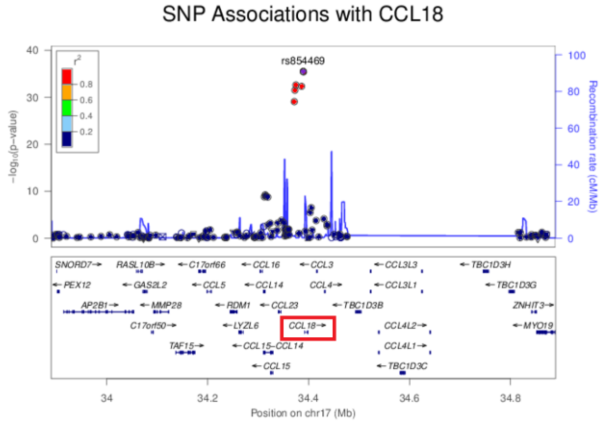

Chemokine (C-C motif) ligand 23 (Myeloid Progenitor Inhibitory Factor 1; MPIF-1) **Gene Name: *CCL23***

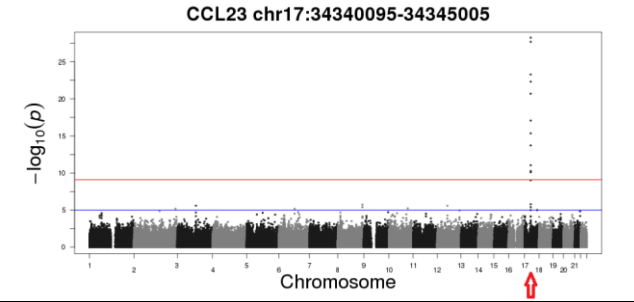

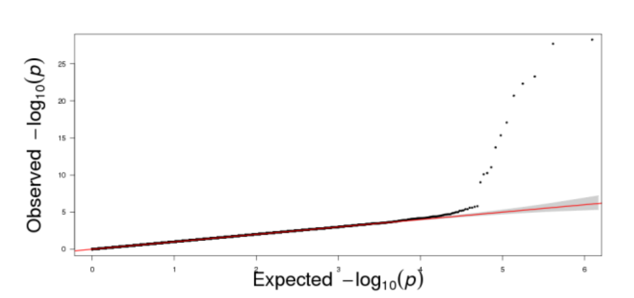

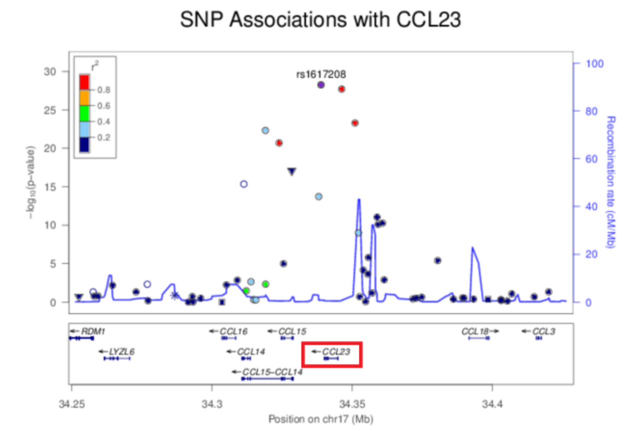

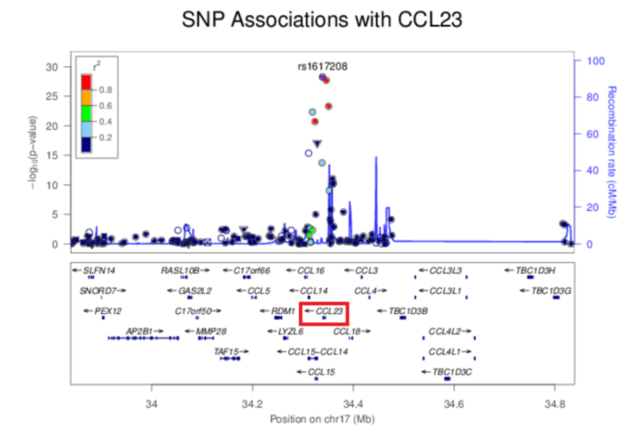

Chemokine (C-C motif) ligand 24 (Eotaxin-2) **Gene Name: *CCL24***

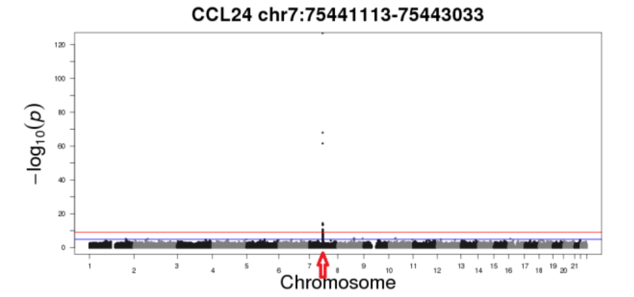

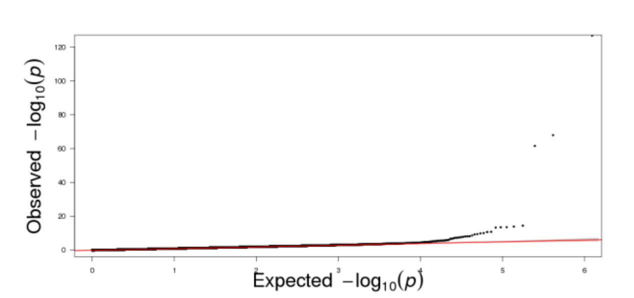

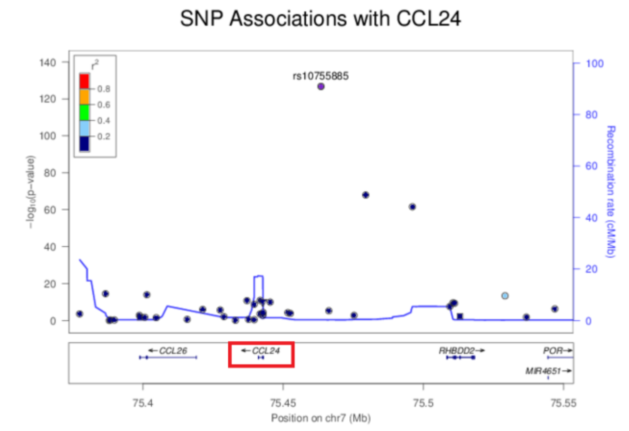

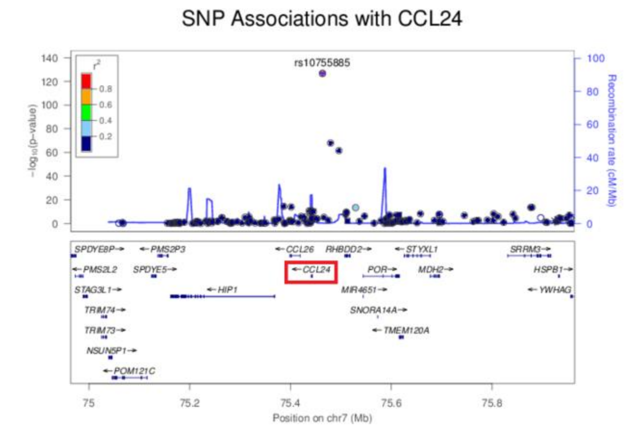

Chemokine (C-C motif) ligand 4 (Macrophage Inflammatory Protein-1 β; MIP-1 β) — **Gene Name: *CCL4***

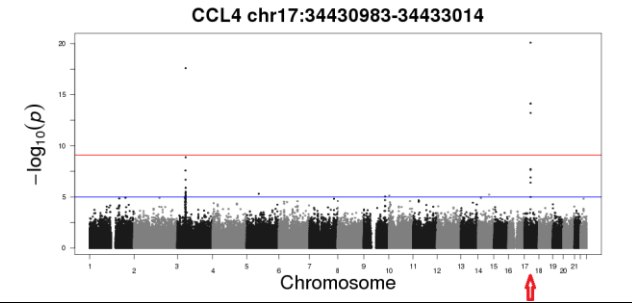

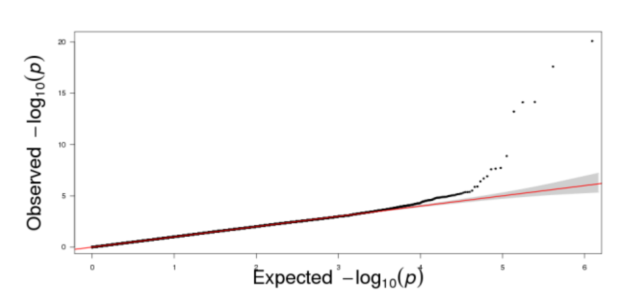

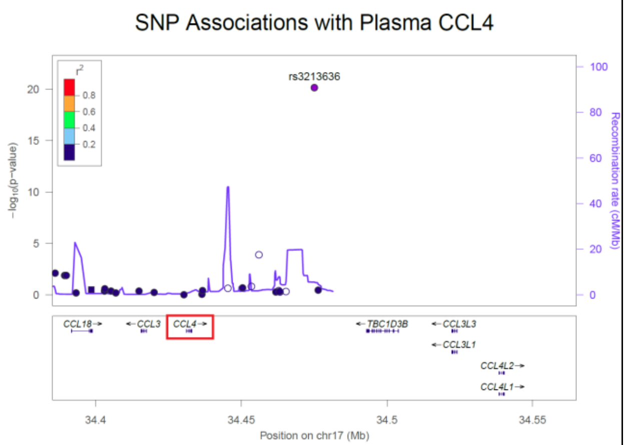

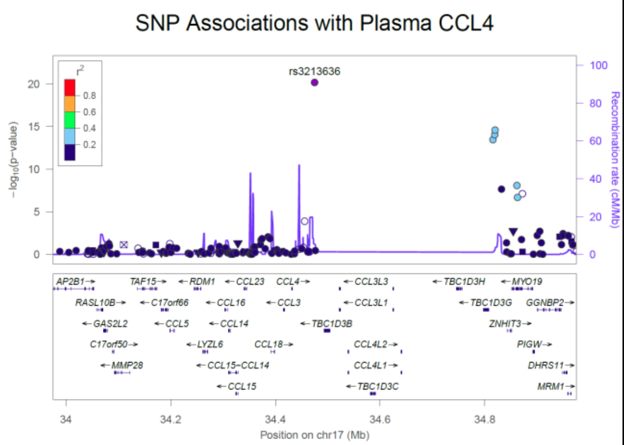

Chemokine (C-C motif) ligand 8 (Monocyte Chemotactic Protein 2; MCP-2) — **Gene Name: *CCL8***

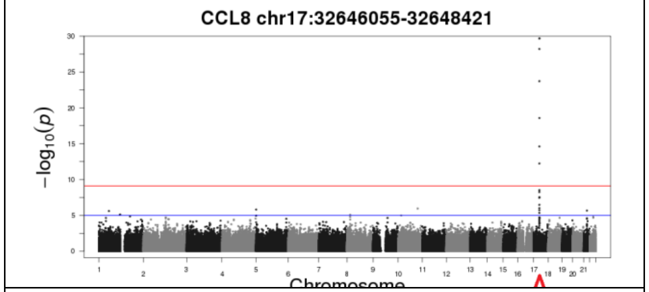

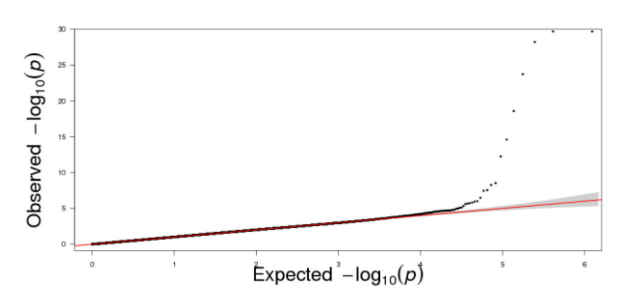

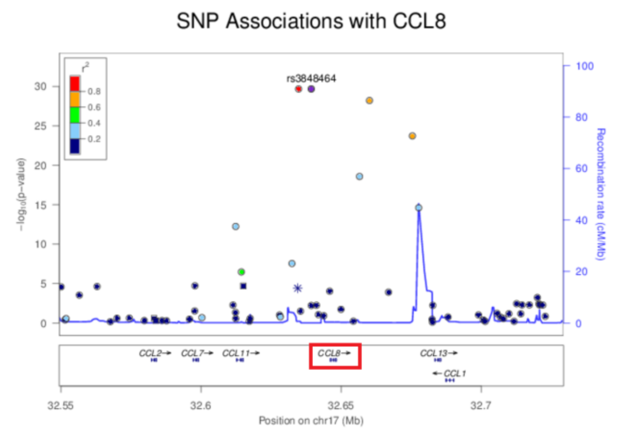

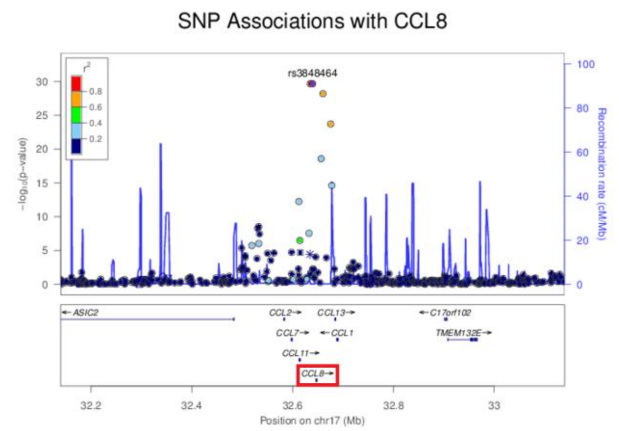

Cadherin-1 [E-cadherin (epithelial)] — Gene Name: *CDH1*

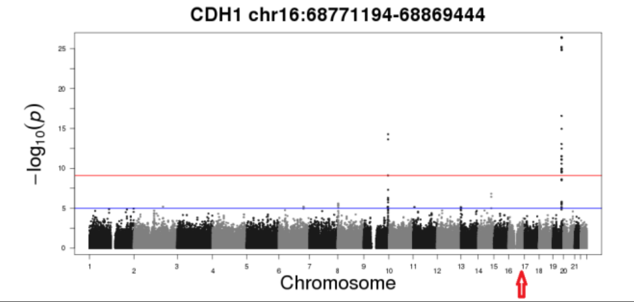

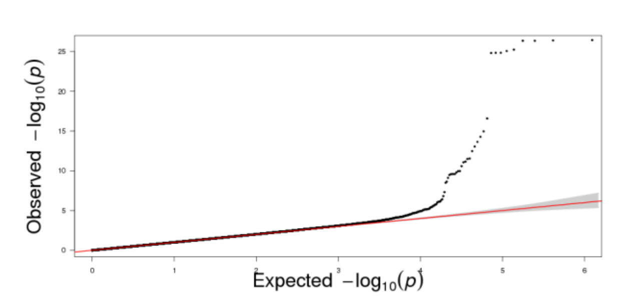

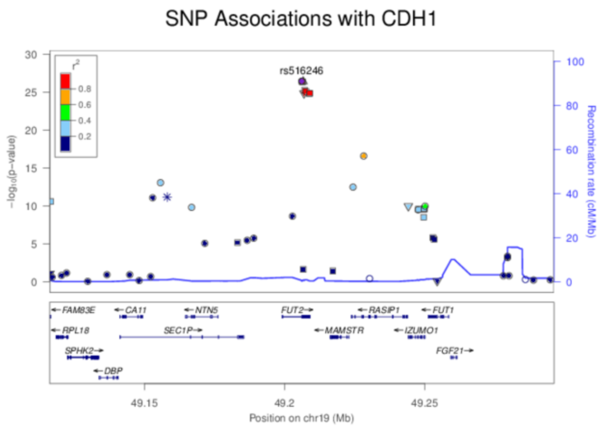

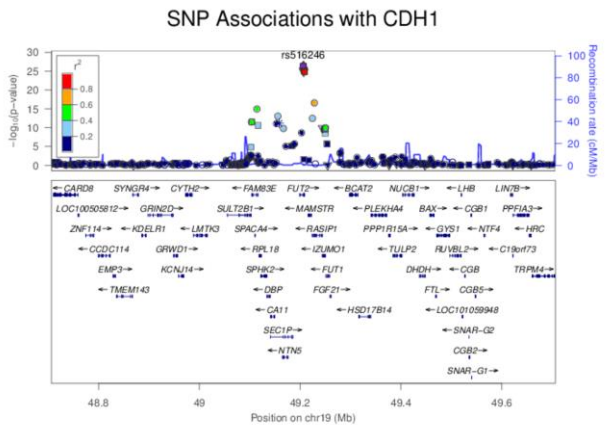

Cystatin-B — Gene Name: *CSTB*

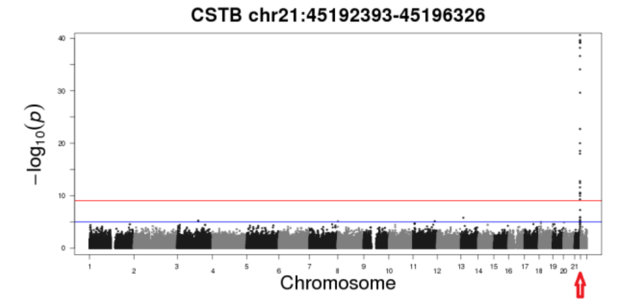

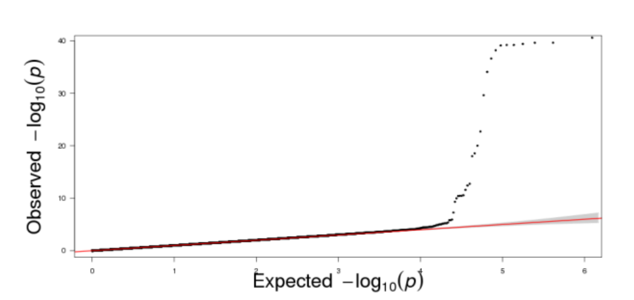

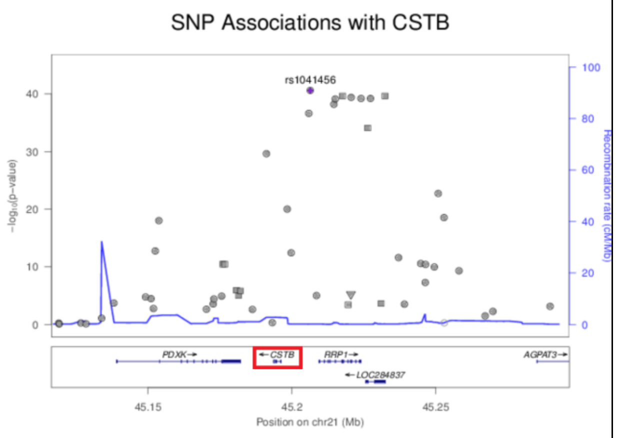

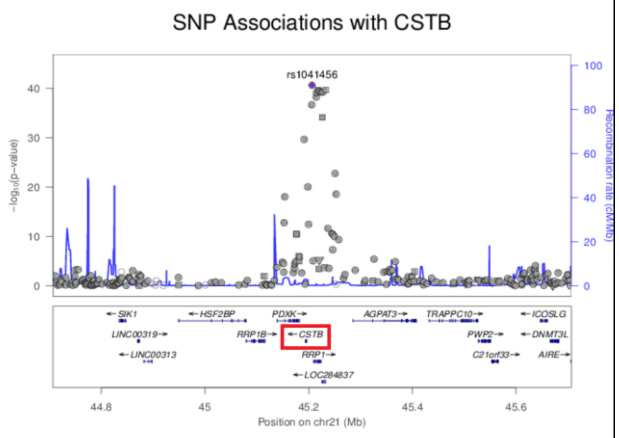

C-X-C motif chemokine 5 (Epithelial-Derived Neutrophil-Activating Protein 78; ENA-78)

Gene Name: *CXCL5*

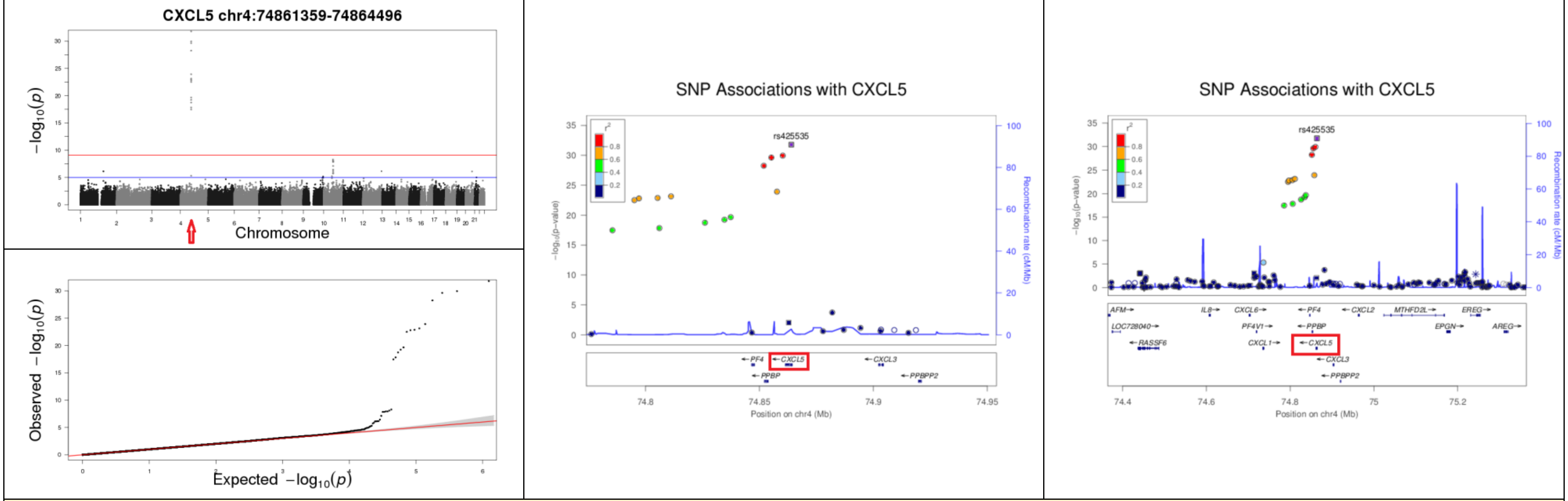

Coagulation Factor VII

Gene Name: *F7*

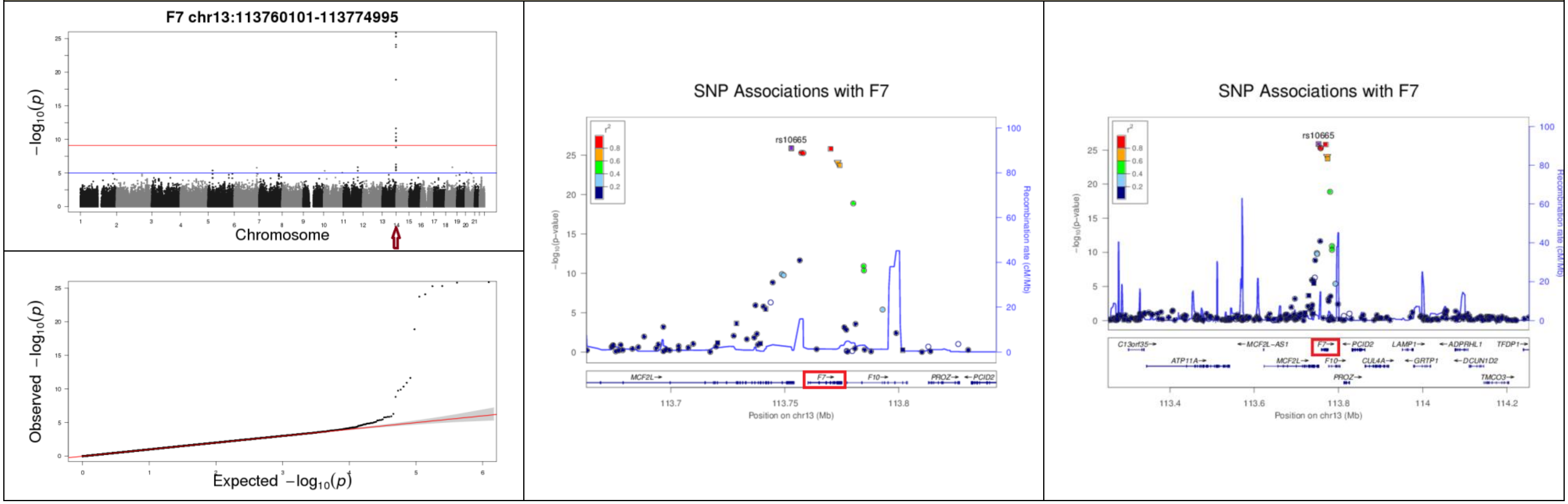

**Tumor necrosis factor receptor superfamily member 6 (FASLG Receptor; CD95)**	**Gene Name: *FAS***

FAS chr10:90750414-90775542

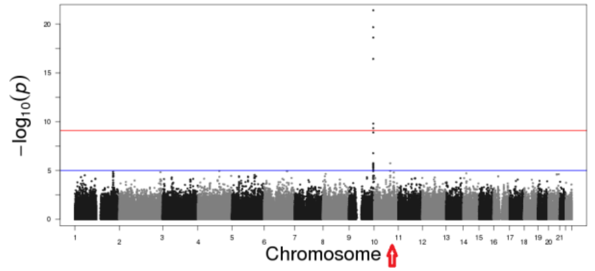

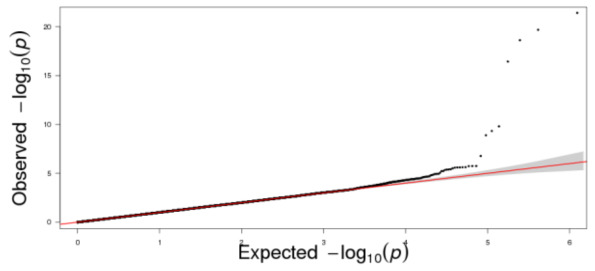

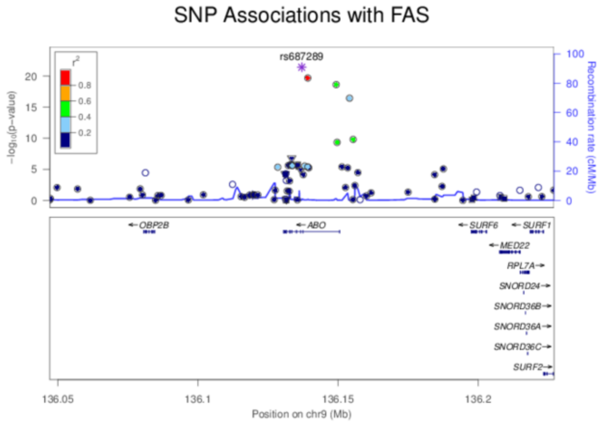

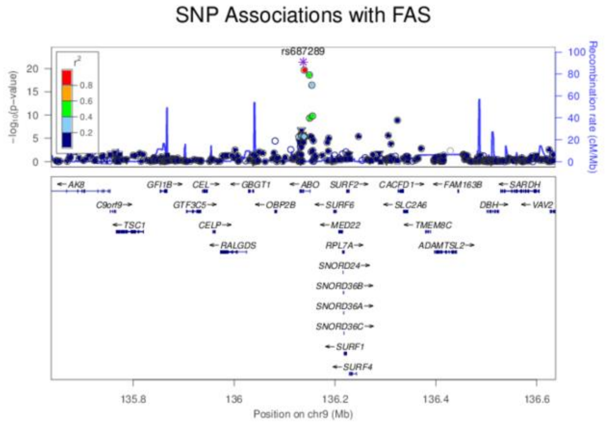

**Vitamin D-Binding Protein**	**Gene Name: *GC***

GC chr4:72607410-72669758

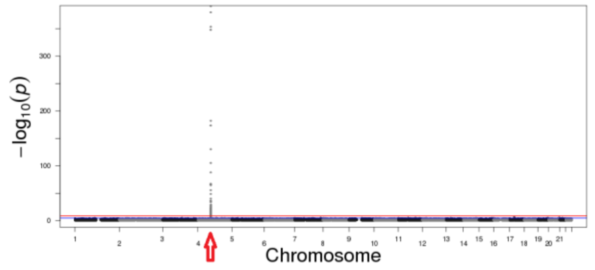

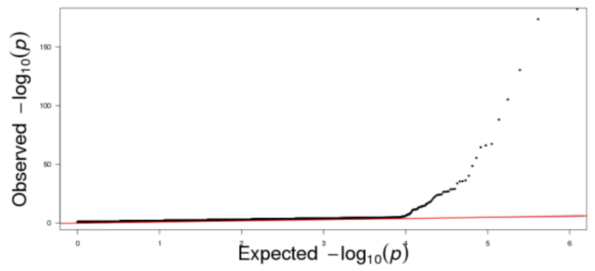

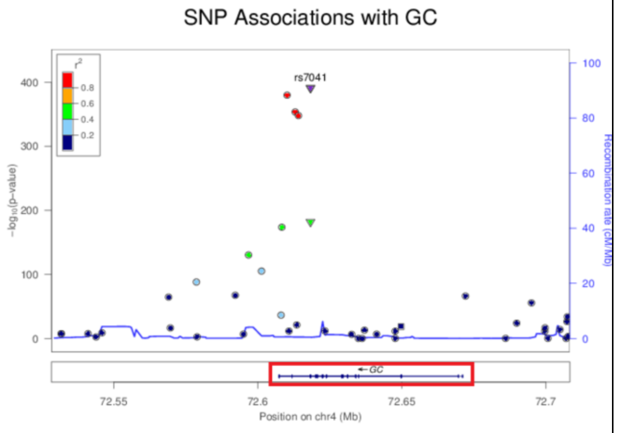

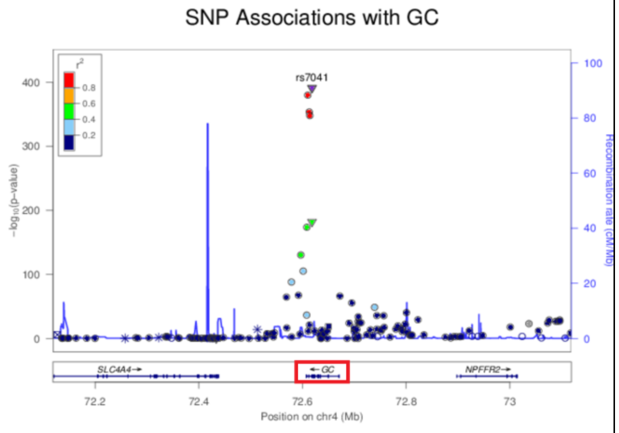

Hepatocyte Growth Factor

Gene Name: ***HGF***

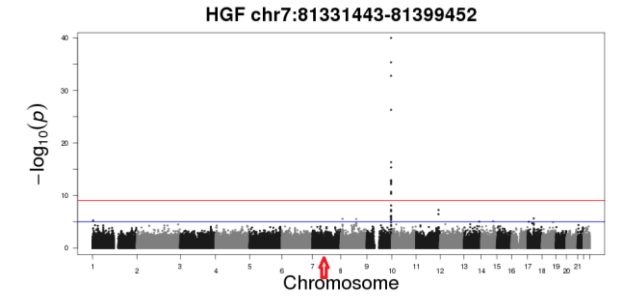

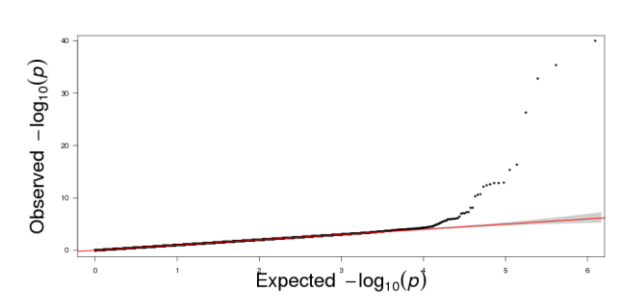

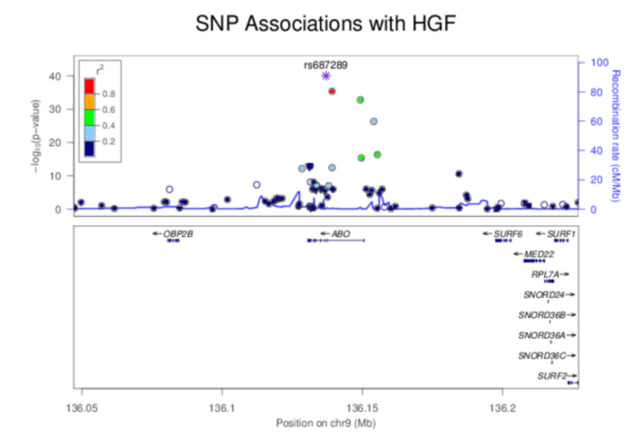

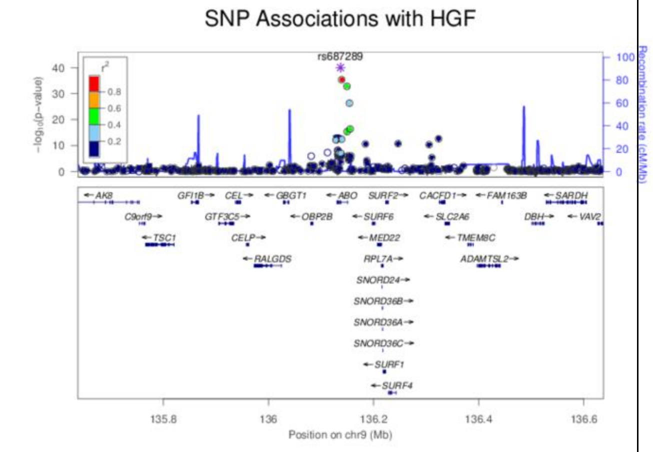

Haptoglobin

Gene Name: ***HP***

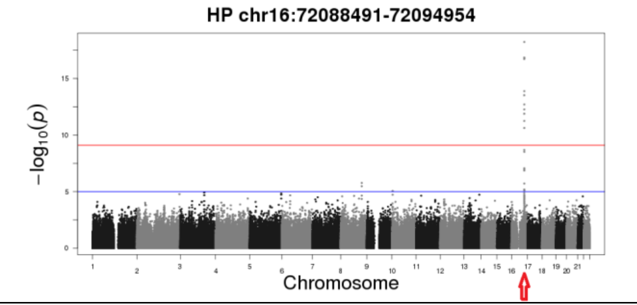

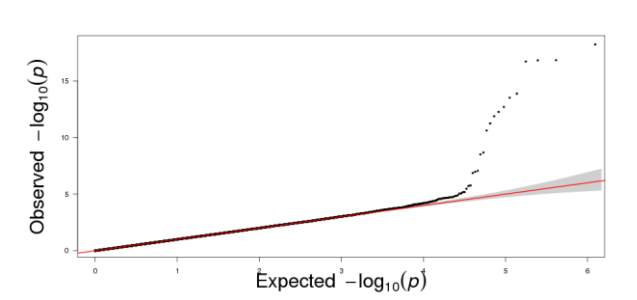

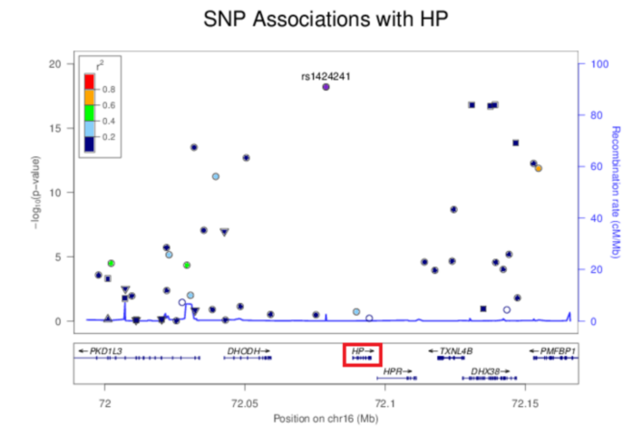

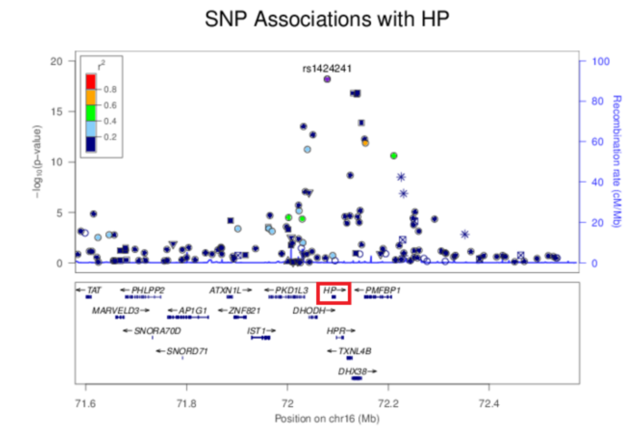

Interleukin-12 Subunit p40 (IL-12p40) — Gene Name: *IL12B*

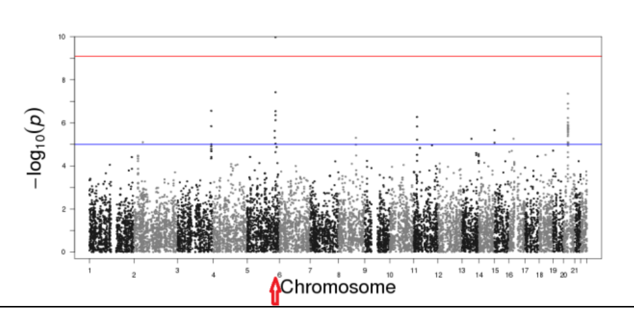

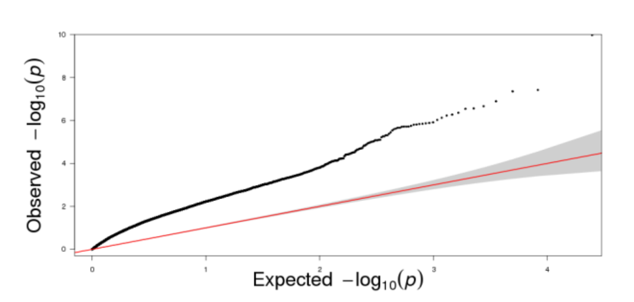

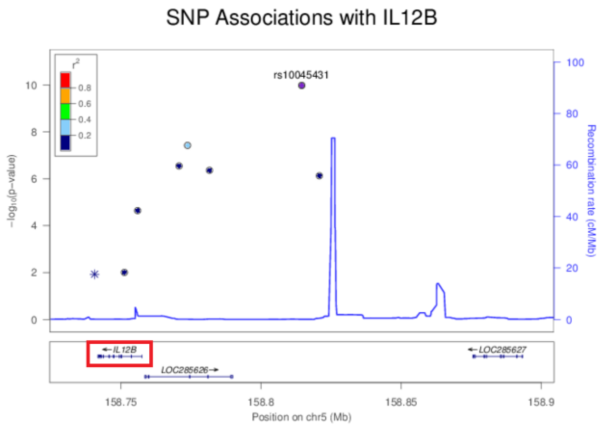

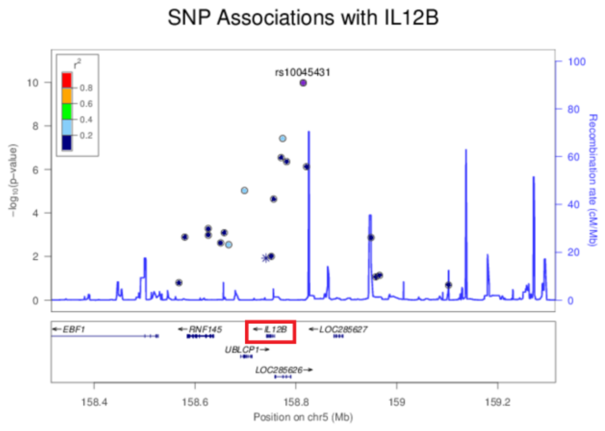

Interleukin-16 — Gene Name: *IL16*

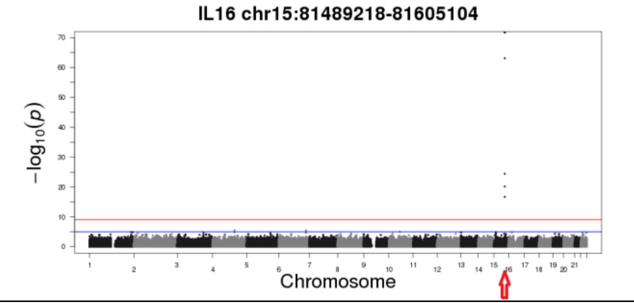

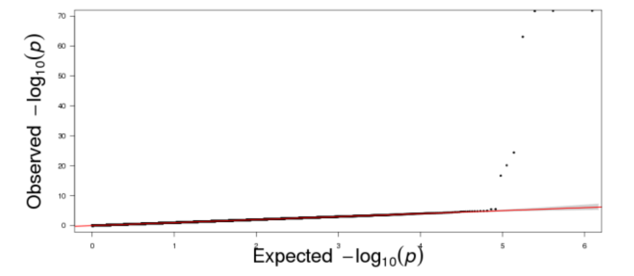

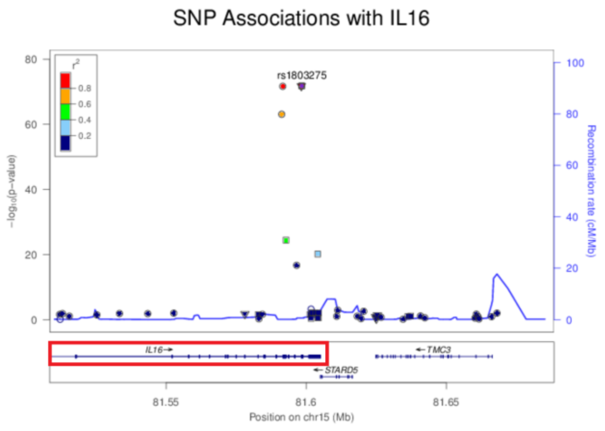

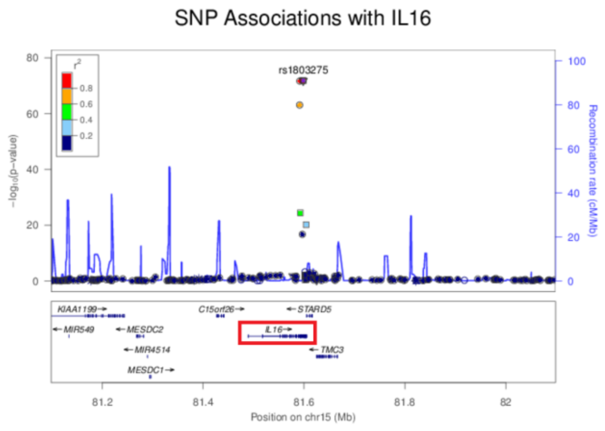

Interleukin-18 — Gene Name: *IL18*

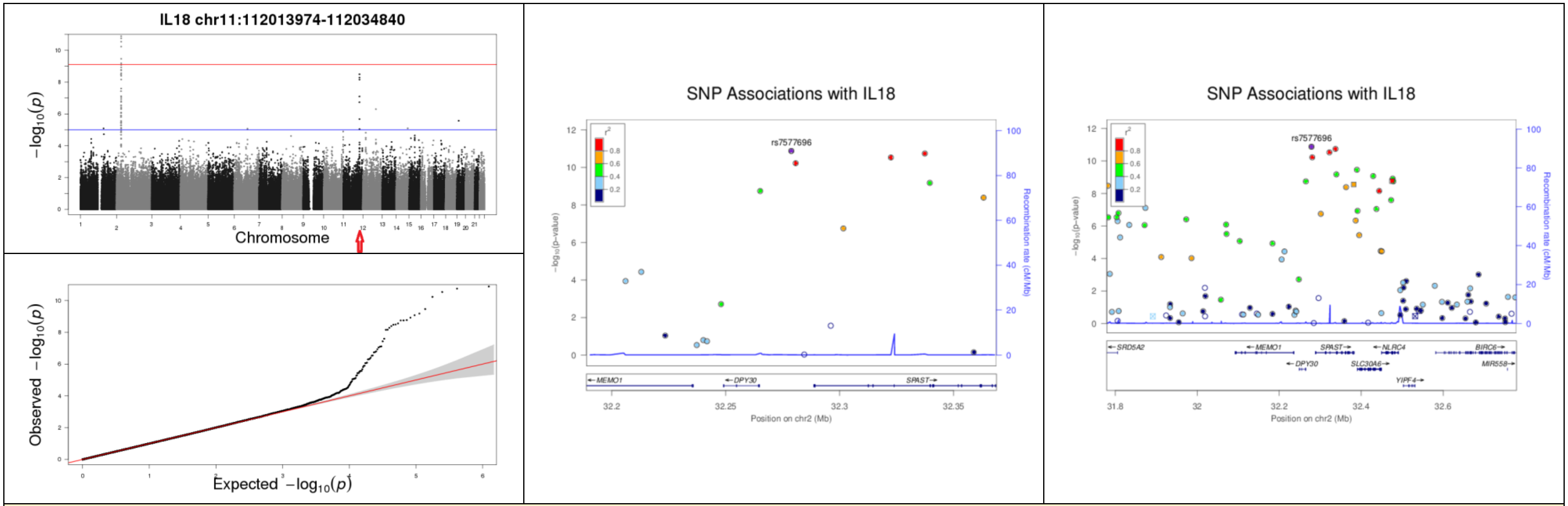

Interleukin-23A **Gene Name: *IL23A***

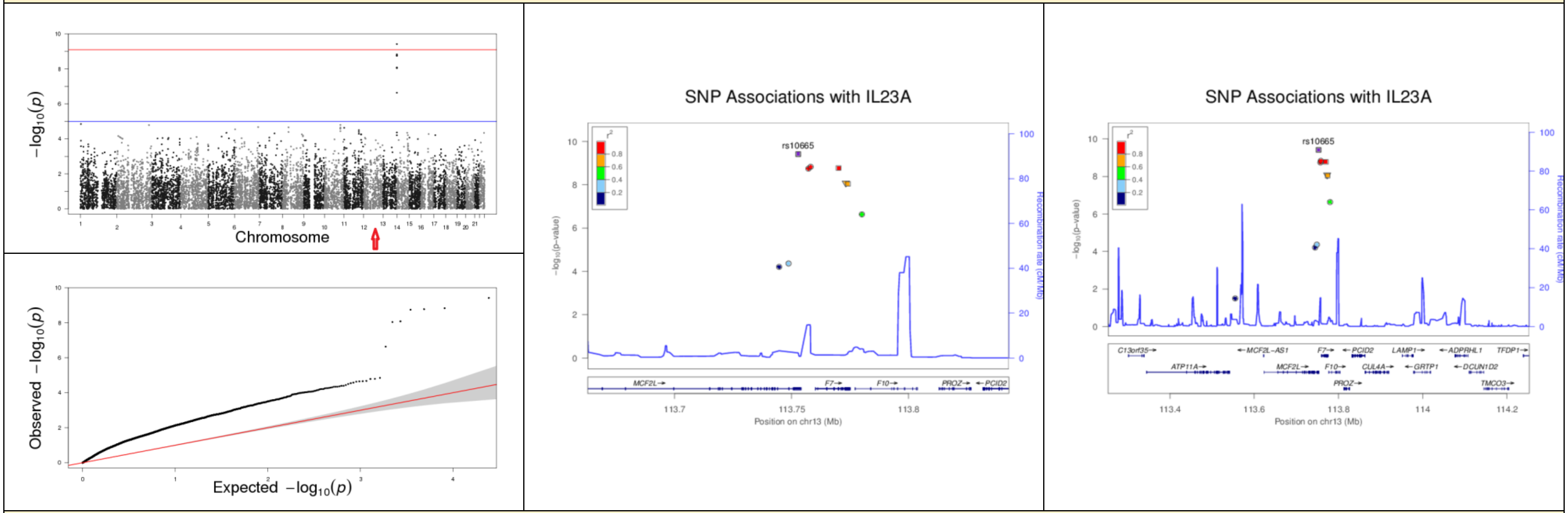

Interleukin-2 receptor subunit alpha **Gene Name: *IL2RA***

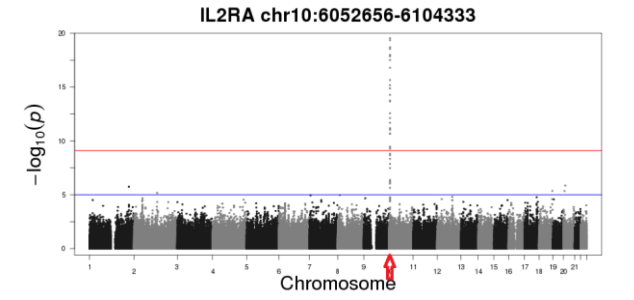

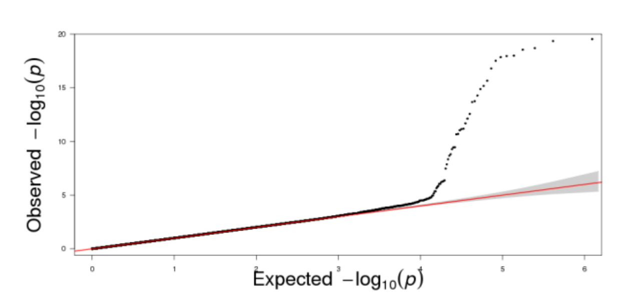

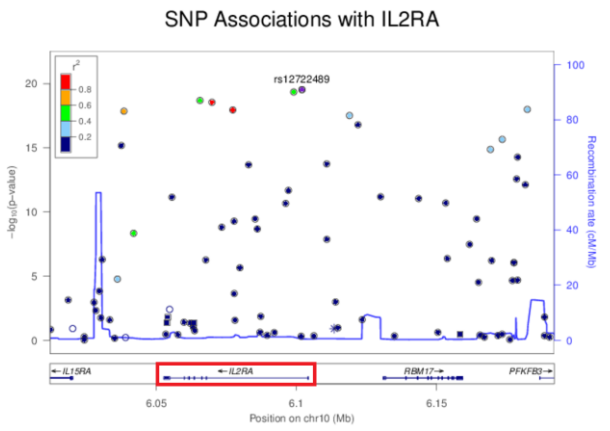

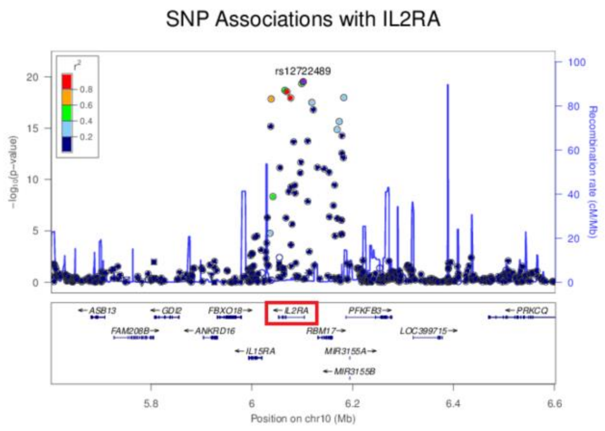

Interleukin-6 receptor subunit alpha **Gene Name: *IL6R***

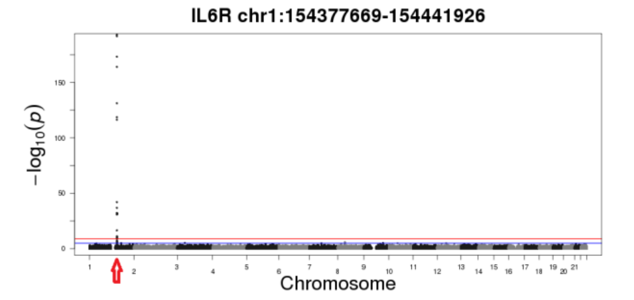

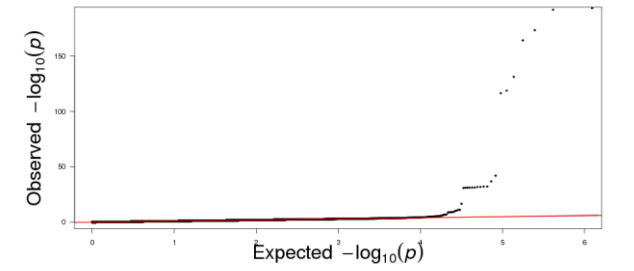

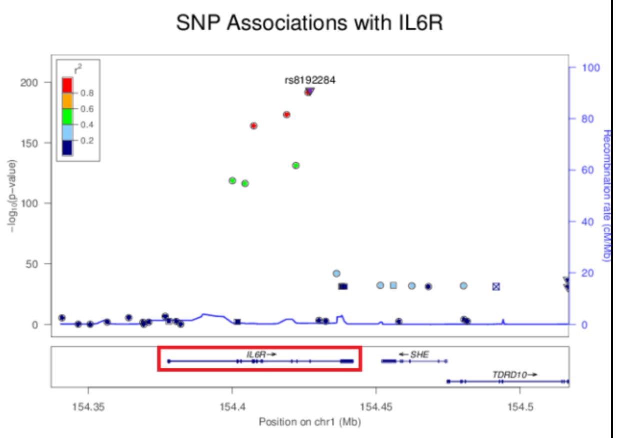

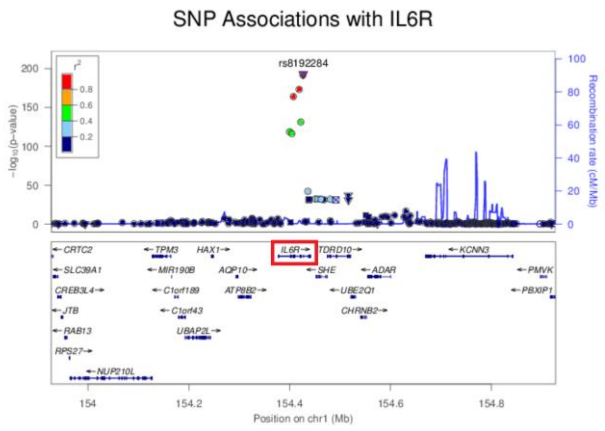

Apolipoprotein(a) **Gene Name: *LPA***

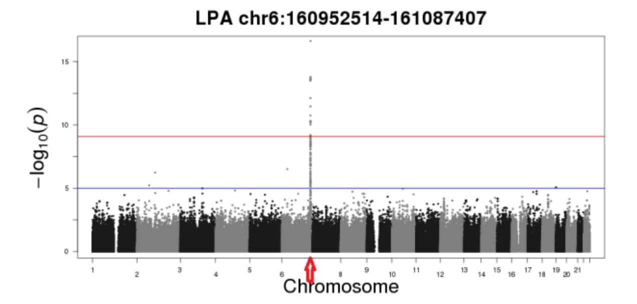

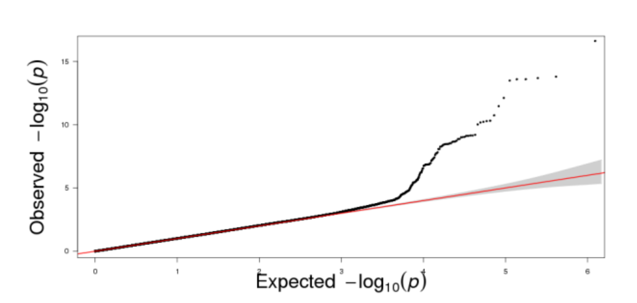

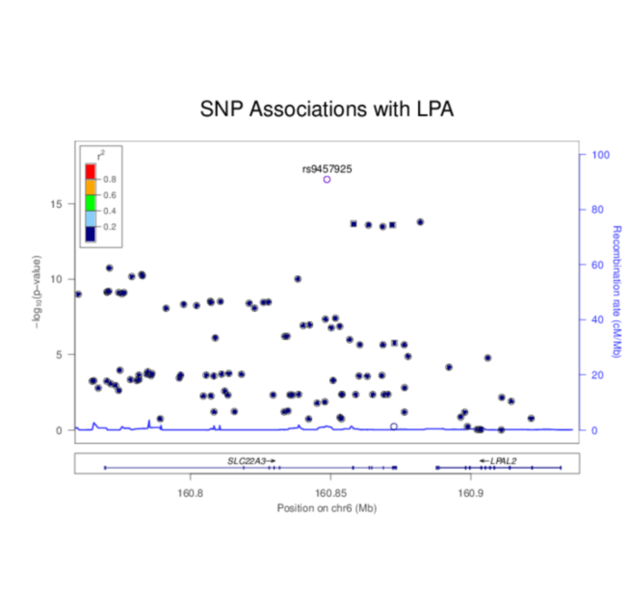

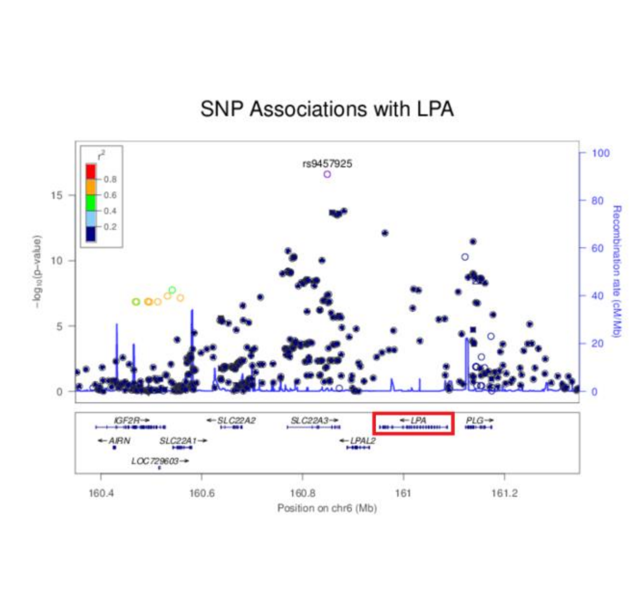

Lactotransferrin — Gene Name: *LTF*

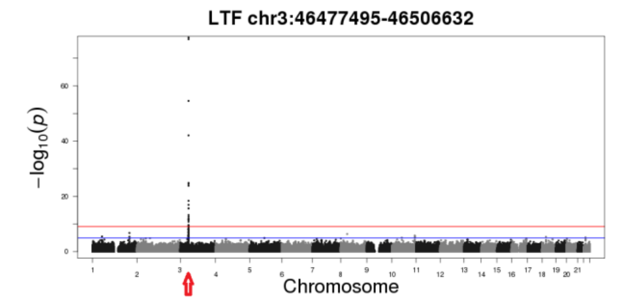

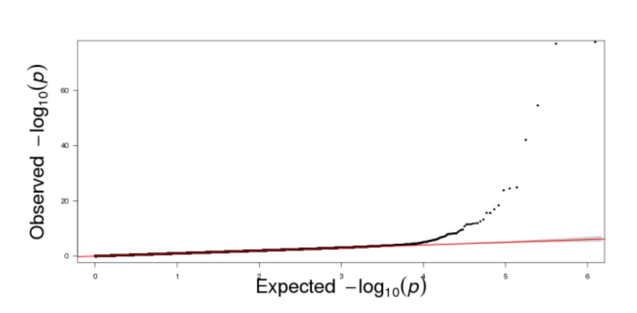

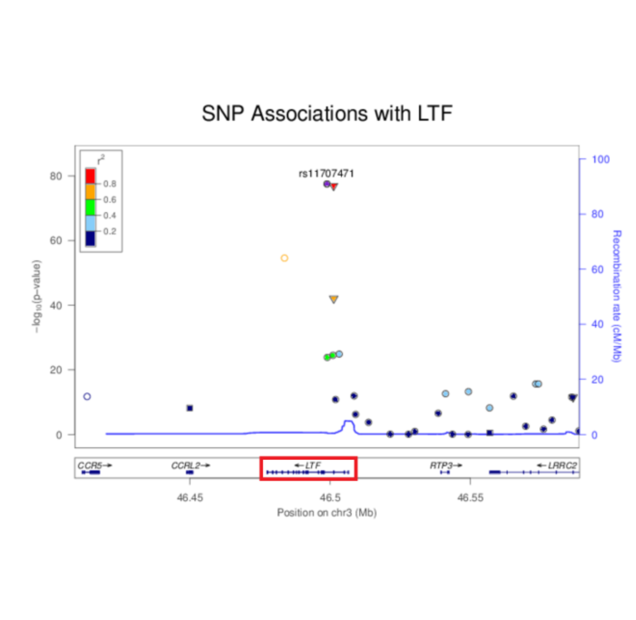

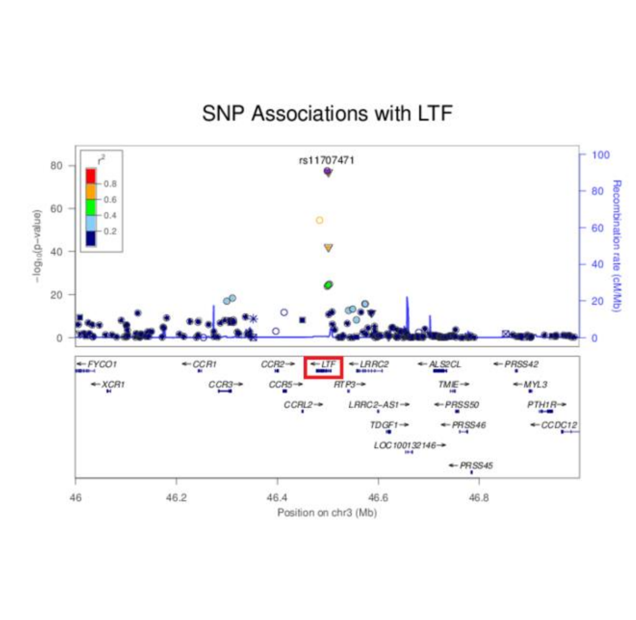

MHC class I polypeptide-related sequence A — Gene Name: *MICA*

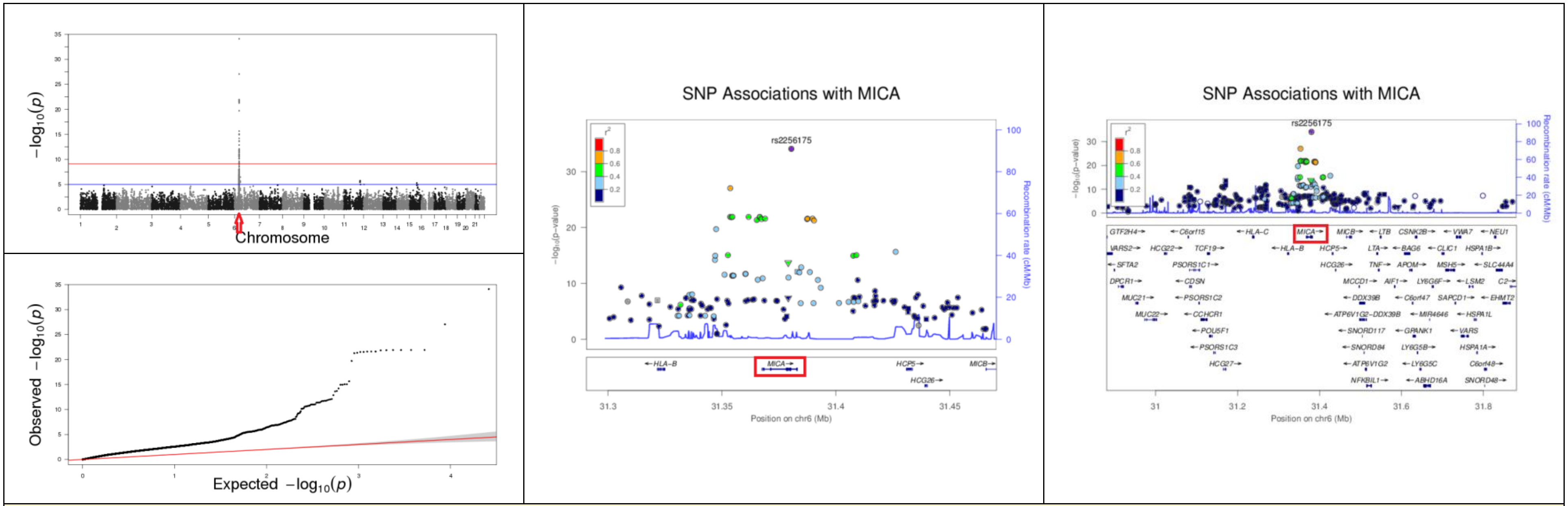

Stromelysin-1 (Matrix Metalloproteinase-3; MMP-3)

Gene Name: *MMP3*

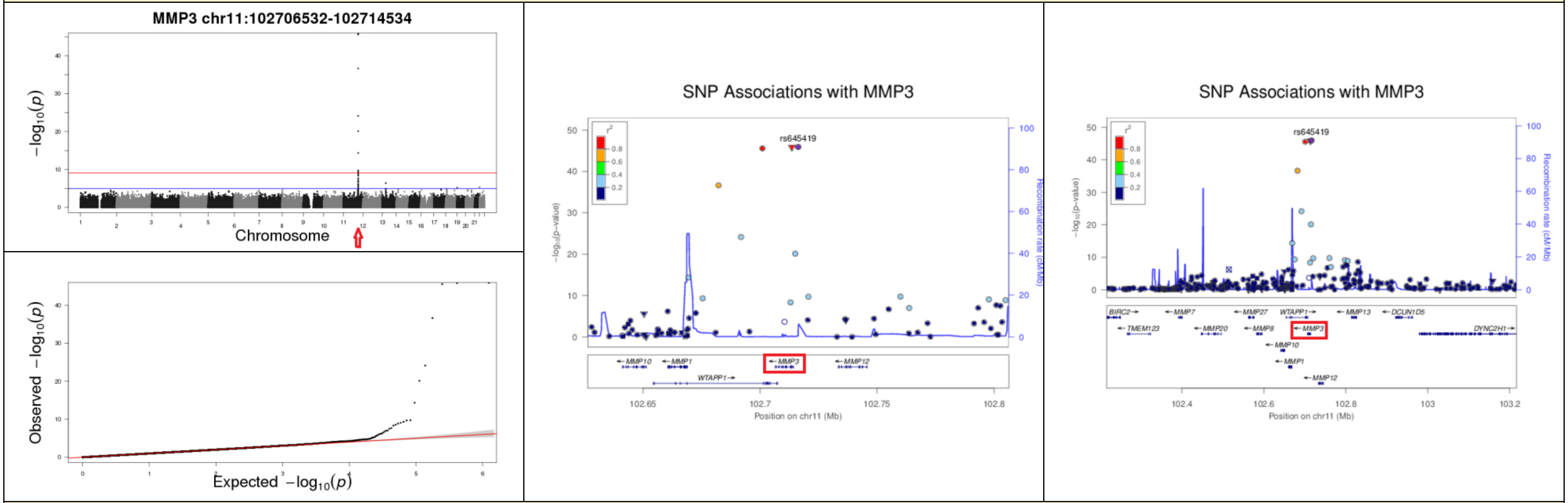

Neuronal Cell Adhesion Molecule

Gene Name: *NRCAM*

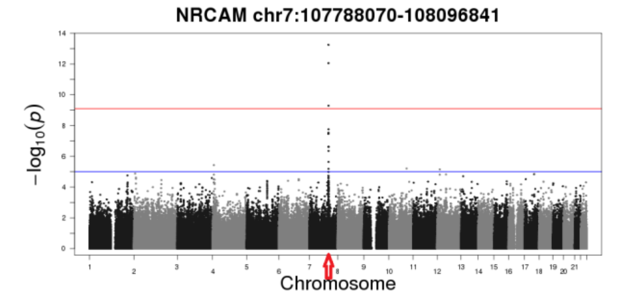

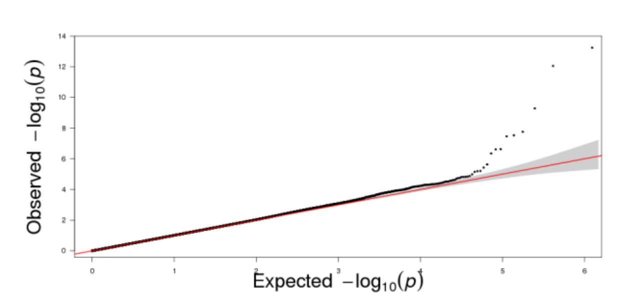

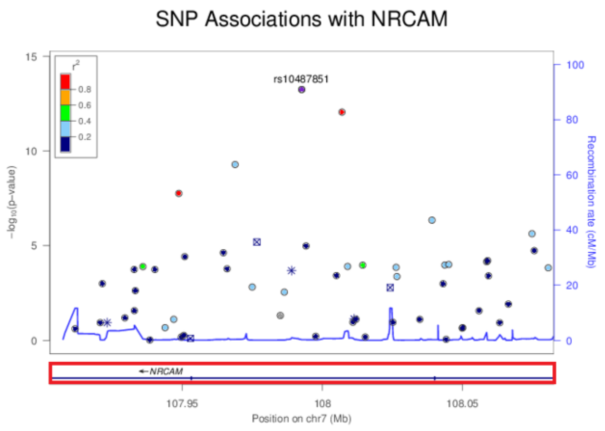

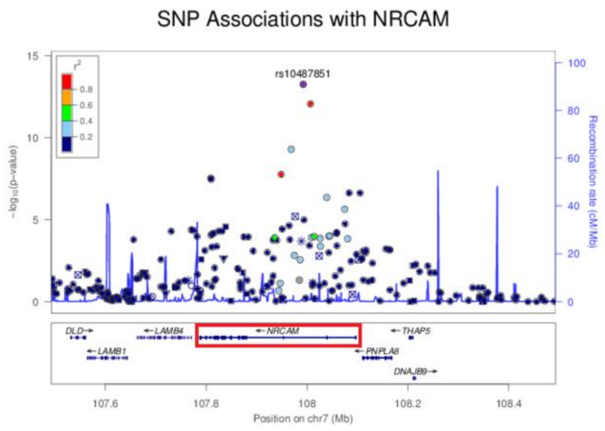

Platelet endothelial cell adhesion molecule **Gene Name: *PECAM1***

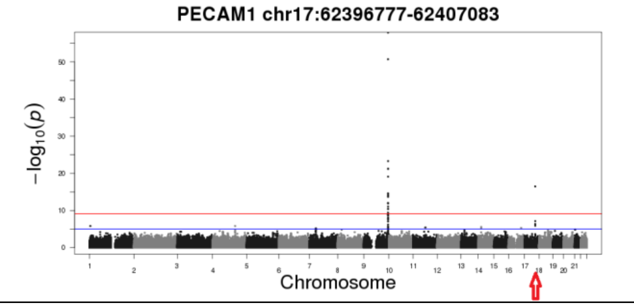

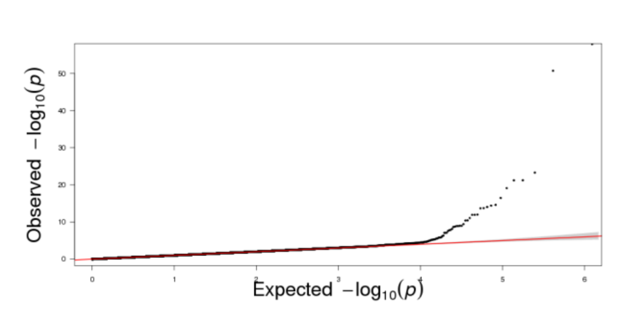

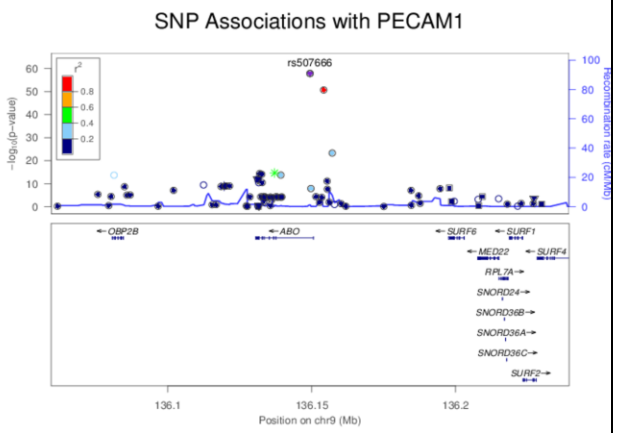

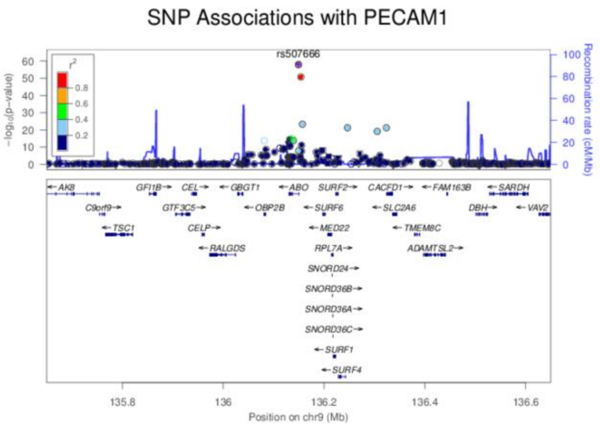

E-selectin **Gene Name: *SELE***

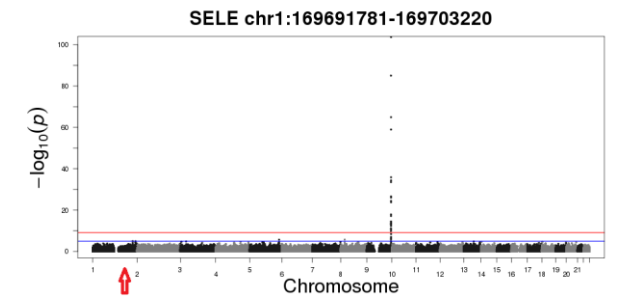

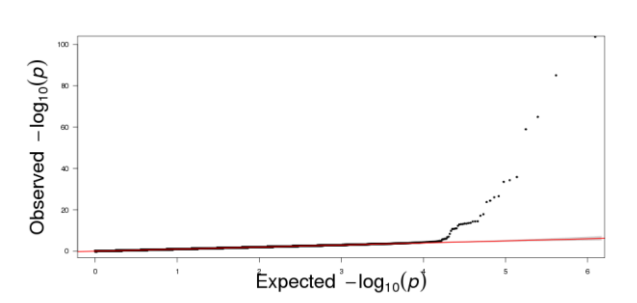

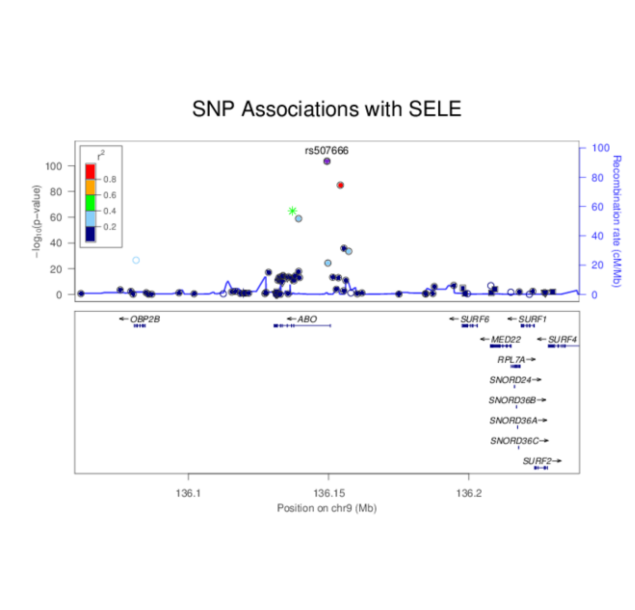

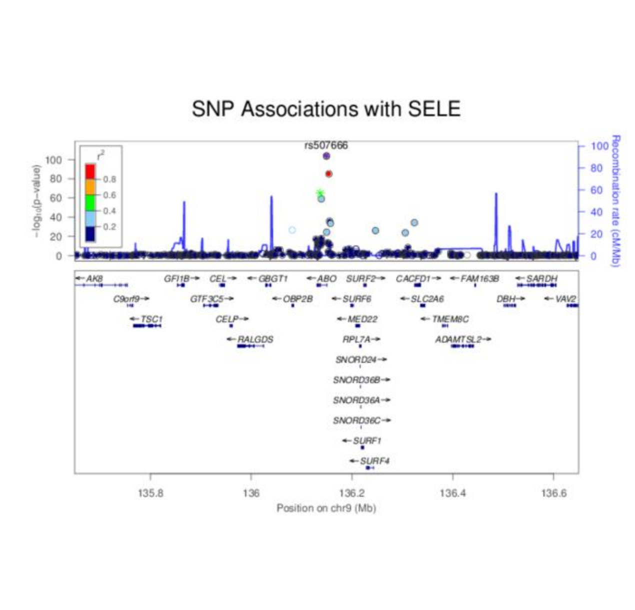

Alpha-1-Antitrypsin | **Gene Name: *SERPINA1***

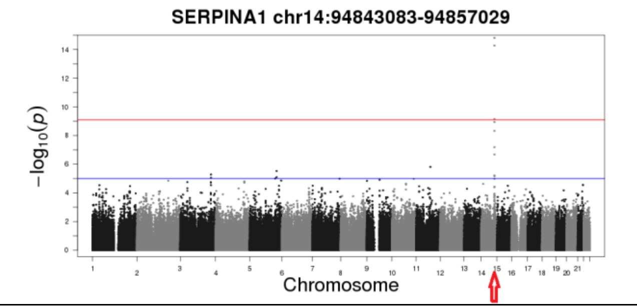

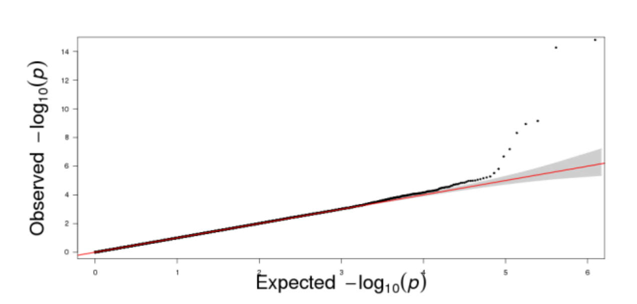

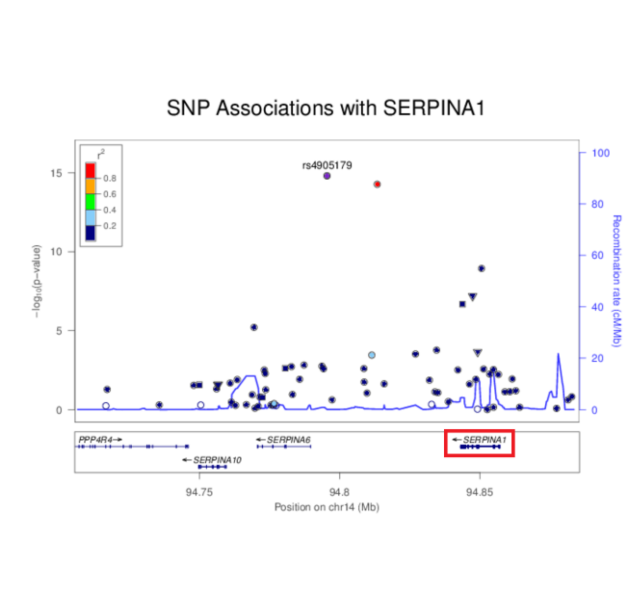

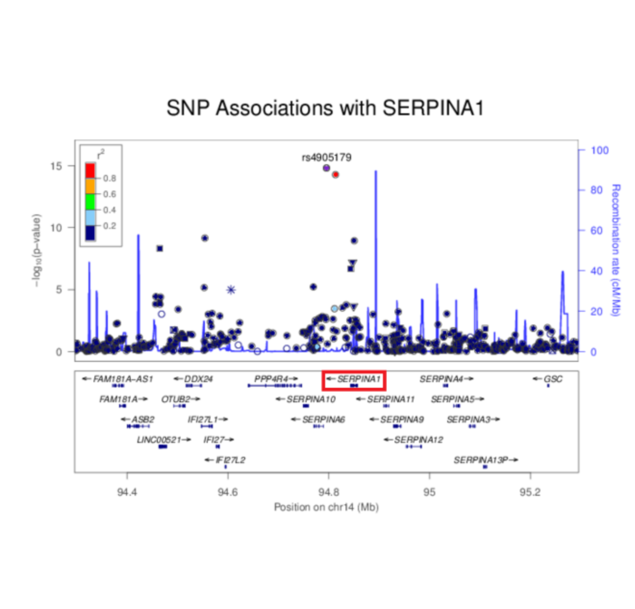

Pulmonary surfactant-associated protein D (SP-D) | **Gene Name: *SFTPD***

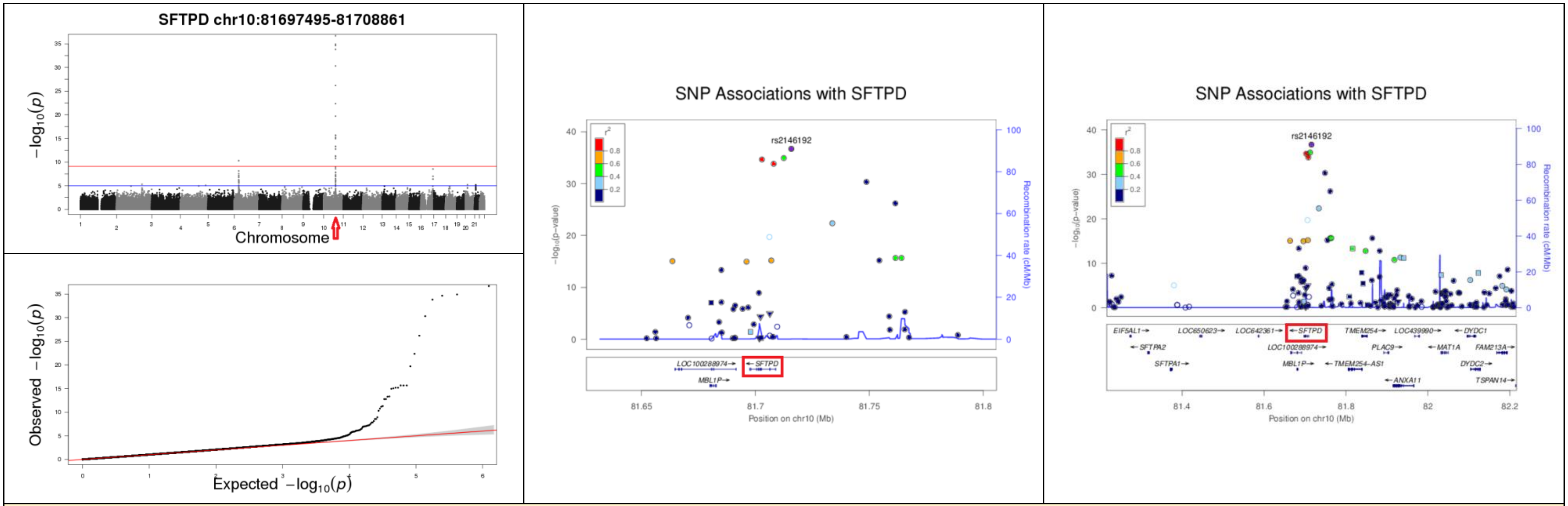

Sex Hormone-Binding Globulin **Gene Name: *SHBG***

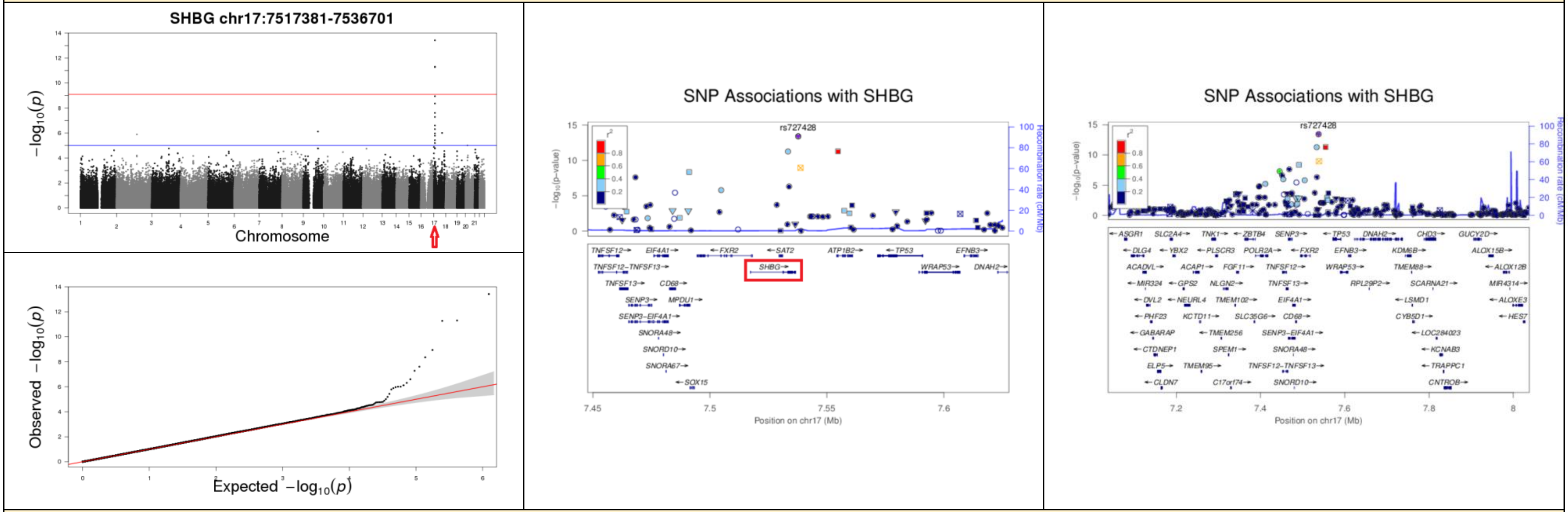

Sortilin **Gene Name: *SORT1***

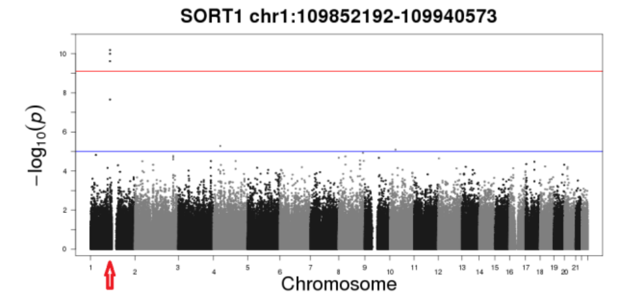

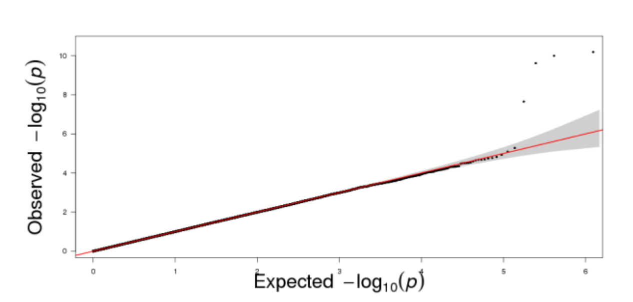

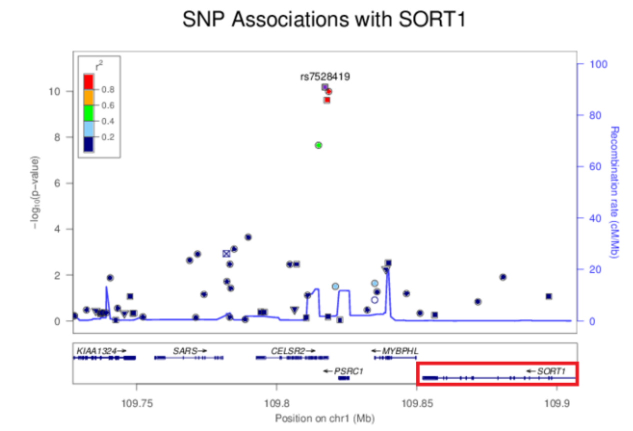

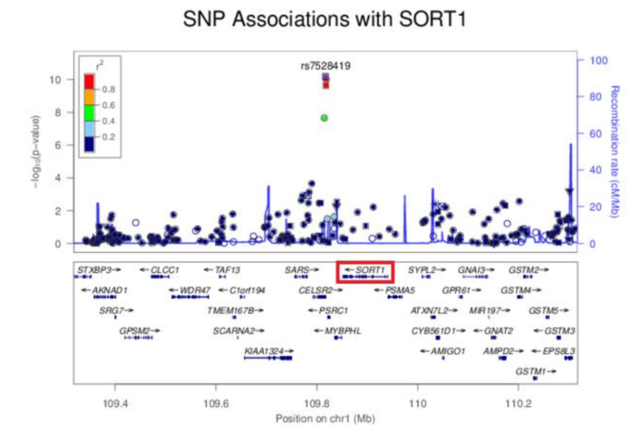

Pancreatic secretory trypsin inhibitor (TATI) **Gene Name: *SPINK1***

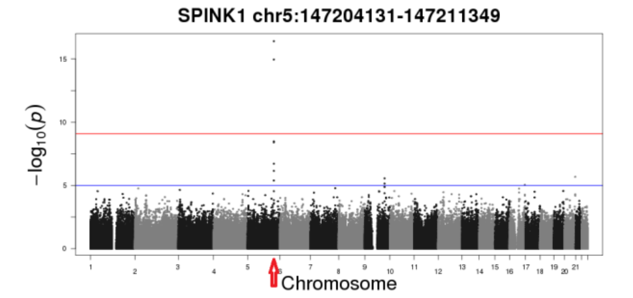

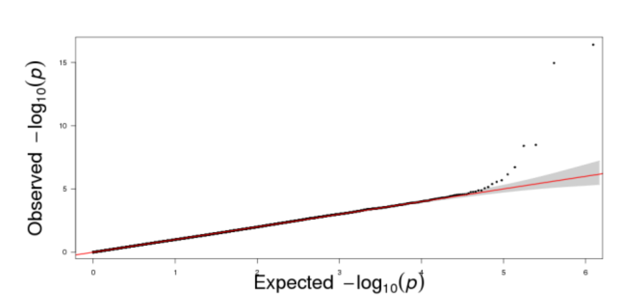

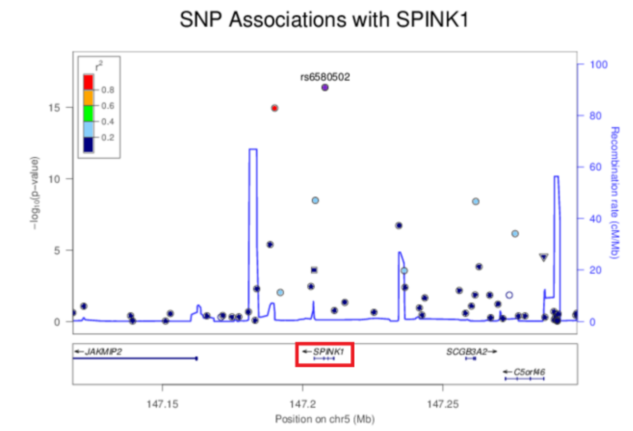

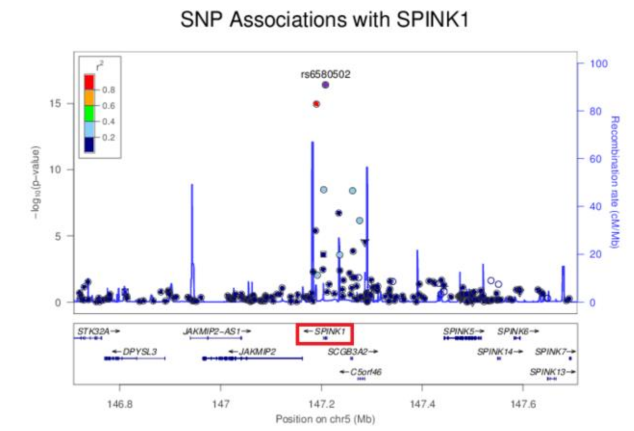

Tumor necrosis factor receptor superfamily member 10C (TNF-Related Apoptosis-inducing Ligand Receptor 3; TRAIL-R3) **Gene Name: *TNFRSF10C***

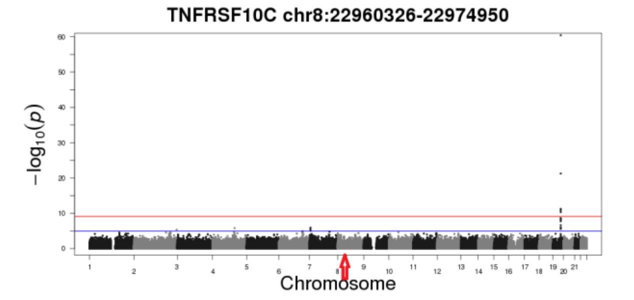

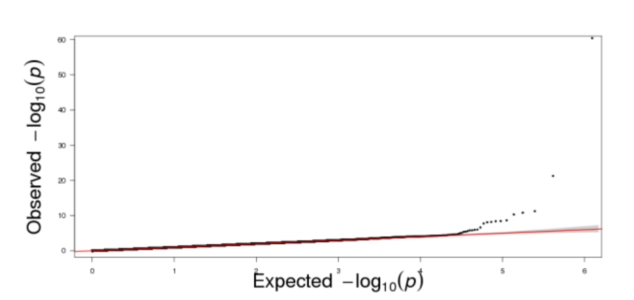

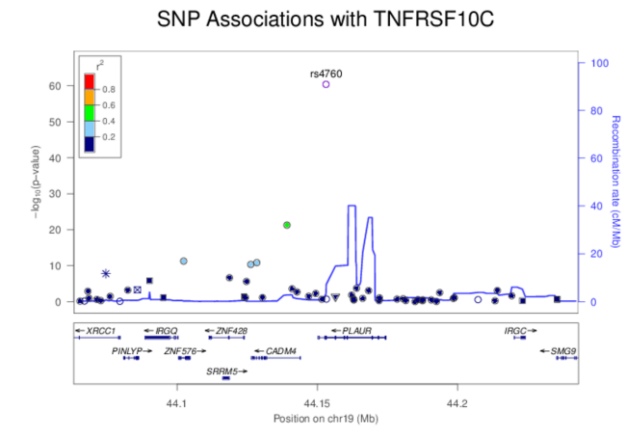

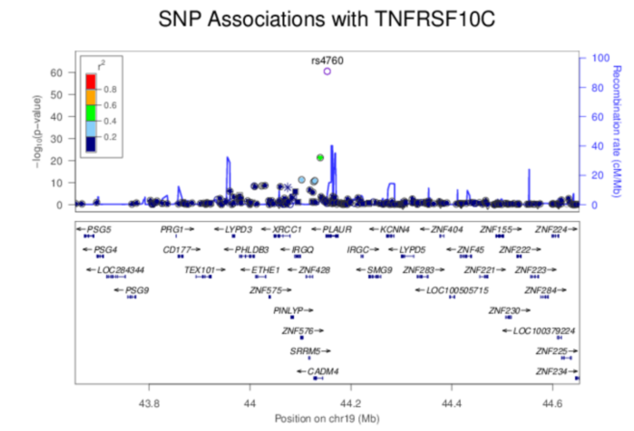

Vascular Endothelial Growth Factor A | **Gene Name: *VEGFA***

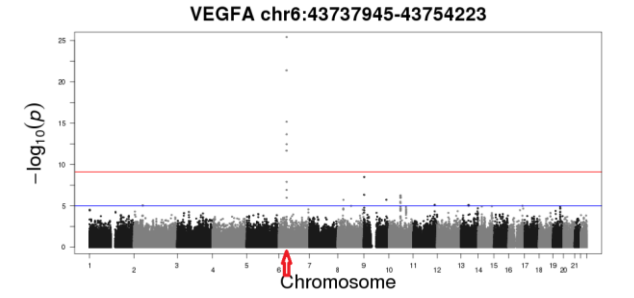

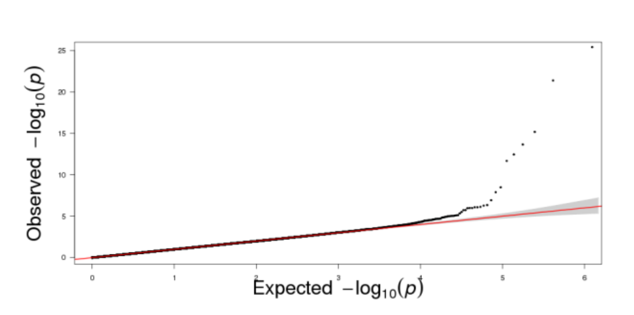

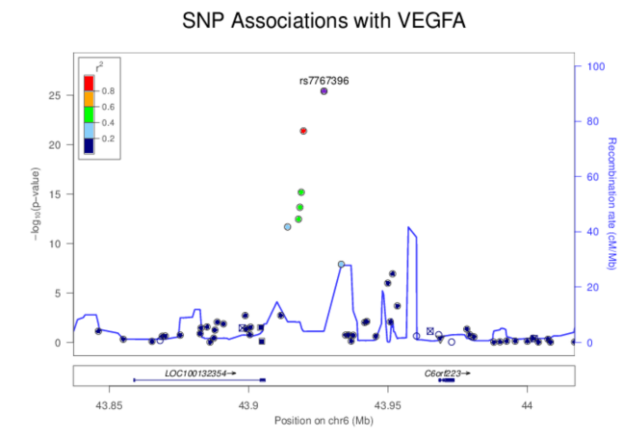

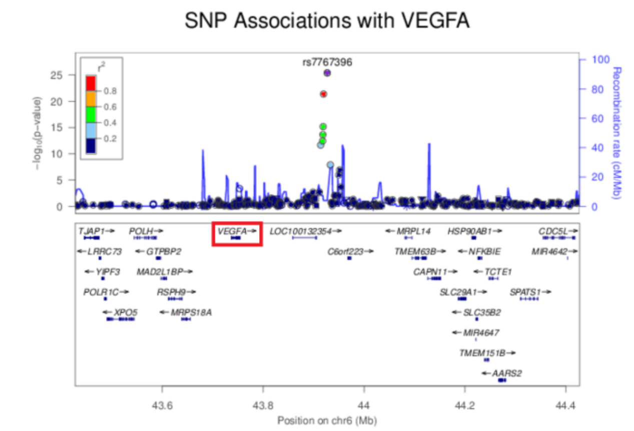

von Willebrand Factor | **Gene Name: *VWF***

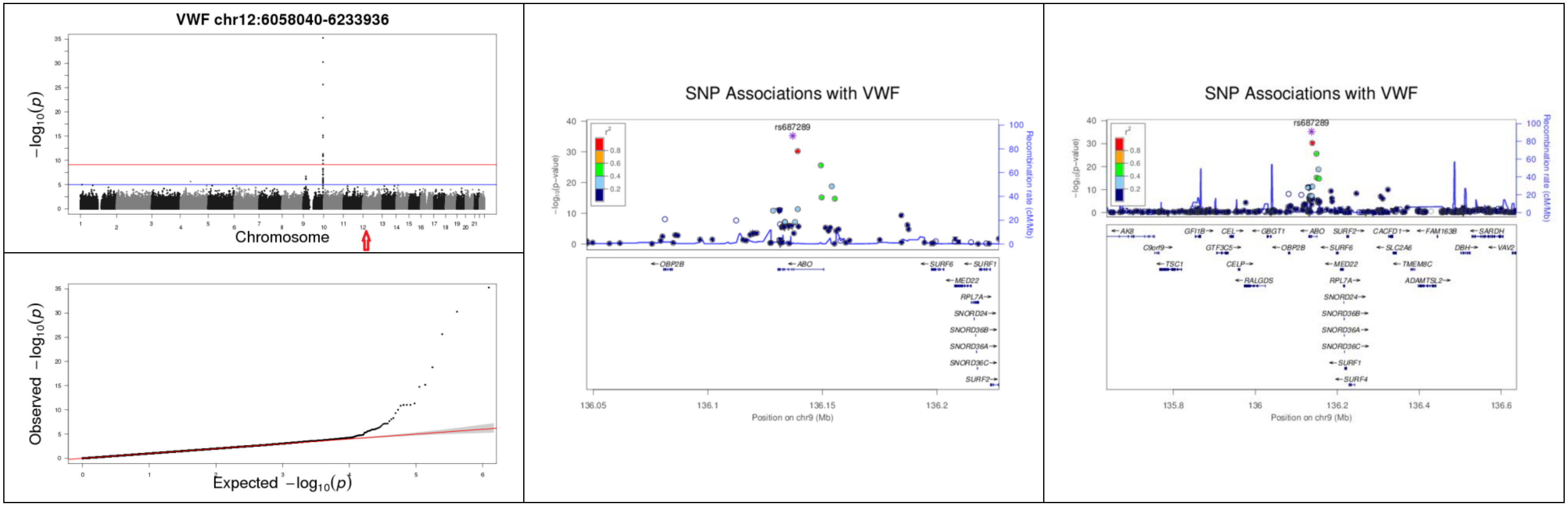
VWF chr12:6058040-6233936
Chromosome
Observed −log10(p)
Expected −log10(p)
SNP Associations with VWF
rs687289
Recombination rate (cM/Mb)
OBP2B
ABO
SURF6
SURF1
MED22
RPL7A
SNORD24
SNORD36B
SNORD36A
SNORD36C
SURF2
Position on chr9 (Mb)
SNP Associations with VWF
rs687289
AK8
GFI1B
CEL
GBGT1
ABO
SURF2
CACFD1
FAM163B
SARDH
C9orf9
GTF3C5
OBP2B
SURF6
SLC2A6
DBH
VAV2
TSC1
CELP
MED22
TMEM8C
RALGDS
RPL7A
ADAMTSL2
SNORD24
SNORD36B
SNORD36A
SNORD36C
SURF1
SURF4
Position on chr9 (Mb)
