# Supplementary material for: Common Genetic Polymorphisms Influence Blood Biomarker Measurements in COPD
Source: PLoS Genet. 2016 Aug 17;12(8):e1006011. doi: 10.1371/journal.pgen.1006011 (PMC4988780; doi:10.1371/journal.pgen.1006011)
Supplement: S5 Fig — Each dot represents a pQTL with P < 10−8. The x-axis denotes the location of the pQTL SNP and the y-axis denotes the location of the biomarker. The color of each dot denotes range of P-value as indicated in the legend. Dots more than 1 Mb from the identity line represent distant pQTL SNPs. Dots on the identity line represent local pQTLs. The bottom panel is useful to highlight the peak of pQTL SNPs located on chromosome 9 (ABO locus). (DOCX) [file pgen.1006011.s013.docx]

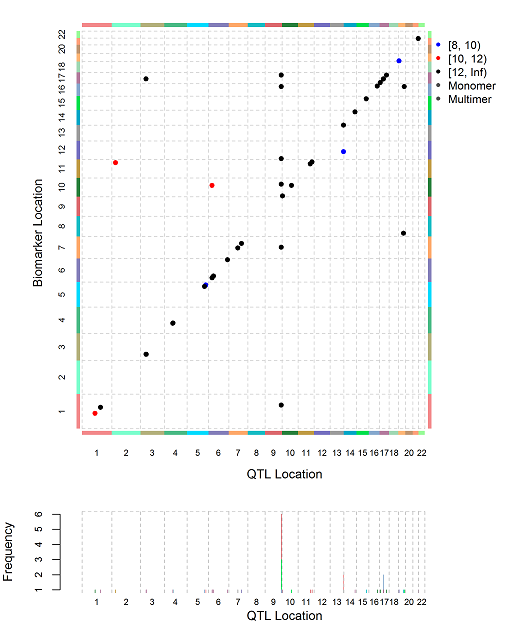


**S5 Fig.** Summary matrix of pQTLs. Each dot represents a pQTL with P < 10^-8^. The x-axis denotes the location of the pQTL SNP and the y-axis denotes the location of the biomarker. The color of each dot denotes range of P-value as indicated in the legend. Dots more than 1 Mb from the identity line represent distant pQTL SNPs. Dots on the identity line represent local pQTLs. The bottom panel is useful to highlight the peak of pQTL SNPs located on chromosome 9 (*ABO* locus).
