# Supplementary material for: Common Genetic Polymorphisms Influence Blood Biomarker Measurements in COPD
Source: PLoS Genet. 2016 Aug 17;12(8):e1006011. doi: 10.1371/journal.pgen.1006011 (PMC4988780; doi:10.1371/journal.pgen.1006011)
Supplement: S6 Fig — The highly significant pQTL SNPs (right panels) represent a distribution of minor allele frequencies similar in distribution to all SNPs in the study (left panels). SPIROMICS (top panel); COPDGene (bottom panel). (DOCX) [file pgen.1006011.s014.docx]

| 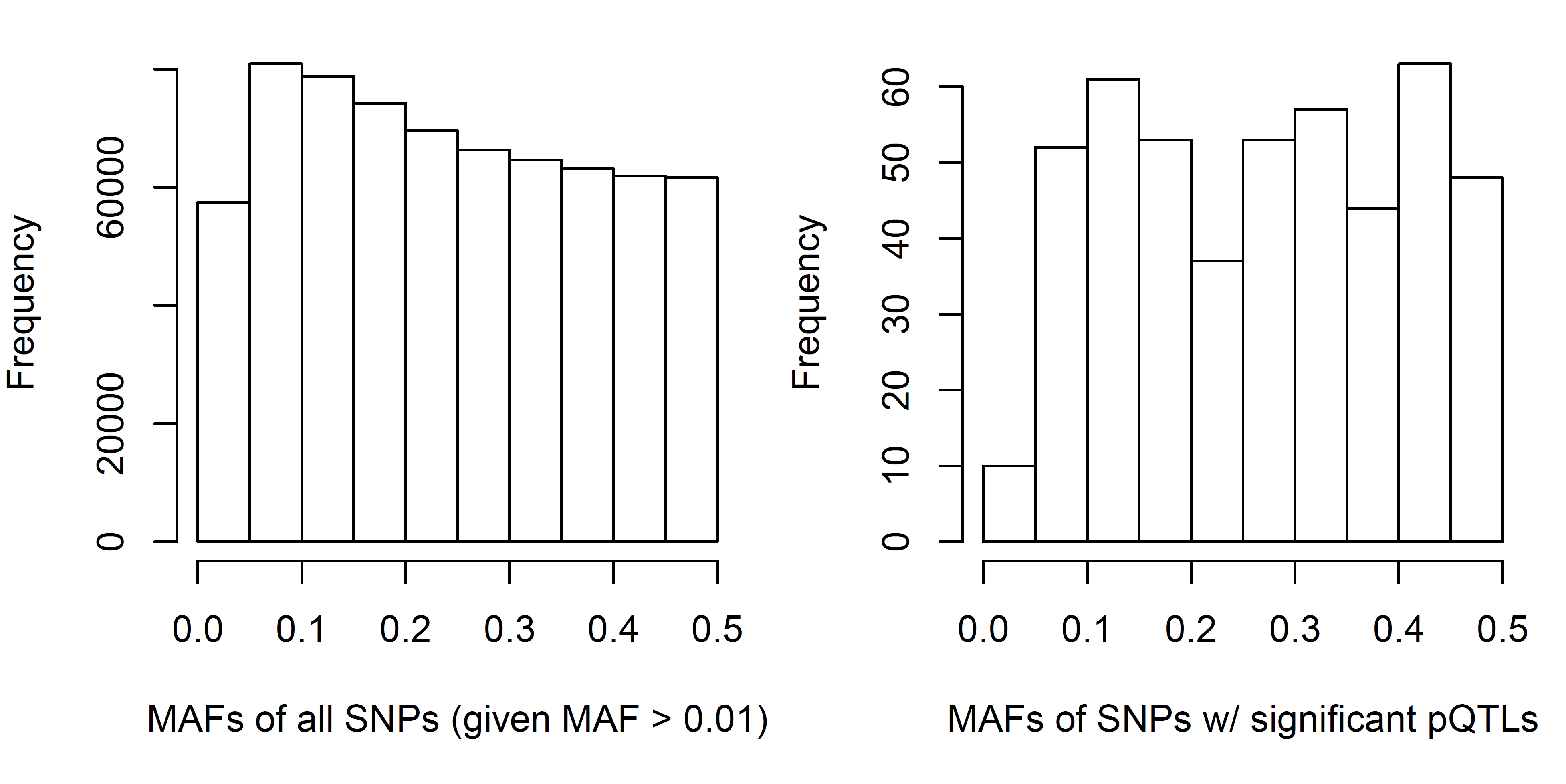 |
| --- |
| 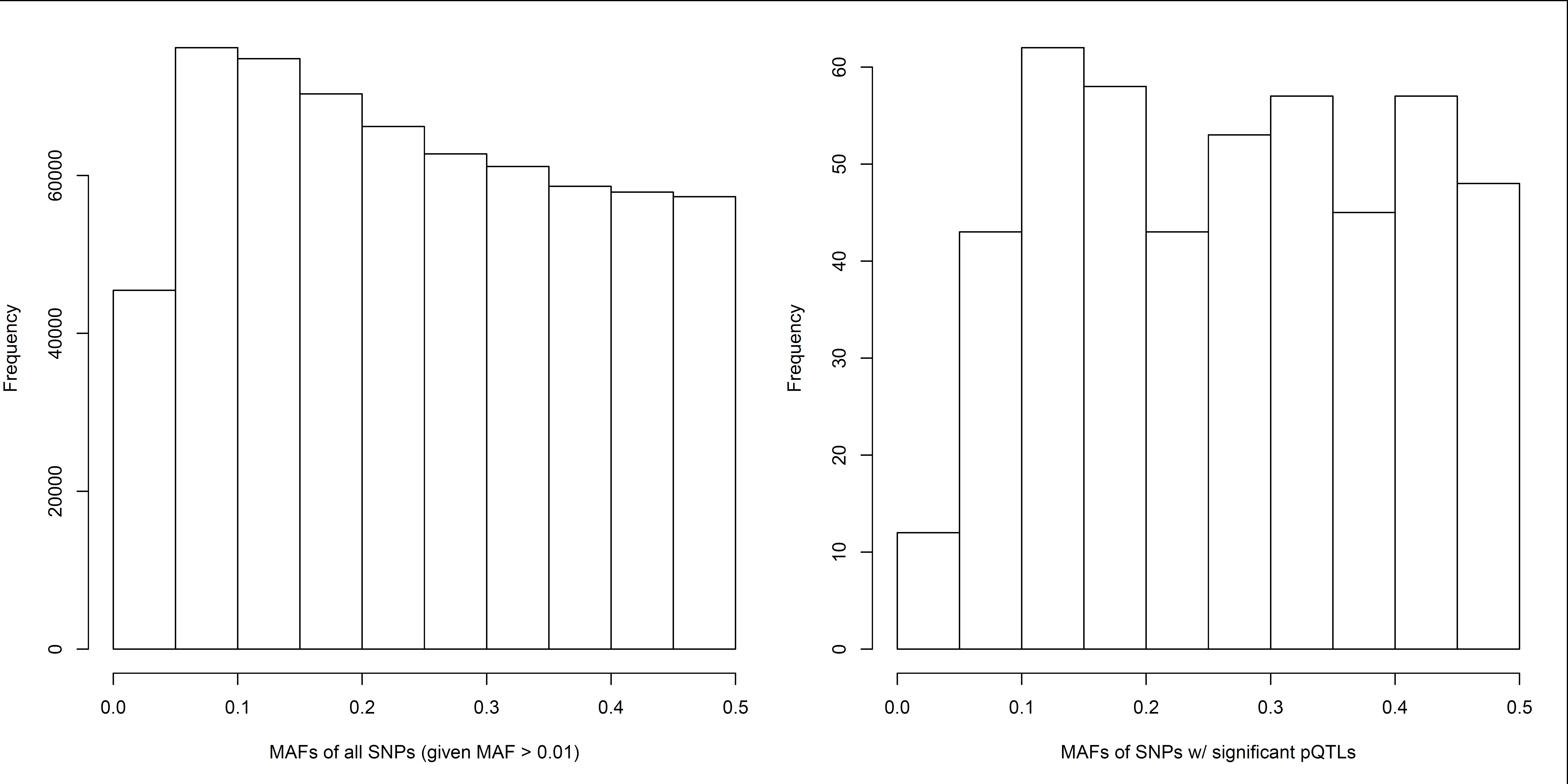 |
| **S6 Fig.** The highly significant pQTL SNPs (right panels) represent a distribution of minor allele frequencies similar in distribution to all SNPs in the study (left panels). SPIROMICS (top panel); COPDGene (bottom panel). |
