# Supplementary material for: Common Genetic Polymorphisms Influence Blood Biomarker Measurements in COPD
Source: PLoS Genet. 2016 Aug 17;12(8):e1006011. doi: 10.1371/journal.pgen.1006011 (PMC4988780; doi:10.1371/journal.pgen.1006011)
Supplement: S7 Fig — a)-b) For both cohorts, the percent variance explained (R2) was greater in the full model, which includes all covariates in addition to the top two independent SNP genotypes, compared to the genotype only model. The correlation (rho) between the two models was higher for COPDGene (0.92) compared to SPIROMICS (0.72). This indicates that utilized covariates are relatively more predictive of biomarker levels in SPIROMICS compared to COPDGene. c)-d) Percent variance explained correlated between COPDGene and SPIROMICS, with only genotype producing a stronger correlation (rho 0.88) compared to the full model (rho = 0.72). Thus, genotype in both cohorts have similar contributions to the percent variation in biomarker levels, while the contribution by the covariates is more variable and study dependent. (DOCX) [file pgen.1006011.s015.docx]

| 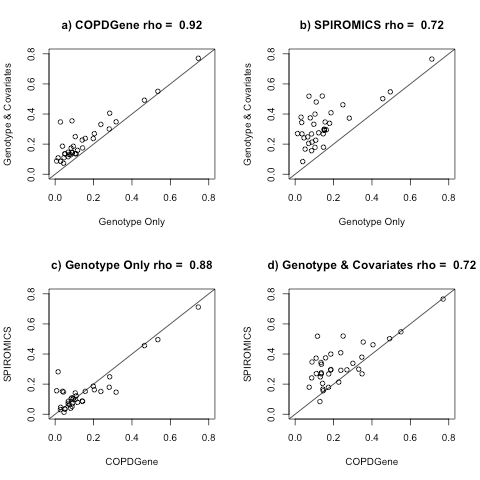 |
| --- |
| **S7 Fig.** Percent variance explained within and between studies.   a)-b) For both cohorts, the percent variance explained (R2) was greater in the full model, which includes all covariates in addition to the top two independent SNP genotypes, compared to the genotype only model.  The correlation (rho) between the two models was higher for COPDGene (0.92) compared to SPIROMICS (0.72).  This indicates that utilized covariates are relatively more predictive of biomarker levels in SPIROMICS compared to COPDGene.   c)-d) Percent variance explained correlated between COPDGene and SPIROMICS, with only genotype producing a stronger correlation (rho 0.88) compared to the full model (rho = 0.72) .  Thus, genotype in both cohorts have similar contributions to the percent variation in biomarker levels, while the contribution by the covariates is more variable and study dependent. |
