# Supplementary material for: Common Genetic Polymorphisms Influence Blood Biomarker Measurements in COPD
Source: PLoS Genet. 2016 Aug 17;12(8):e1006011. doi: 10.1371/journal.pgen.1006011 (PMC4988780; doi:10.1371/journal.pgen.1006011)
Supplement: S8 Fig — For 103 subjects, both gene expression and biomarker data were available from the COPDGene cohort. In those subjects, 80 biomarkers had available gene expression data in 199 probesets (multiple probesets may be available for a gene). For these 199 biomarker-gene expression pairs, there is a significant number of positive correlations (0.007, sign test) indicating that eQTLs (based on mRNA) can effect blood biomarker levels. (DOCX) [file pgen.1006011.s016.docx]

**S8 Fig.** Correlation between gene expression and biomarker level. For 103 subjects, both gene expression and biomarker data were available from the COPDGene cohort. In those subjects, 80 biomarkers had available gene expression data in 199 probesets (multiple probesets may be available for a gene). For these 199 biomarker-gene expression pairs, there is a significant number of positive correlations (0.007, sign test) indicating that eQTLs (based on mRNA) can effect blood biomarker levels.
