# Supplementary material for: Common Genetic Polymorphisms Influence Blood Biomarker Measurements in COPD
Source: PLoS Genet. 2016 Aug 17;12(8):e1006011. doi: 10.1371/journal.pgen.1006011 (PMC4988780; doi:10.1371/journal.pgen.1006011)
Supplement: S9 Fig — This figure represents an expanded version of Fig 3 in the main text. (DOCX) [file pgen.1006011.s017.docx]

|  |
| --- |
| **S9 Fig.** VEP analysis to evaluate the characteristics of the pQTL SNPs in comparison to eQTLs from various sources and the published findings from the NHGRI GWAS Catalog. A p-value cutoff of 10^-6^ was used to define the eQTL COPDGene to obtain enough examples to see trends across the different SNP types. This figure represents an expanded version of Figure 4 in the main text. The results of eQTL Twin study was reported by Wright et al. 2014 (Nat Genet. 2014;46(5):430-7). |
