# Supplementary material for: Common Genetic Polymorphisms Influence Blood Biomarker Measurements in COPD
Source: PLoS Genet. 2016 Aug 17;12(8):e1006011. doi: 10.1371/journal.pgen.1006011 (PMC4988780; doi:10.1371/journal.pgen.1006011)
Supplement: S10 Fig — When cell counts (eosinophils, basophils, neutrophils, monocytes, platelets, and red blood cells) were included in the regression models (Tobit or Linear as described in the methods) the significance of the pQTLs (X axis) did not vary significantly from results that did not include the CBC data (Y-axis). This was true for all pQTLs (left) and for the significant pQTLs (right). CBC data was only available from SPIROMICS and so these graphs represent SPIROMICS-only p-values. The correlation = 0.9854 for all SNP biomarker pairs and >0.999 for significant pQTL biomarker pairs. (DOCX) [file pgen.1006011.s018.docx]

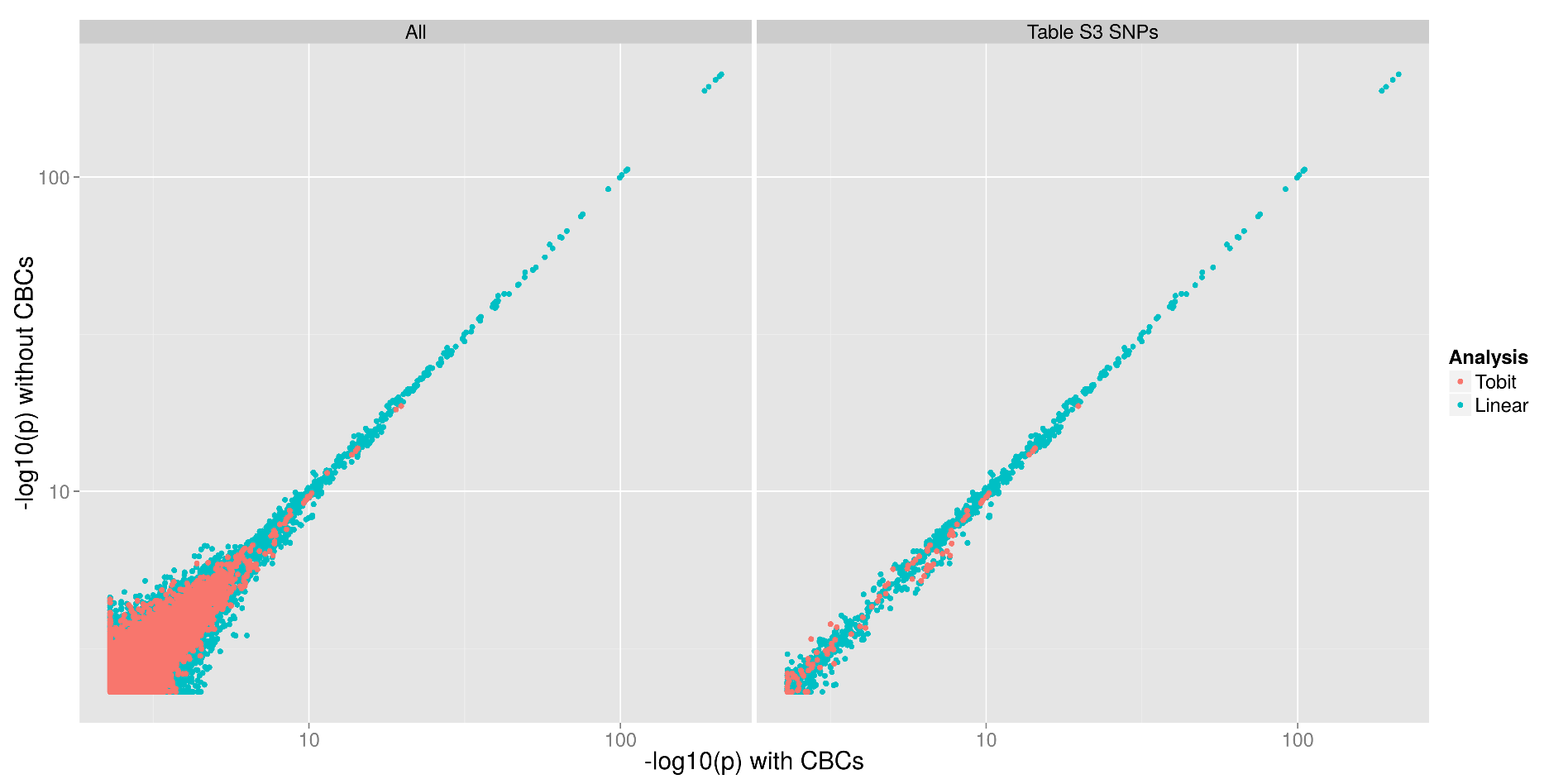


**S10 Fig.** Significant pQTLs are not affected by total blood cell counts (CBC). When cell counts (eosinophils, basophils, neutrophils, monocytes, platelets, and red blood cells) were included in the regression models (Tobit or Linear as described in the methods) the significance of the pQTLs (X axis) did not vary significantly from results that did not include the CBC data (Y-axis). This was true for all pQTLs (left) and for the significant pQTLs (right). CBC data was only available from SPIROMICS and so these graphs represent SPIROMICS-only p-values. The correlation = 0.9854 for all SNP biomarker pairs and >0.999 for significant pQTL biomarker pairs.
