# Supplementary material for: Common Genetic Polymorphisms Influence Blood Biomarker Measurements in COPD
Source: PLoS Genet. 2016 Aug 17;12(8):e1006011. doi: 10.1371/journal.pgen.1006011 (PMC4988780; doi:10.1371/journal.pgen.1006011)
Supplement: S11 Fig — (DOCX) [file pgen.1006011.s019.docx]

|  |
| --- |
| **S11 Fig.** Vitamin D binding protein measured by two different assay methods (polyclonal versus monoclonal) in selected SPIROMICS subjects who are GG or TT at rs7041. |
